# Summary of the Dutch Practice Guideline on Pregnancy Wish and Pregnancy in CKD

**DOI:** 10.1016/j.ekir.2022.09.029

**Published:** 2022-10-05

**Authors:** Margriet F.C. de Jong, Henk W. van Hamersvelt, Inge W.H. van Empel, Ellen J.W. Nijkamp, A. Titia Lely, the Dutch Guideline Working Group on Pregnancy in CKD

**Affiliations:** 1 Department of Internal Medicine, Division of Nephrology, University Medical Center Groningen, Groningen, the Netherlands; 2 Department of Nephrology, Radboud University Medical Center, Nijmegen, the Netherlands; 3 Department of Obstetrics and Gynecology, Radboud University Medical Center, Nijmegen, the Netherlands; 4 Department of Obstetrics and Gynecology, Utrecht University Medical Center, Utrecht, the Netherlands

**Keywords:** CKD, fetal outcome, kidney disease, maternal outcome, practice guideline, pregnancy

## Introduction

### Background

**Figure 1:**
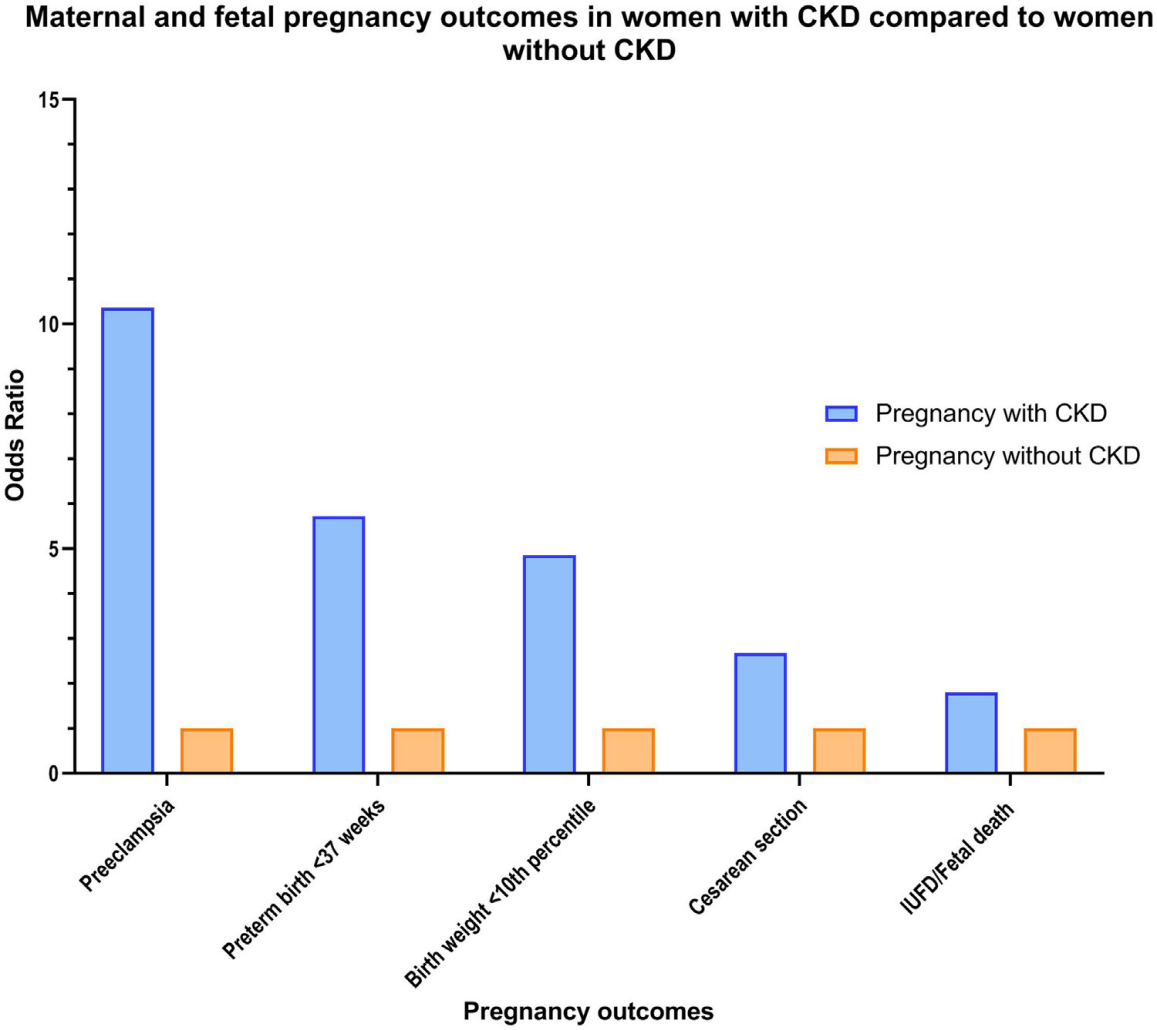
Maternal and fetal pregnancy outcomes in women with CKD compared to women without CKD. CKD, chronic kidney disease; IUFD, intrauterine fetal death.

**Figure 2:**
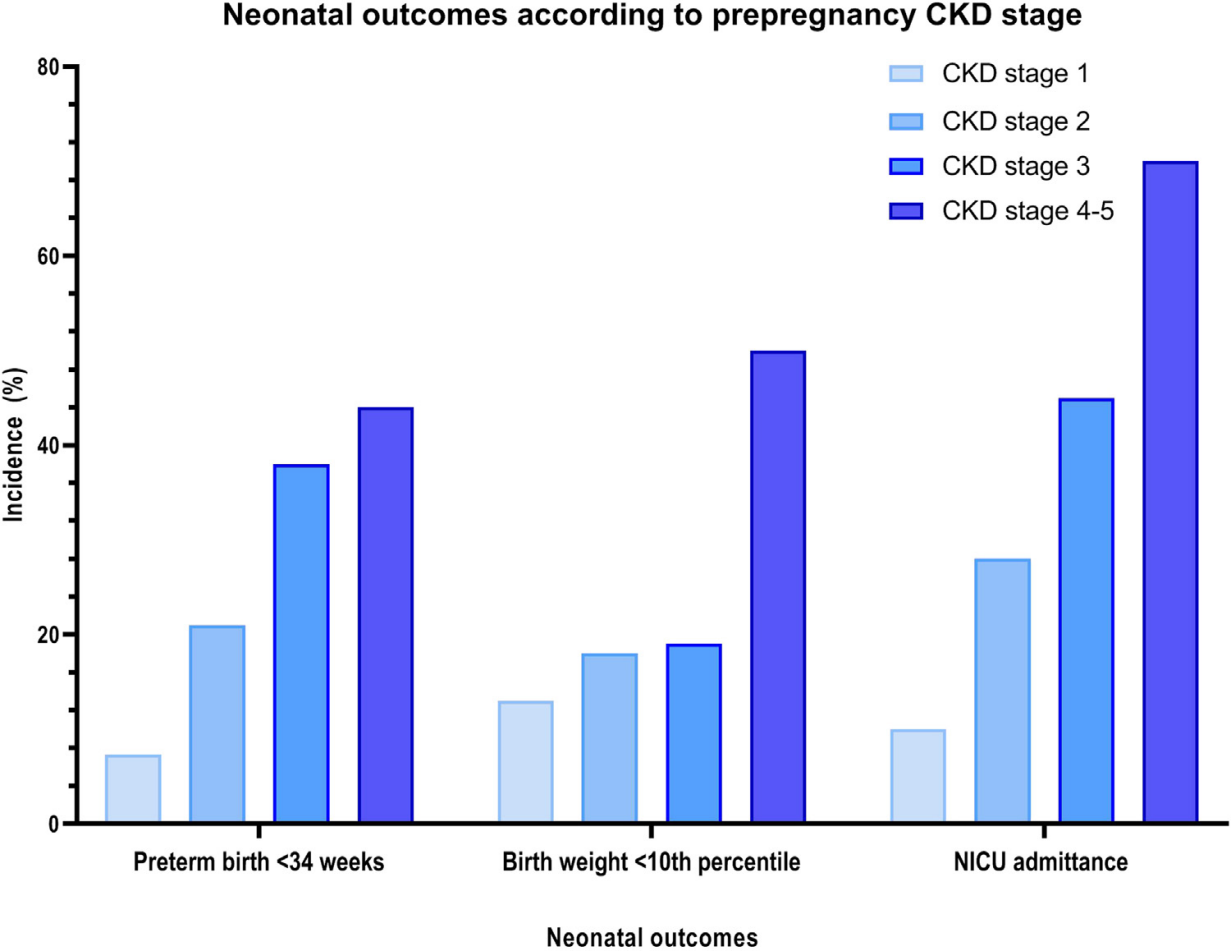
Neonatal outcomes according to prepregnancy CKD stage. CKD, chronic kidney disease; NICU, neonatal intensive care unit.

The number of women with chronic kidney disease (CKD) desiring a pregnancy is increasing. First, it is because of the increasing maternal age with the inherent higher incidence of CKD. Second, it is because of improved treatment options and higher numbers of kidney transplantations leading to increased number of fertile women with CKD for whom pregnancy can be a safe option. Furthermore, due to improved preconception care and multidisciplinary care before, during, and after pregnancy, maternal and fetal outcomes have improved. Nevertheless, preexisting CKD has negative effects on maternal and fetal outcomes (Figures 1 and 2). Patients with CKD or a kidney transplant and their offspring have an increased risk for pregnancy complications such as temporary or permanent kidney function decline, hypertension, preeclampsia, preterm birth, and intrauterine growth restriction.^1^, ^2^, ^3^, ^4^, ^5^, ^6^, ^7^, ^8^, ^9^

### Aims

**Figure 3:**
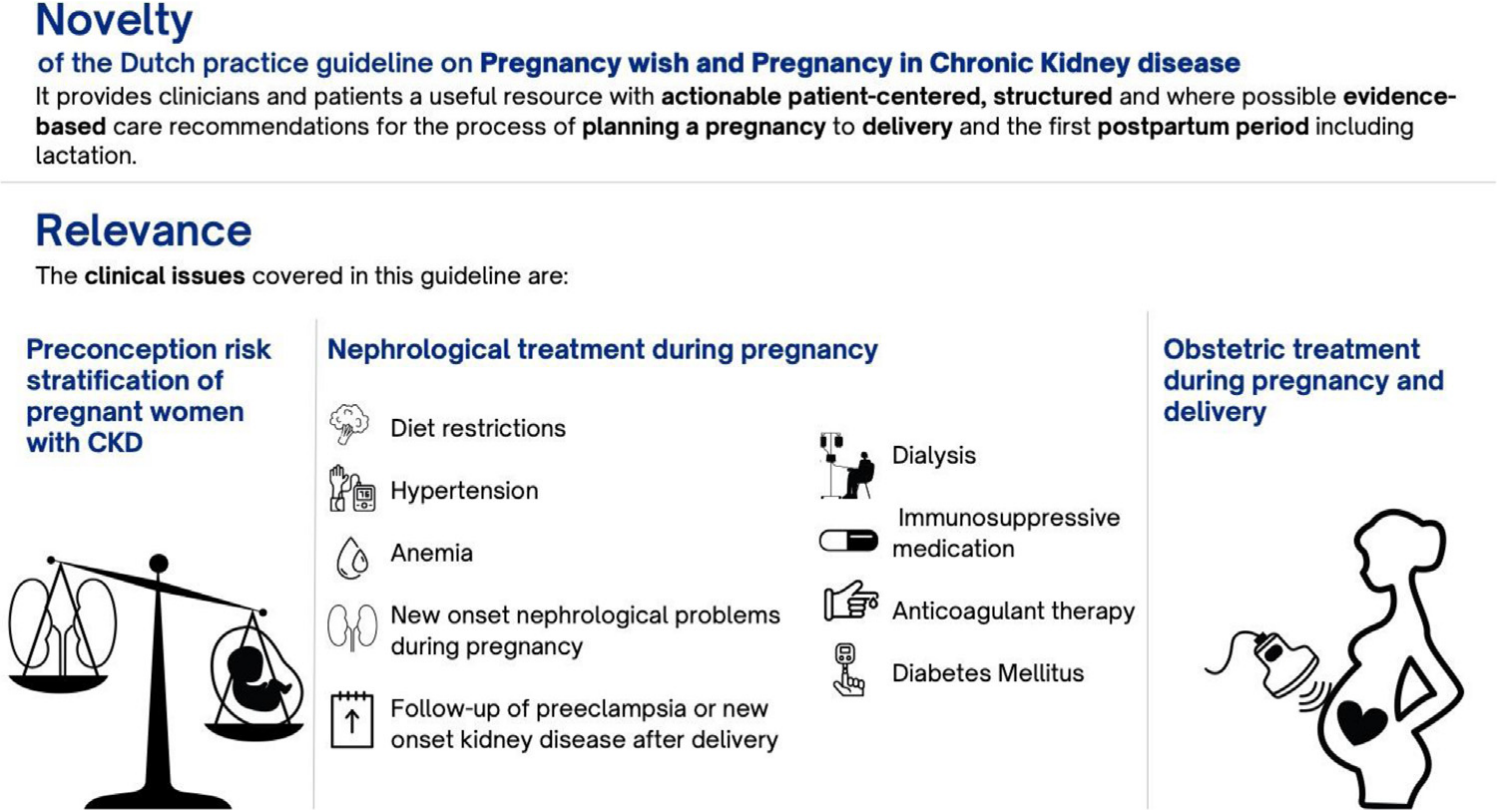
Novelty and relevance of the Dutch practice guideline on Pregnancy Wish and Pregnancy in CKD.

The aim of this guideline is to provide structured and where possible, evidence-based standard of care for patients with CKD throughout the process of planning a pregnancy to delivery and the early postpartum period including lactation (Figure 3).

### Scope

The clinical issues covered in this guideline include the following (Figure 3):

Part 1: Preconception risk stratification of pregnant women with CKD

1.1 Risk stratification
1.2 Preconception counseling
1.3 Preconception genetic counseling

Part 2: Nephrological treatment during pregnancy

2.1 Diet restrictions
2.2 Hypertension
  2.2.1 Target blood pressure before conception and during pregnancy with and without proteinuria
  2.2.2 Diuretics during pregnancy
  2.2.3 Safety of antihypertensive drugs used in pregnancy
  2.2.4 Antihypertensive drugs and diet interventions in patients with proteinuria preconceptionally
  2.2.5 Antihypertensive drugs during lactation
2.3 Anemia
  2.3.1 Target values of iron parameters
  2.3.2 Intravenous iron preparations
  2.3.3 Treatment with recombinant human erythropoietin (rhEPO)
  2.3.4 Blood transfusion
2.4 New onset nephrological problems during pregnancy
  2.4.1 Diagnostics of new nephrological problems (proteinuria/nephrotic syndrome/ thrombotic microangiopathy)
  2.4.2 Differentiation between preeclampsia and kidney disease
  2.4.3 Indication for kidney biopsy
2.5 Dialysis
2.6 Immunosuppressive medication
2.7 Anticoagulant therapy
2.8 Diabetes mellitus
2.9 Follow-up of preeclampsia or new onset kidney disease after delivery

Part 3: Obstetric treatment during pregnancy

3.1 Advanced ultrasound investigation
3.2 Decreasing preeclampsia risk
3.3 Delivery plan

## Executive Summary

The Dutch practice guideline on “Pregnancy Wish and Pregnancy in Patients with CKD” was developed by a multidisciplinary working group composed of all relevant specialisms and representatives of patients, and published in the Dutch medical guideline database in 2021. Guideline recommendations are based on available relevant studies and appraisal of the quality of the evidence. The strength of recommendations is based on the “Grading of Recommendations Assessment, Development and Evaluation” (GRADE) approach. Scope of the guideline was determined by clinical issues, such as preconception (genetic) counseling, diet restrictions, treatment of hypertension and anemia, immunosuppressive medication, and the obstetric treatment. The goal of the guideline is to provide clinicians and patients a useful resource with actionable patient-centered, structured, and where possible evidence-based care recommendations for the process of planning a pregnancy to delivery and the early postpartum period including lactation. Finally, knowledge gaps for each clinical issue are described.

## Lay Summary

The Dutch practice guideline on “Pregnancy Wish and Pregnancy in Patients with CKD” was developed by a multidisciplinary working group composed of all relevant specialisms (nephrologists, gynecologists, and clinical geneticist) and representatives of patients. It was published in the Dutch medical guideline database in 2021. Guideline recommendations are based on available relevant studies and appraisal of the quality of the evidence. The strength of recommendations is based on the GRADE approach. The scope of the guideline was determined by clinical issues. Examples of the issues include the following:

- discussing the pregnancy wish before an actual pregnancy, review of risk factors for pregnancy complications and worse kidney outcomes, need for referral to a clinical geneticist, and a medication review,

- diet restrictions,

- treatment of high blood pressure and anemia,

- immunosuppressive medication, and

- the obstetric treatment.

The goal of the guideline is to provide clinicians and patients a useful resource with actionable patient-centered, structured and where possible, evidence-based care recommendations. This includes the whole process of planning a pregnancy to delivery and the early postpartum period including lactation. Finally, knowledge gaps for each clinical issue are described.

## Methods

For development of this guideline a multidisciplinary working group was composed of all relevant specialisms and representatives of patients. The scope of the guideline was determined by clinical issues that were framed by a research question and background. Literature searches were conducted by literature specialists using specific search terms related to each of the issues covered. The research question, background, search and select method, results, conclusion, rationale, and recommendations were described for each clinical issue. Strength of evidence was determined by the GRADE method.^10^^,^^11^ When GRADE was not possible because studies found by the literature search did not meet the inclusion criteria, studies were described in the analysis of the literature if relevant for the direction of the recommendations. For this publication, recommendations were officially translated from Dutch by a forward and backward method. Other parts of the Dutch guideline were (somewhat shortened) translated by the authors. The full guideline is reported in the Supplementary Materials.

## Recommendations

### Preconception Risk Stratification and Counseling

#### Risk Stratification (no GRADE)

1. Preconception consultation of women with CKD stage 3 or higher, or with a kidney transplant, or with a systemic autoimmune disease (regardless of the use of immunosuppressants) should preferably be performed in a university hospital by a maternal-fetal medicine specialist and a nephrologist. Both specialists should be experienced in supervising pregnant women with CKD.
  • For women with CKD stage 1 or 2 without a kidney transplant or systemic autoimmune disease, the working group suggests that it is useful to receive counseling at a university hospital.
2. Pregnant women with CKD stage 1 or 2 without a kidney transplant and without systemic autoimmune disease needing immunosuppressants may receive specialist care in a general hospital. This care should be given by an obstetrician and a nephrologist with affinity for pregnancy in patients with CKD.
  If the patient’s condition or renal function deteriorates or fetal complications (are likely to) develop, the attending physician should consult with the specialized team at a university hospital and refer the patient without hesitation.

3. Pregnant women with CKD stage 3 or higher should preferably be treated in a university hospital by a maternal-fetal medicine specialist and a nephrologist who should both be experienced in the treatment of pregnant patients with CKD.
4. Referral to a university hospital is strongly recommended for patients with a kidney transplant, patients with CKD due to a systemic autoimmune disease needing immunosuppressants and dialysis patients.

### Preconception Counseling (no GRADE)

1. Recommend patients with CKD who do not want to become pregnant (yet) to use safe and effective contraception. Actively inquire into the desire to conceive.
2. Consultation with a CKD patient who wants to conceive, to be conducted by a nephrologist and maternal-fetal medicine specialist, should at least address the following aspects:
  • the possible effects of pregnancy on the underlying kidney disease and assessment of the risk of transient and/or permanent loss of renal function
  • timing of pregnancy
  • the effects of CKD on pregnancy, specifically the risks of pregnancy complications such as hypertension, preeclampsia, intrauterine growth restriction, and (iatrogenic) premature birth (Figures 1 and 2)
  • if the woman becomes pregnant, her life expectancy and quality of life (especially in case of end-stage renal failure), so that the woman and her partner can make an informed choice about how to fulfill their desire to have children. Those close to the patient and her partner can also be involved in this process.
  • the potential risk of teratogenic medication and, if possible, recommend discontinuation or switch to a safe alternative
  • periodical evaluations of the likelihood that patients (male/female) with a latent desire to have children may have hereditary kidney disease; do not hesitate to refer them to a clinical geneticist
  • if the kidney disease is hereditary and carries an elevated risk of inheritance for future children, inquire if the couple would like to be informed about the various options for fulfilling their desire to have children without the genetic disorder
  • general preconception advice, including stopping smoking, alcohol and/or drugs, weight reduction if BMI >25 kg/m^2^, and prophylactic use of daily 400 mcg of folic acid before conception.
3. The disease of patients with CKD with a desire to conceive should be stable; if necessary, regulate their blood pressure (<130/80 mm Hg).
4. Formulate a multidisciplinary treatment plan depending on the patient’s condition and an assessment of the risk of complications.
5. Also recommend that the patient takes acetylsalicylic acid from the twelfth week of pregnancy (after last menstruation) to reduce the risk of hypertensive disorders during pregnancy.

#### Preconception Genetic Counseling (no GRADE)

1. At an early stage, assess whether patients with CKD (male/female) with a potential or actual desire to have children may have hereditary kidney disease.
2. Do not hesitate to refer patients with CKD (male/female) with a potential desire to have children to a clinical geneticist for counseling and, if necessary, additional testing. At least consider referral if:
  • patients are undiagnosed or if there is doubt about the current diagnosis; this includes:
    ○ patients with a severe manifestation of their CKD, for example:
      ▪ symptomatic CKD at a young age (<20 years old),
      ▪ CKD stage 4 or 5 in a patient younger than 40 years
  • patients with a positive family history of primary kidney disease or hereditary disorders with renal involvement such as atypical hemolytic uremic syndrome
  • adult patients with a confirmed hereditary disorder if they have not had preconception counseling or if the preconception counseling was more than 5 years ago
  • patients who desire prenatal diagnostics:
    ○ who consulted with a clinical geneticist more than 2 years ago
    ○ whose prenatal diagnostics report or instructions are not explicit enough
    ○ who did not receive genetic counseling at the center where the prenatal diagnostics will be conducted.
  In the above cases, contact the clinical geneticist at the center where the prenatal diagnostics will be conducted to verify if a referral is indicated.
  • Patients with a confirmed hereditary disorder who may wish to be eligible for preimplantation genetic diagnostics (In the Netherlands, patients will always have to be referred for such preimplantation genetic diagnostics via a clinical geneticist).

### Nephrological Treatment During Pregnancy

#### Diet Restrictions (no GRADE-Very Low GRADE)

1. Check sodium excretion in the 24-hour urine of pregnant patients with CKD (nondialysis (ND)) and limit their daily salt intake to a maximum of 6 grams (= 100 mmol of sodium chloride equivalent to 2400 mg of sodium) as recommended for all patients with CKD. (very low GRADE)
2. For pregnant women with CKD and (expected) high degree of sodium retention during pregnancy (manifesting as excessive weight gain combined with edema and/or hypertension), consider further limiting the daily salt intake to 3 grams (1200 mg sodium) during pregnancy. Refer patients to a dietician for this purpose. (very low GRADE)
3. Regularly check the salt excretion of pregnant women with CKD and a good indication for salt restriction using 24-hour urine or the sodium-to-creatinine ratio (mmol sodium/10 mmol creatinine) in a spot urine sample. (no GRADE)
4. Continue or start pregnant patients with CKD and a good nephrological indication for protein restriction on a diet of 0.8 g/kg ideal weight per day throughout pregnancy and, therefore, be reluctant to increase protein intake in the second and third trimesters as recommended for healthy pregnant women. This recommendation does not apply to dialysis patients. (no GRADE)
5. For kidney patients with a (highly) advanced CKD stage (G3b-G5ND), consider prescribing a (stricter) protein-restricted diet of less than 0.8 g/kg ideal weight per day during pregnancy to maintain their serum urea level at <17 mmol/l in order to prevent polyhydramnios, and thereby attain a longer gestational age and a higher birth weight. (no GRADE)
6. Consider supplementing this strictly protein-restricted diet with amino acid supplements to ensure an equivalent daily protein intake of at least 0.8 grams/kg ideal weight/day by referring patients to a dietician with expertise in this area. (no GRADE)
7. Consider regularly checking the urea level in 24-hour urine or a spot urine sample to estimate protein intake and thereby assess dietary compliance, and avoid excessively low protein intake. (no GRADE)

#### Hypertension (No GRADE–Low GRADE)

1. When treating hypertension in patients with CKD and a desire to conceive, aim preconceptionally for blood pressure <130/80 mm Hg, regardless of the proteinuria level. (no GRADE)
2. During pregnancy, only initiate antihypertensive treatment in patients with CKD (with or without albuminuria) who have not used antihypertensives before or during pregnancy if their blood pressure is higher than 140/90 mm Hg on repeated measurements. (no GRADE)
3. In patients with CKD who already used antihypertensives before conception, only intensify the antihypertensive regimen during pregnancy if their blood pressure is higher than 140/90 mm Hg on repeated measurements. (no GRADE)
4. For patients with CKD (with or without albuminuria) who use antihypertensives during pregnancy, aim at a systolic blood pressure between 130 mm Hg and 140 mm Hg and a diastolic blood pressure between 80 mm Hg and 90 mm Hg. (no GRADE)
5. After birth, aim at a consistent blood pressure ≤130/80 mm Hg in patients with CKD. (no GRADE)
6. Consider prescribing diuretics to patients with CKD with a desire to conceive only if:
  • their blood pressure does not reach the desired target of <130/80 mm Hg with the central alpha-blocker methyldopa, beta blockers and/or calcium channel blockers due to (possible) sodium retention caused by their CKD;
  • their proteinuria is higher than 0.5 grams/day in order to reduce the likelihood of sodium retention and associated hypertensive disorders during pregnancy. (low GRADE)
7. Do not prescribe diuretics to patients with CKD before conception:
  • to prevent pregnancy-specific hypertensive disorders (including preeclampsia),
  • as preferred treatment of (essential) hypertension and nephrogenic hypertension without relevant proteinuria. (low GRADE)
8. Only start thiazide or loop diuretics in pregnant patients with CKD if there is a strict indication. (low GRADE)
9. Avoid starting diuretics if preeclampsia is suspected because preeclampsia is usually due to excessive vasoconstriction with intravascular underfilling. This may be aggravated by diuretics, with a potentially negative effect on placental perfusion. (no GRADE)
10. Patients with CKD should continue to take thiazide diuretics during pregnancy if they were started before pregnancy *and* there is no good alternative. (low GRADE)
11. Only if there is a strict indication for potassium-sparing diuretics, consider prescribing amiloride during pregnancy. Avoid triamterene and aldosterone antagonists (e.g. spironolactone) during pregnancy due to the risk of severe teratogenic effects. (no GRADE)
12. Choice of antihypertensives (Table 1; no GRADE):
  • When prescribing methyldopa preconceptionally, be aware of the possibility of amenorrhea due to hyperprolactinemia and the central side effects such as lethargy, dizziness and sadness, making it a less attractive drug in patients with (a history of) depression.
  • If a beta-blocker must be prescribed, select an agent that is considered safe (labetalol, pindolol, metoprolol, or bisoprolol).
  • Be very careful when prescribing short-acting dihydropyridine calcium antagonists such as direct-acting nifedipine and starting high doses in short periods.
  There is only limited evidence that dihydropyridine calcium antagonists such as nifedipine and amlodipine are safe in the first trimester. However, case series and extensive obstetrical experience have revealed no serious problems. Such agents can be prescribed preconceptionally if there are no good alternatives.
  • Stop renin-angiotensin system (RAS) inhibitors either preconceptionally or early in the first trimester, certainly before 8 weeks of amenorrhea, because RAS inhibitors can cause severe congenital defects and oligohydramnios, due to fetal anuria induced by fetal hypotension.
  • Only recommend continuing RAS inhibitors in women with a desire to conceive if they are sufficiently aware of needing to stop taking the drug immediately if 2 consecutive pregnancy tests are positive (no later than 8 weeks of amenorrhea) and have a strong indication, that is:
    ○ diabetic nephropathy with at least moderate albuminuria (A2, ACR ≥3 mg/mmol)
    ○ CKD and severely elevated albuminuria (A3, ACR ≥30 mg/mmol, corresponding to proteinuria ≥0.5 g protein/24 hours or protein/creatinine ratio ≥0.5 g/10 mmol creatinine).

**Table 1:** Considerations for use of antihypertensive agents before and during pregnancy

Antihypertensive agen		Safe in pregnancy	Considerations for the mother	Considerations for the child
Group	Agent			
Central acting sympathomimetic agents	Methyldopa	Yes	-preconceptional amenorrhoea due to hyperprolactinemia -drowsiness, tiredness, depression -less effective in severe hypertension	Safe, also in long-term follow-up
Betablocking agents	In general		-careful in severe asthma and liver function disorder	When used during delivery: small increased risk of hypoglycemia and very sporadic bradycardia. Consider monitoring postpartum, certainly with high dosages
	Alfa and betablocking agent (labetalol)	Yes	-agent with most experience during pregnancy -can also be given IV -orthostatic hypotension, mainly with dosages >1200 mg/d -nightmares	Possibly risk 1:500 of multicystic kidney dyplasia
	Lipophilic betablocking agents (metoprolol, bisoprolol)	Yes	-safe alternatives for labetalol -nightmares	
	Lipophilic betablocking agents with partial agonist activity (pindolol)	Yes	-pindolol safe alternative for labetalol -nightmares	Possibly positive effect on placenta perfusion
	Hydrophilic betablocking agents (atenolol, sotalol)	?		Possibly fetal growth retardation
Dihydropyridine calcium antagonists	In general	-limited evidence for safety in 1st trimester -safe in 2nd and 3rd trimester	-very effective for treatment of preeclampsia in 2nd and 3rd trimester -frequently used tocolytic agent -be careful with high dosages in short time interval -edema -uterus atony	
	Nifedipine		-be very careful with short acting variants	
	Amlodipine		-advantage once daily dosing	
Loop and thiazide diuretics	Preconceptional	-safe when used preconceptionally -can be continued during pregnancy	Possible indications preconceptionally: -insufficient effect of other agents or -preconceptional (glomerular) proteinuria >0.5 g/d Side effects: -nausea/vomiting -hypokalemia -gout	No association with congenital defects
	During pregnancy	Start only with strict indication in 2nd half of pregnancy	Contraindication is suspicion of preeclampsia. Possible indications: -fluid overload in severe renal insufficiency and/or nephrotic syndrome -heart failure	
	Hydrochlorothiazide	Safe when started before conception	Agent with most obstetric experience	
	Furosemide	Probably safe during pregnancy in case of fluid overload	Most effective for sodium retention in renal insufficiency	
Potassium sparing agents	Amiloride	Very limited evidence for safety in 1st trimester	Possible indications: -hypokalemia despite diet and suppletion -nephrotic range proteinuria	Risk of congenital defects unknown
	Triamterene	No, it is a folic acid antagonist		Risk of schizis, neural tube, cardiovascular and urinary tract defects
	Spironolactone	No, because of antiandrogenic effects		Feminisation of male fetus
Renin-angiotensin inhibitors	-ACE inhibitors -angiotensin receptor blocking agents -renin inhibitors	-until 8 weeks amenorrhoea probably safe -from 8 weeks amenorrhoea teratogenic and fetotoxic	Consider continuation preconceptionally: 1. diabetic nephropathy with at least moderately increased albuminuria 2. chronic kidney disease with severely increased albuminuria or proteinuria ≥0.5 g protein/24h -Only if pregnant woman can be well instructed to stop after positive pregnancy test.	Severe congenital defects and oligohydramnios because of fetal anuria

IV, intravenous.
13. In patients with CKD with proteinuria who develop nephrotic range proteinuria (>3 grams/day) during pregnancy, consider one or more of the following measures:
  • further restricting the dietary sodium intake to approximately 1200 mg/day
  • limiting the daily protein intake to 0.6 to 0.7 grams/kg ideal weight, supplemented with amino and keto acids equivalent to 0.2 to 0.3 grams of protein/kg ideal weight
  • adding the potassium-sparing diuretic amiloride in patients who already use a diuretic and develop (severe) sodium retention from the twentieth week onwards, because of its beneficial effect on sodium retention caused by proteinuria
  • starting with a dihydropyridine calcium antagonist only if the patient’s systolic blood pressure is above 140 mm Hg or diastolic blood pressure is above 90 mm Hg on repeated measurements despite the maximum dose of the thiazide diuretic and beta-blocker. (no GRADE)
14. For women who need an antihypertensive during lactation, the potential advantages and disadvantages of the various agents must be considered.
  • When there is proteinuria >0.5g/24h or diabetic nephropathy, start with enalapril if antihypertensive treatment is indicated, as soon as possible after birth.
  • For women with CKD who need a beta-blocker during lactation, choose a beta-blocker with favorable pharmacokinetic properties (high protein binding, low kidney excretion: labetalol, metoprolol, pindolol, or propranolol) because these can be safely used during lactation.
  • Avoid prescribing alpha-blockers during lactation because it is largely unknown whether these drugs find their way into breast milk and the neonatal risks are still unknown. (no GRADE)

#### Anemia (No GRADE)

1. Aim at a ferritin level >80 mcg/l to 500 mcg/l in pregnant patients with CKD with intact renal function (estimated glomerular filtration rate [eGFR] >60; stage G1-2) and anemia.
2. Aim at a ferritin level >200 mcg/l to 500 mcg/l in pregnant patients with CKD with impaired renal function (eGFR <60; stage G3-5ND) and anemia.
3. In principle, do not prescribe iron in pregnant patients with CKD with ferritin level >500 mcg/l and/or transferrin saturation >30%.
4. Aim at a transferrin saturation between 30% and 50% and a ferritin level >300 mcg/l to 800 mcg/l in pregnant dialysis patients with anemia.
5. Start pregnant patients with CKD with iron-deficiency anemia on oral iron supplements; aim at a ferritin level >80 mcg/l.
6. Consider starting pregnant patients with CKD with even mild iron deficiency on such supplements even if their hemoglobin (Hb) is normal.
7. In the second and third trimesters, switch to parenteral iron supplements if the target levels cannot be achieved with oral agents and regularly check Hb and iron levels.
8. If pregnant patients with CKD must be given parenteral iron supplements, prescribe one of the modern, stable iron supplements such as iron carboxymaltose (Ferinject) with a maximum dose of 1000 mg (up to 15 mg/kg/dose) or iron isomaltoside (Monofer/Diafer) in doses of up to 1000 to 2000 mg (maximum 20 mg/kg/dose).
9. If one of the older, less stable iron supplements such as iron sucrose (Venofer) is selected for pregnant hemodialysis patients, give a low dose of up to 62.5 to 100 mg during dialysis because of the possibility of fetal iron accumulation.
10. Aim for an Hb of 6.2 to 6.8 mmol/l (corresponding with an Ht of 30% to 35%) in pregnant patients with CKD (with or without dialysis), because the obstetrical outcomes are better if Hb >6.2 mmol/l.
11. Exclude iron, folic acid, and vitamin B12 deficiencies and adequately treat any deficiencies before considering prescribing rhEPO.
12. Consider starting pregnant patients with CKD with (renal) anemia (Hb <6.2 mmol/l) on rhEPO. When doing so, always weigh the potential benefits for the well-being of the mother and the obstetrical outcome against the potential risks of vasoconstriction with aggravation of hypertension, particularly if Hb rises quickly.
13. If a rapid increase in Hb is desired, for example because labor is approaching, consider leukocyte-depleted, Parvovirus B19-free, and cEK-compatible erythrocyte transfusions as a good alternative to rhEPO. Because of the leukocyte depletion, these transfusions are CMV-free too.
14. Because of erythropoietin resistance during pregnancy, consider increasing the rhEPO dose for pregnant patients who already used rhEPO before pregnancy by 50% to 100% to achieve and/or maintain the Hb target value. Regularly review the rhEPO dose in the light of the patient’s Hb. For most patients, a maximum dose of twice the normal starting dose can be used as a safe dose.
15. For pregnant patients with CKD, perioperative transfusions should be given if Hb <4.5 mmol/l and considered if Hb <5.0 mmol/l in a stable situation. However, consider transfusions if Hb <6.0 mmol/l and major blood loss is expected (e.g., around birth) because the obstetrical outcomes are better if Hb >6.2 mmol/l.
16. Consider blood transfusions for pregnant dialysis patients who are classified as ASA 3 due to the dialysis if their Hb <6.0 mmol/l and it cannot be expected that Hb can be corrected within a few weeks with parenteral iron supplements and/or rhEPO. Always give blood transfusions if Hb < 6.0 mmol/l and major blood loss is expected (e.g., around birth).
17. When considering the need for transfusions, consider the risk of allosensitization in view of organ transplantation in the near or far future. Discuss the potential consequences of blood transfusions.

#### New Onset of Nephrological Problems During Pregnancy

Proteinuria/nephrotic syndrome/thrombotic microangiopathy (no GRADE)

1. In pregnant women showing a decline in eGFR and/or proteinuria >300 mg/day during the first half of their pregnancy, analyze the cause of the renal insufficiency using diagnostic tools that would also be used outside pregnancy (ultrasonography, urine tests, and, if indicated, specific immunological tests or a selectivity index).
2. Pregnant women with symptomatic urolithiasis should primarily receive conservative treatment. Be reluctant to apply retrograde urological interventions in the urinary tract due to the risks of inducing preterm labor and urosepsis.
3. Consider thrombotic microangiopathy that is not part of hemolysis, elevated liver enzymes and low platelets for women with acute kidney injury and hemolysis during pregnancy and after birth.

Differentiation between preeclampsia and kidney disease (no GRADE)

1. Do not use the soluble fms-like tyrosine kinase-1/ placental growth factor ratio in daily practice to distinguish between preeclampsia and (underlying) primary kidney disease because the available data is not yet sufficiently reliable.

Indication for kidney biopsy (no GRADE)

1. Do not refrain from a kidney biopsy in pregnancy if there is a strict indication to do so because the risks of a kidney biopsy under ultrasonographic guidance with an automatic biopsy device are the same for pregnant and nonpregnant women.
The main indications are:
  • severe nephrotic syndrome in the first or second trimester,
  • nephrotic syndrome with progressive renal failure in all trimesters,
  • suspicion of acute glomerulonephritis in patients with progressive renal failure with glomerular erythrocyturia with or without proteinuria,
  • suspicion of acute rejection of a kidney transplant.
2. In patients with postpartum persistent non-nephrotic range proteinuria without (progressive) renal failure, only perform a renal biopsy at least 6 months after the birth due to the high likelihood of spontaneous recovery of pregnancy-related glomerular abnormalities in this postpartum period.

#### Dialysis (No GRADE)

1. In hemodialysis patients who want to become pregnant, consider whether a kidney transplant is possible and whether pregnancy can be postponed until 1 year after transplantation because the pregnancy outcomes after kidney transplantation are significantly better than during hemodialysis.
2. Preferably treat pregnant dialysis patients with intensive hemodialysis instead of peritoneal dialysis.
3. Refer pregnant dialysis patients to a center that has a multidisciplinary team experienced in treating this rare patient group.
4. Depending on their residual renal function, intensify the hemodialysis schedule of pregnant patients to at least 20 hours/week so that the maternal urea level will always be lower than 17.5 mmol/l from the second trimester onwards.
5. Consider treating hemodialysis patients without residual renal function during pregnancy with high-intensity hemodialysis, preferably with a dialysis intensity of >36 hours/week with the help of, for example, frequent night dialysis.
6. Do not use Kt/V measurements to assess the efficiency of high-intensity hemodialysis of pregnant patients.
7. Consider starting intensive hemodialysis for pregnant women with severe kidney failure who are not on dialysis yet, if despite an adequate low-protein diet (if necessary, supplemented with amino acids) the maternal urea level cannot be maintained below 17.5 mmol/l.
8. Regularly conduct fetal ultrasound (growth and doppler) throughout the pregnancy given the high fetal morbidity and mortality.
9. Pregnant dialysis patients treated with intensive hemodialysis should have a daily protein intake of 1.5 to 1.8 g/kg ideal weight per day.
10. Double the dosages of water-soluble vitamins for pregnant patients treated with intensive hemodialysis and start with deliberately high doses of folic acid (5 mg/day) even before conception because these substances will be removed in higher quantities by high-intensity hemodialysis.
11. Have a dietician frequently evaluate the nutritional status of pregnant dialysis patients because prolonged treatment with high-intensity hemodialysis significantly affects the patient’s fluid balance and nutritional status.

#### Immunosuppressive Medication (no GRADE)

1. Refer patients with CKD with a desire to have children for preconception counseling in a university hospital. A medication review and, if necessary, changing (teratogenic) medication must be part of the counseling.
2. If necessary, modify immunosuppression depending on the type of agent at least 3 to 6 months before any conception attempt to evaluate their effectiveness and minimize the risk of teratogenicity (Table 2).

**Table 2:** Summary of immunosuppressive medication used in chronic kidney disease and its periconceptional advise, teratogenicity, pregnancy advice, lactational advice, and paternal advice

Medication	Periconceptional advice	Placenta transfer	Known teratogenicity	Possible effect on neonate	Pregnancy advice	Transition into breast milk	Lactation advice	Paternal advice
Immunosuppressive medication								
Corticosteroids	Can be used	Yes, strongly dependent on type	In high doses in animals associated with schizis, but in human not unequivocally proven	With chronic use of prednisone dose >10 mg association with fetal growth restriction and neonatal adrenal suppression	Can be used	Yes, in small amounts	Can be used	Can be used
Hydroxychloroquine	Can be used	Yes	No	-	Can be used	Yes, in small amounts	Can be used	Can be used
Azathioprine	Can be used	Yes	No	Association of maternal leukopenia with neonatal leukopenia and pancytopenia	Can be used; if leukopenia: decrease dose in 3rd trimester to prevent neonatal problems after birth	Yes, in small amounts	Can be used	Can be used
Tacrolimus	Can be used	Yes	No	Association of maternal hyperkalemia with neonatal hyperkalemia and mild renal dysfunction	Can be used; increase dose from 1st trimester on basis of trough levels	Yes	Can be used	Can be used
IVIG	Can be used	Yes	No	-	Can be used	Yes	Can be used	Can be used
Ciclosporine	Can be used	Yes	No	Association with neonatal leukopenia	Can be used; increase dose from 1st trimester on basis of trough levels	Yes, in small amounts	Can be used	Can be used
Mycophenolate mofetil	Contraindication; stop at least 3 months before conception and switch to safer alternative	Yes	Associated with congenital defects in face, extremities, heart, esophagus and kidneys	-	Absolutely contraindicated	Unknown	Discouraged	Can be used
Cyclofosfamide	Discouraged	Yes	Association with congenital defects	-	Discouraged, use only with strict indication in 2nd or 3rd trimester (life threatening situations)	Yes	Discouraged	Discouraged
Methotrexate	Contraindication; stop at least 3 months before conception	Yes	Association with fetal mortality and congenital defects in face, extremities and central nervous system	-	Absolutely contraindicated	Yes, in small amount	Contraindicated	Can be used in low dose
Biologicals								
Alemtuzumab	Unknown risk	Unknown	Unknown	Unknown	Unknown risk	Unknown	Unknown risk	Unknown risk
Belatacept	Unknown risk	Unknown	Unknown	Unknown	Unknown risk	Unknown	Unknown risk	Unknown risk
Belimumab	Unknown risk	Unknown	Unknown	Unknown	Unknown risk	Unknown	Unknown risk	Unknown risk
Eculizumab	Can be used	Yes, in small amounts	No	Association with inhibition of terminal complement in fetal circulation	Can be used	Unknown	Can be used	Unknown risk
rATG	Unknown risk	Yes	Unknown	Unknown	Unknown risk	Unknown	Unknown risk	Unknown risk
Rituximab	Discouraged, stop at least 6 months before conception; use only when no safer alternative available	Yes	No	Risk for reversible neonatal B cell depletion when used in 2^nd^ or 3^rd^ trimester	Discouraged, use only when no safer alternative available	Unknown, problably not	Can be used, probably inactivation of active metabolites in gastrointestinal tract of neonate	Unknown risk

IVIG, intravenous immunoglobulins; rATG, rabbit anithymocyte globulin.
3. Only consider prescribing biopharmaceuticals before conception and during pregnancy if no other safe and effective treatment of a maternal disease is available and only in a center experienced in the treatment of pregnant patients with CKD using immunosuppression.

#### Anticoagulant Therapy (no GRADE)

1. Type of anticoagulant (low molecular weight heparins [LMWH] or vitamin K antagonists [VKA]), prophylactic or therapeutic dose, and duration of treatment strongly depend on the underlying disorder, concomitant comorbidity, and estimated thrombosis and bleeding risks.
2. During pregnancy, VKA should, in principle, be replaced by therapeutic LMWH due to VKAs teratogenic effects and increased fetal bleeding tendency during labor. An exception to this rule is pregnant women with a strict indication for VKA such as patients with certain types of low-flow mechanical heart valves.
3. Preconceptionally and, at the latest, after the first positive pregnancy test, patients with CKD treated with a direct oral anticoagulant should be switched to therapeutic LMWH due to the increased risk of miscarriage or congenital defects.
4. For pregnant patients with CKD with an indication for anticoagulation, continue the LMWH or VKA even after birth for at least 6 weeks due to the increased thrombosis risk in this early postpartum period.
5. Treat pregnant women with severe nephrotic syndrome and serum albumin <25 g/l with LMWH in prophylactic or, if necessary, therapeutic doses based on any additional thrombosis risk factors.
6. Consider giving LMWH in prophylactic doses to pregnant patients with CKD with elevated thrombosis risk due to the CKD (especially membranous nephropathy) without severe nephrotic syndrome because the pregnancy should be regarded as an additional thrombosis risk factor.
7. Treat pregnant women with systemic lupus erythematosus nephritis and/or antiphospholipid syndrome with low doses of acetylsalicylic acid to reduce the risk of arterial thrombosis and preeclampsia. Combine this drug with prophylactic doses of LMWH if the patient has obstetrical antiphospholipid syndrome.
8. If LMWH is given in therapeutic doses to pregnant patients with a higher-stage CKD (G3b-G5ND), consider monitoring their anti-Xa levels to prevent accumulation.

#### Diabetes Mellitus (No GRADE)

1. Consider the potentially increased insulin sensitivity and extended duration of action of insulin, particularly in patients with higher CKD stages.
2. Consider prescribing shorter-acting insulin types (e.g., insulin isophane) to pregnant patients with diabetes mellitus and CKD stage 3b or higher given the extended duration of action of insulin in these patients.
3. Consider more frequent blood sugar checks with a glucose sensor for pregnant patients with diabetes mellitus and CKD stage 3b or higher.
4. Prescribe corticosteroids to pregnant patients with diabetes mellitus (whether they have CKD or not) to promote fetal lung maturation only in clinical setting due to the need for intensive blood sugar monitoring and frequent insulin adjustments.

#### Follow-up of Preeclampsia or New Onset Kidney Disease After Delivery (no GRADE)

1. Patients with the following postpartum characteristics must be monitored at the nephrology clinic:
  • patients with acute renal failure superimposed on preeclampsia or hemolysis, elevated liver enzymes and low platelets syndrome whose renal failure does not resolve quickly and completely after the birth
  • patients who develop nephrotic syndrome, glomerulonephritis, thrombotic microangiopathy, or any other primary nephrological disorder during pregnancy.
2. Recommend that these patients be given nephrological and obstetrical preconception counseling before a future pregnancy.
3. Inform patients who have had preeclampsia about their increased lifetime cardiovascular risk and the need for regular follow-ups and, if necessary, treatment of other cardiovascular risk factors.
4. Advise patients who have only had preeclampsia to have their GP conduct life-long follow-ups due to their increased lifetime cardiovascular risk.

### Obstetric Treatment During Pregnancy

#### Advanced Ultrasound Investigation (no GRADE)

1. For any prospective parent (male/female) with CKD, consider whether there is an indication for advanced ultrasonography of the fetus.
2. All prospective parents with CKD due to a structural defect not based on a known genome abnormality and women with CKD who must use teratogenic medication during pregnancy have an indication for advanced ultrasonography of the fetus.

#### Decreasing Preeclampsia Risk (no GRADE)

1. Prescribe acetylsalicylic acid to any pregnant woman with CKD because they have a (substantially) higher preeclampsia risk.
2. Start prophylactic acetylsalicylic acid in doses of 80 to 150 mg/day from 12 weeks (after last menstruation) and preferably before the end of the sixteenth week. Stop the treatment at least 1 week before the expected natural birth or planned cesarean section, that is, usually at 36 weeks pregnancy, unless the birth is expected earlier.
  Recommend that the woman takes the acetylsalicylic acid in the evening.

3. Recommend that any pregnant woman with CKD use at least 1000 mg/day of elemental calcium (preferably in their food) because this may lower the preeclampsia risk.
4. If insufficient calcium is ingested with food, start elemental calcium supplementation of 500-1000 mg (preferably a combination of calcium and 400 to 800 IU of colecalciferol), to be taken in 1 dose on an empty stomach, with the dose depending on the dietary calcium intake.

Patients should not take calcium supplements at the same time as iron supplements, which should also be taken on an empty stomach.

Be cautious when prescribing calcium supplements to patients with advanced CKD stages (G4 and higher) and hyperphosphatemia despite adequate dietary measures.

5. Do not recommend sodium or protein restriction to prevent preeclampsia in pregnant women with CKD. If necessary, recommend this as part of their CKD treatment.

#### Delivery Plan (no GRADE)

1. In principle, aim at a vaginal delivery in women with CKD and/or a functioning kidney transplant. Time the delivery based on the clinical parameters. Discuss the advantages and disadvantages of inducing labor in patients with CKD after the thirty-eighth week due to the increased risks of preeclampsia, loss of renal function, and stillbirth.
2. In the event of obstetrical complications such as fetal growth restriction, preeclampsia, or deterioration of the mother’s condition and/or kidney function, consider inducing labor early, preferably in consultation with the nephrologist.
3. In patients with CKD stages G4 and above (eGFR < 30 ml/min per 1.73 m^2^), consider 1 or more of the following measures before or during labor:
  • try to avoid neuroaxial techniques in patients who have stopped taking acetylsalicylic acid less than 5 days before
  • if potentially severe bleeding is expected or occurs due to uremic thrombocytopathy, especially if the patient stopped taking acetylsalicylic acid less than 5 days before:
    ○ blood transfusions if their Hb drops below 6.2 mmol/l
    ○ 0.3 to 0.4 mcg/kg of desmopressin intravenous. When desmopressin is given, limit the patient’s fluid intake to a maximum of 1500 ml all-in over the first 24 hours after the desmopressin administration to prevent overfilling and hyponatremia with the associated risk of epileptic seizures. Regularly check the patient’s blood pressure and serum sodium levels for the first 24 hours after administration
  • preferably prime the cervix with a Foley balloon catheter
  • if labor must be induced, do so with oxytocin because this does not require dose adjustments in patients with CKD
  • lower the maintenance dose of magnesium sulfate: normal 4 g loading dose in 10 to 30 minutes and 0.5 g/hour maintenance dose. Frequently check the patient for signs of intoxication and regularly monitor their blood magnesium levels
  • any drug given during labor should be checked to verify that it is allowed and whether the dose should be adjusted; do not hesitate to consult with a nephrologist and/or pharmacist.
4. Test a sample of umbilical cord blood for hematologic parameters if the mother has used immunosuppressants such as azathioprine or biopharmaceuticals during pregnancy. These agents may cause maternal leukopenia and/or thrombocytopenia and are known to cause neonatal bone marrow suppression.

## Conclusion

The Dutch practice guideline on “Pregnancy Wish and Pregnancy in Patients with CKD” provides patient-centered, thorough, structured, and where possible evidence-based standard of care recommendations for patients with CKD throughout the process of planning a pregnancy to delivery and the first postpartum period including lactation.

## Disclosure

MFCdJ, IWHvE and ATL report receiving speaker fees from Alexion. All the other authors declared no competing interests.

## Appendix

Other members of the Dutch Guideline Working Group on Pregnancy in CKD include the following: Olivier W. H. van der Heijden, Y. K. Onno Teng, Albertien M. van der Eerde, Renate Bosma, Heleen Grootjans, Rob van der Pas, Merlijn Wind, Karen Prantl and Kim van Ierssel.

## Supplementary Material

Supplementary File (PDF)

**Dutch Practice Guideline: Pregnancy wish and Pregnancy in patients with chronic kidney disease**

**Figure S1.** Birth weight and NICU admittance according to prepregnancy CKD stage.

**Figure S2.** Maternal complications and admittance during pregnancy according to CKD stage.

**Figure S3.** Kidney outcome 6 months postpartum according to prepregnancy CKD stage.

**Table S1.** Considerations for use of antihypertensive agents during lactation.
