# Supplementary material for: Summary of the Dutch Practice Guideline on Pregnancy Wish and Pregnancy in CKD
Source: Kidney Int Rep. 2022 Oct 5;7(12):2575–88. doi: 10.1016/j.ekir.2022.09.029 (PMC9727525; doi:10.1016/j.ekir.2022.09.029)
Supplement: Supplementary File (PDF) [file mmc1.pdf]

Dutch practice guideline on Pregnancy Wish and Pregnancy in Chronic Kidney Disease

Margriet FC de Jong, MD PhD MBA[1], Henk W van Hamersvelt, MD PhD[2], Inge WH van Empel, MD PhD[3], Ellen JW Nijkamp MD[4], A. Titia Lely, MD PhD[4]

On behalf of the Dutch guideline working group Pregnancy in CKD[*]

[1] Department of Internal Medicine, Division of Nephrology, University Medical Center Groningen, Groningen, The Netherlands.

[2] Department of Nephrology, Radboud University Medical Center, Nijmegen, The Netherlands.

[3] Department of Obstetrics and Gynecology, Radboud University Medical Center, Nijmegen, The Netherlands.

[4] Department of Obstetrics and Gynecology, Utrecht University Medical Center, Utrecht, The Netherlands.

[*] Other members of the working group: Olivier WH van der Heijden, MD PhD, YK Onno Teng, MD PhD, Albertien M van der Eerde, MD PhD, Renate Bosma, MD PhD, Heleen Grootjans, MD, Rob van der Pas, MD PhD, Merlijn Wind, MD, Karen Prantl, Kim van Ierssel.

Corresponding author:

Margriet FC de Jong, MD PhD MBA, nephrologist

Department of Internal Medicine, division of Nephrology

University Medical Center Groningen (UMCG)

Hanzeplein 1

9713 GZ Groningen, The Netherlands

m.f.c.de.jong@umcg.nl

Introduction

Background

The number of women with chronic kidney disease (CKD) desiring a pregnancy is increasing. Firstly, because of increasing maternal age with inherent higher incidence of CKD and secondly, because of improved treatment options and higher numbers of kidney transplantations leading to increased number of fertile women with CKD for whom pregnancy can be a safe option. Furthermore, due to improved preconception care and multidisciplinary care before, during and after pregnancy, maternal and fetal outcomes have improved. However, still, preexisting CKD has negative effects on maternal and fetal outcomes (Figures 1 and 2). Patients with CKD or a kidney transplant have an increased risk for pregnancy complications such as temporary or permanent kidney function decline, hypertension, preeclampsia, preterm birth and intrauterine growth restriction.[1-9]

Aims

Aim of this guideline is to provide structured and where possible evidence-based standard of care for patients with CKD throughout the process of planning a pregnancy to delivery and the first postpartum period including lactation (Figure 3).

Scope

The clinical issues covered in this guideline are (Figure 3):

Part 1: Preconception risk stratification of pregnant women with CKD

- 1.1 Risk stratification
- 1.2 Preconception counseling
- 1.3 Preconception genetic counseling

Part 2: Nephrological treatment during pregnancy

- 2.1 Diet restrictions
- 2.2 Hypertension
  - 2.2.1 Target blood pressure before conception and during pregnancy with and without proteinuria
  - 2.2.2 Diuretics during pregnancy
  - 2.2.3 Safety of antihypertensive drugs used in pregnancy

2.2.4 Antihypertensive drugs and diet interventions in patients with proteinuria preconceptionally
2.2.5 Antihypertensive drugs during lactation
2.3 Anemia
2.3.1 Target values of iron parameters
2.3.2 Intravenous iron preparations
2.3.3 Treatment with rhEPO
2.3.4 Blood transfusion
2.4 New onset nephrological problems during pregnancy
2.4.1 Diagnostics of new nephrological problems (proteinuria /nephrotic syndrome/ thrombotic microangiopathy)
2.4.2 Differentiation between preeclampsia and kidney disease
2.4.3 Indication of kidney biopsy
2.5 Dialysis
2.6 Immunosuppressive medication
2.7 Anticoagulant therapy
2.8 Diabetes mellitus
2.9 Follow-up of preeclampsia or new onset kidney disease after delivery
Part 3: Obstetric treatment during pregnancy
3.1 Advanced ultrasound investigation
3.2 Decreasing preeclampsia risk
3.3 Delivery plan

Executive summary

The Dutch practice guideline on "Pregnancy Wish and Pregnancy in Patients with CKD" was developed by a multidisciplinary working group composed of all relevant specialisms and including representatives of patients, and published in the Dutch medical guideline database in 2021. Guideline recommendations are based on available relevant studies and appraisal of the quality of the evidence. The strength of recommendations is based on the "Grading of Recommendations Assessment, Development and Evaluation" (GRADE) approach. Scope of the guideline was determined by clinical issues, such as preconception (genetic) counseling, diet restrictions, treatment

of hypertension and anemia, immunosuppressive medication, and the obstetric treatment. The goal of the guideline is to provide clinicians and patients a useful resource with actionable patient-centered, structured and where possible evidence-based care recommendations for the process of planning a pregnancy to delivery and the first postpartum period including lactation. Finally, knowledge gaps for each clinical issue are described.

Lay summary

The Dutch practice guideline on “Pregnancy Wish and Pregnancy in Patients with CKD” was developed by a multidisciplinary working group composed of all relevant specialisms (nephrologists, gynecologists, clinical geneticist) and including representatives of patients. It was published in the Dutch medical guideline database in 2021. Guideline recommendations are based on available relevant studies and appraisal of the quality of the evidence. The strength of recommendations is based on the “Grading of Recommendations Assessment, Development and Evaluation” (GRADE) approach. Scope of the guideline was determined by clinical issues. Issues are for example:

-before an actual pregnancy discussing the pregnancy wish, with review of risk factors for pregnancy complications and worse kidney outcomes, need of referral to a clinical geneticist, and a medication review,

-diet restrictions,

-treatment of high blood pressure and anemia,

-immunosuppressive medication,

-the obstetric treatment.

The goal of the guideline is to provide clinicians and patients a useful resource with actionable patient-centered, structured and where possible evidence-based care recommendations. This includes the whole process of planning a pregnancy to delivery and the first postpartum period including lactation. Finally, knowledge gaps for each clinical issue are described.

Methods

For development of this guideline a multidisciplinary working group was composed with all relevant specialisms and including representatives of patients. Scope of the guideline was determined by clinical issues. Clinical issues were framed by a research question and background. Literature searches were performed by literature specialists and undertaken using specific search terms related to each of the issues covered. The research question, background, search and select method, results, conclusion, rationale and recommendations were described for each clinical issue. Strength of evidence was determined by GRADE-method (Grading Recommendations Assessment, Development and Evaluation).[10,11] When no GRADE was possible because studies found by literature search did not meet the inclusion criteria, studies were described in the analysis of the literature if relevant for the direction of the recommendations. For publication, recommendations were officially translated from Dutch by a forward and backward method. Other parts of the Dutch guideline were (somewhat shortened) translated by the authors.

Part 1: Preconception risk stratification and counseling

1.1 Risk stratification

Question

Where (general practitioner (GP), general hospital, academic hospital) should patients with CKD and pregnancy (wish) be counseled and treated based on the risks for maternal and fetal complications?

Background

CKD is a heterogenic patient group because of the diversity of kidney diseases and their diversity of clinical presentations. Counseling as well as treatment during pregnancy should be performed by an obstetrician with affinity for maternal disease. In advanced CKD stages these should be performed by a maternal-fetal medicine specialist and nephrologist with experience and expertise on pregnancy in CKD.

Search and select

A systematic review of the literature was performed to answer the following question:
Which factors are related to complications of pregnancy in mother and/or child in different stages of CKD? How do these factors contribute to the estimated risk of maternal and fetal/neonatal complications?

**P:** patient	women with CKD who are pregnant or wish to become pregnant;
**I:** intervention	presence of prognostic factors: degree of kidney insufficiency, degree of proteinuria, presence of hypertension;
**C:** control	absence of prognostic factors;
**O:** outcome measures	maternal complications of pregnancy: maternal mortality; cardiovascular incidents/cerebrovascular incidents; preeclampsia/hypertensive disorders of pregnancy/HELPP; loss of renal function/worsening kidney function fetal complications: small for gestational age (SGA)/intrauterine growth restriction (IUGR)/dysmaturity/birth weight; premature birth/prematurity.

Relevant outcome measures

The guideline development group considered maternal mortality, maternal cardiovascular events and fetal mortality and birth defects as a critical outcome measure for decision making; and maternal preeclampsia/hypertensive disorders of pregnancy/HELLP and fetal growth restriction and need for neonatal intensive care unit as an important outcome measure for decision making.
A priori, the working group did not define the outcome measures listed above but used the definitions used in the studies.

Search and select: methods

The databases Medline (via OVID) and Embase (via Embase.com)) were searched with relevant search terms from 1st of January 1998 until 22nd of February 2019. The systematic literature search resulted in 4821 hits. Studies were selected based on the following criteria: original studies or systematic reviews that described the relation between prognostic factors and maternal and neonatal outcomes in women with CKD who were pregnant or planning to get pregnant. The relation between these factors had to be corrected for confounders in a multivariate model. As a minimum the model had to be corrected for kidney function as a confounder.
Ninety-six studies were initially selected based on title and abstract screening. After reading the full text, all studies were excluded because none of them fulfilled all the inclusion criteria.

Fourteen studies were found that did not report a multivariate model describing the relation between prognostic factors and maternal and neonatal outcomes in women with CKD who were pregnant or planning to become pregnant. However, these studies did report this relation in a univariate analysis. Six of the 14 studies are described below, in order to present at least an overview of less direct evidence. However, no GRADE-assessment of these studies is performed, since the minimum criteria for inclusion are not fulfilled.
One retrospective cohort study describing maternal and fetal outcome in patients with CKD stage 3-5,[9] published after the closure of the search strategy (February 2019), was considered highly relevant for the guideline development and was included in the analysis.

<u>Results</u>
Fourteen studies did not meet the inclusion criteria but are described in the analysis of the literature, including one systematic review of the literature, because they are relevant for the direction of the recommendations.

Summary of literature
1. **Meta-analysis Zhang (2015)**[4]: the authors undertook a systematic review and meta-analysis of cohort and case control studies to estimate the risk of pregnancy complications among patients with CKD versus those without CKD, and the risk of disease progression among women with CKD. Twenty-three studies with 504,700 pregnancies were included. Women with CKD and pregnancy had far worse pregnancy outcomes than women without CKD: the overall odds ratio (OR) for preeclampsia was 10.36 (95% (confidence interval (CI), 6.28 to 7.09; p<0.001), pregnancy failure (described as still-birth and neonatal death) was 1.80 (95% CI, 1.03 to 3.13; p<0.04), prematurity (< 37 weeks gestation) was 5.72 (95% CI, 3.26 to 10.03; p<0.01), small for gestational age was 2.67 (95% CI, 2.01 to 3.57; p<0.001) and caesarean section was 4.85 (95% CI, 3.03 to 7.75; p<0.01). There were no significant differences in the occurrence of renal events (doubling of serum creatinine levels, 50% decrement of GFR, or end stage renal disease (ESRD)) during median follow-up of 5 years between CKD pregnant women with glomerular diseases (mainly IgA nephropathy) and those with similar diseases but without pregnancy (OR, 0.96; 95% CI, 0.69 to 1.35). However, in most of these 9 studies on renal events, patients with preserved renal function (CKD stages 1 and 2) were included.

2. **Piccoli (2012)**[1]: two hundred and forty-nine pregnancies were monitored in 225 CKD patients over an 11-year period in a tertiary referral center in Italy. After exclusion for several reasons, 179 singleton pregnancies in CKD patients (n=127 CKD stage 1) were compared with a historical control group of 267 singleton 'low-risk pregnancies' in the same center Maternal and fetal outcome were described. CKD stage 1 patients were compared with healthy

controls and with patients with CKD stages 2 to 4 using multivariate logistic regression analysis. As compared to healthy parous controls, pregnant patients with CKD stage 1 have a higher rate for preterm delivery < 37 weeks (OR 8.5; 95% CI 4.11 to 17.57), early preterm delivery < 34 weeks (OR 7.33; 95% CI 2.25 to 23.83) and need for neonatal intensive care unit (NICU) admission (OR 16.1; 95% CI 4.42 to 58.66). Furthermore, when compared to CKD stage 1, pregnancy in women with CKD stages 2 to 4 was associated with a greater OR for preterm delivery < 37 weeks (OR 2.84; 95% CI 1.30 to 6.20) and NICU admission (OR 2.59; 95% CI 1.10 to 6.06). Women with proteinuria > 1g had a greater risk of child admission to the NICU (OR 4.40; 95% CI 1.38 to 14.07). Clearly, the higher the CKD stage, the worse the fetal outcomes with increasing percentages of preterm delivery < 37 weeks from 28% in stage 1 to 100% in stage 4 (controls 5%), early preterm delivery < 34 weeks from 10% in stage 1 to 50% in stage 4 (controls 1.5%), small for gestational age < 10 percentile from 14% in stage 1 to 75% in stage 4 (controls 10%) and need for NICU from 14% in stage 1 to 100% in stage 4 (controls 1%). Of note, only 17 patients with stage 3 and 4 patients with stage 4 were included in this analysis. In all stages of CKD, an increase in proteinuria and small rise in creatinine was observed from the first control to the last control during pregnancy. The increase in proteinuria showed a very wide scatter in all stages varying from no increase at all to nephrotic range proteinuria. The small overall rise in creatinine from 61.6 to 64.3 umol/L should be interpreted with caution because in a substantial part of the patients, the first control of creatinine was performed during the first half of pregnancy were physiologic hyperfiltration will have lowered creatinine.

3. **Piccoli (2015)**[2]: in the Italian Torino-Cagliari observational TOCOS study, maternal and fetal outcome of 504 singleton pregnancies in women with CKD stages 1 to 5 were compared to those of 836 healthy parous controls. The risk for adverse perinatal outcome increased across CKD stages, where the combined outcome parameter preterm delivery < 37 weeks, SGA and NICU admissions rose form 34% in stage 1 to 90% in stage 4 to 5. Similarly, the combined parameters early preterm delivery < 34 weeks, SGA and NICU admissions rose from 21.4% in stage 1 to 80% in stage 4 to 5. In the largest cohort of women with CKD stage 1, preterm delivery was associated with baseline hypertension (OR 3.42; 95% CI 1.87 to 6.21), proteinuria (OR 3.69; 95% CI 1.63 to 8.36), and systemic disease consisting of diabetic nephropathy, SLE, collagen diseases or vasculitis, and kidney transplantation (OR 3.13; 95% CI 1.51 to 6.5). Moreover, even in the absence of baseline hypertension, proteinuria, or systemic disease, CKD stage 1 was still associated with the combined adverse perinatal outcome parameter of preterm delivery, NICU, and SGA (OR 1.88; 95% CI 1.27 to 2.79).

4. **Piccoli (2017)**[6]: This study aimed to investigate the effect of common types of glomerulonephritis on the pregnancy outcome in 126 women (CKD stage 1 to 5; 37 lupus glomerulonephritis and 33 IgA nephropathy) and compared these to 1418 low-risk parous controls without CKD from the TOCOS cohort. Using multiple regression analysis, preterm delivery < 37 weeks and early preterm delivery < 34 weeks rose significantly across CKD stages 1 to 5 (resp. from 33.3% in stage 1 to 82.4% in stages 3 to 5, p<0.001; and early preterm delivery from 9% to 35%, p<0.009). The risk for the development of new onset hypertension, new onset proteinuria and doubling of proteinuria also increased considerably across CKD stages.
5. **He (2018)**[7]: nearly 300 pregnancies in women with CKD were studied retrospectively in a Chinese setting. The reproductive outcomes in stages 3-4 (n=30) in particular were described and compared to those of stages 1 (n=197) and 2 (n=73) and follow-up of stage 3-4 CKD was partly performed by telephone interview. Preterm birth was markedly increased in stages 3 to 4 as compared to stages 1 and 2 (57% versus 18% respectively, p<0.001), as was low birth weight (44% versus 15%, p<0.001). Renal function itself did not deteriorate throughout pregnancy *per se*, but the chance of progression to ESRD after pregnancy was higher in stage 3-4 CKD patients. However, in multivariate analysis, the chance of developing ESRD in pregnant stage 3-4 CKD patients was comparable to the chance of ESRD in a control group of non-pregnant stage 3-4 CKD patients. In this multivariate analysis, only baseline serum creatinine and proteinuria were risk factors for renal function deterioration in patients with stage 3–4 CKD, while pregnancy itself did not increase the risk of renal function deterioration.
6. **Hladunewich (2014)**[3]: pregnancy outcomes in women with end stage renal disease (ESRD) receiving hemodialysis were monitored in a comparison cohort study between Canadian (PreKid Clinic Registry) and United States ((American Registry for Pregnancy in Dialysis Patients) women. The 2 cohorts were comparable with respect to the causes of ESRD. Intensive hemodialysis throughout pregnancy resulted in a significant percentage live birth (86% in the Canadian cohort and 61% in the US cohort). In addition, only 6% of Canadian children born were classified as very low birth weight (versus 29% in the US cohort).
7. **Wiles (2021)**[9]: the impact of CKD stages 3-5 on pregnancy outcomes was studied in 178 pregnancies beyond 20 weeks of gestation and included 43 women with renal transplants. The reproductive outcomes of these 159 women in 6 UK tertiary renal centers between 2003 and 2017 were studied and data were collected in a retrospective cohort analysis. Chronic hypertension was the strongest predictor of preterm delivery < 34 weeks gestation (32%), and this risk was higher (increasing from 20% to 40%) if the gestational fall in serum

creatinine was < 10% of pre-pregnancy values. Pre-pregnancy proteinuria > 1 g/10 mmol creatinine increased the risk of birthweight below the 10th percentile with odds ratio of 2.57. Remarkably, there was a large drop in eGFR between pre-pregnancy and post-partum values, and this drop (4.5 mL/min/1.73m^2) was much higher than the annual decline in eGFR prior to pregnancy (1.8 mL/min/1.73m^2), resulting in considerable reduction of renal survival of 1.7, 2.1 and 4.9 years in CKD stages 3a, 3b and 4–5, respectively. Th pregnancy associated decline in renal function was greater in women with chronic hypertension and in those with a gestational fall in serum creatinine of < 10% of pre-pregnancy concentrations. As a consequence, the decline in renal function at 1 year postpartum was significant: almost half of all women had lost more than 25% of their pre-pregnancy eGFR or required renal replacement therapy.

Conclusion

**- GRADE**	No studies were found describing the relation between properties of CKD (degree of kidney insufficiency, degree of proteinuria, presence of hypertension) and maternal and fetal outcomes in women with CKD who are pregnant or wish to become pregnant taking in account correction for confounders.
**- GRADE**	There is a body of literature that links adverse maternal and/or fetal outcome to women to all stages of CKD.<br>Preeclampsia, preterm birth, and impaired fetal growth are by far the most prevalent negative reproductive outcomes in these women.<br>*Sources: (He, 2018; Hladunewich, 2014; Piccoli, 2015; Piccoli, 2016; Piccoli, 2012; Wiles, 2021; Zhang, 2015)*

Rationale

Preexisting CKD has negative effects on maternal and fetal outcome and these are more pronounced in more advanced CKD stages (Figures 1 (adapted from Zhang et al.[4]) and 2 (adapted from Piccoli et al.[1,2,6]), and Figures S1 (adapted from Piccoli et al.[1,2,6] and local unpublished data), S2 (adapted from Piccoli et al.[1,2,6], Wiles et al.[9] and local unpublished data), and S3 (adapted from Bramham et al.[5])). There is an increased risk for hypertension, preeclampsia, abruptio placentae, decrease in maternal kidney function, intrauterine fetal growth restriction and (partly iatrogenic) preterm birth.[1,2,4,5,6,9] These complications often lead to hospital admission, and length of stay increases with higher CKD stages. Decrease in kidney function was more pronounced in more advanced stages CKD: loss of year

equivalents of preconceptional kidney function decline was 1.7, 1.8 and 4.9 ml/min/1.72m2 for CKD stage 3a, 3b and 4-5 respectively.[9]

Patients with systemic autoimmune disease, diabetic nephropathy and kidney transplantation have an increased risk of adverse maternal and fetal outcome, with an increased risk of doubling of proteinuria, a 3 times increased risk of preterm birth and 6 times higher risk of NICU admission.[2]

Patients with systemic lupus erythematodes (SLE) nephritis, have increased risk for obstetric and maternal complications, especially with active disease. Even with disease in remission, spontaneous abortion, preterm birth, intrauterine growth restriction and perinatal mortality are prevalent.[12] All pregnancies in patients with SLE nephritis should be regarded as high-risk pregnancies.

Also, pregnancy in kidney transplant recipients (KTR) has significant risks for mother and child. Complications such as acute rejection, hypertension, anemia, urinary tract infections, and decline of kidney function because of calcineurin inhibitor toxicity, chronic graft failure and recurrent kidney disease. The fetus may suffer from (severe) fetal growth restriction and prematurity. Pregnancy care should be multidisciplinary by a nephrologist, maternal-fetal medicine specialist (both with experience and expertise in the field of pregnancy in kidney transplantation), and pediatrician-neonatologist. Pregnancy outcomes in KTR are favorable in patients with good and stable kidney function and in absence of severe increased albuminuria (proteinuria >1g/day).[8]

<u>Recommendations</u> (no GRADE)

1. Preconception consultation of women with CKD stage 3 or higher, or with a kidney transplant, or with a systemic autoimmune disease (regardless of the use of immunosuppressants) should preferably be performed in a university hospital by a maternal-fetal medicine specialist and a nephrologist. Both should be experienced in supervising pregnant women with CKD.
    - For women with CKD stage 1 or 2 without a kidney transplant or systemic autoimmune disease, the working group suggests that it is useful to receive counselling at a university hospital.

2. Pregnant women with CKD stage 1 or 2 without a kidney transplant and without systemic autoimmune disease needing immunosuppressants may receive specialist care in a general

hospital. This care should be given by an obstetrician and a nephrologist with affinity for pregnancy in CKD patients.

If the patient's condition or renal function deteriorates or fetal complications (are likely to) develop, the attending physician should consult with the specialized team at a university hospital and refer the patient without hesitation.

3. Pregnant women with CKD stage 3 or higher should preferably be treated in a university hospital by a maternal-fetal medicine specialist and a nephrologist who should both be experienced in the treatment of pregnant CKD patients.

4. Referral to a university hospital is strongly recommended for patients with a kidney transplant, patients with CKD due to a systemic autoimmune disease (needing immunosuppressants) and dialysis patients.

Knowledge gaps

- Which factors predict the risk for maternal and fetal complications in pregnant women with CKD?
- Does selection by risk factors of pregnant women with CKD for treatment in the regional hospital or an academic hospital lead to better maternal and fetal outcomes?

1.2 Preconception counseling

Questions

1. How should preconception counseling be performed?
2. When should be started with preconception counseling?

Background

The prevalence of CKD increases worldwide. Incidence of women with CKD planning a pregnancy will also increase. The five stages of CKD are determined by eGFR and range of albuminuria.[13] Approximately 3% of fertile women (20-39 years) have CKD stage 1 or 2. Approximately 1:150 fertile women suffer from CKD stage 3-5, but because of decreased fertility and spontaneous abortions the incidence of pregnancy will be lower. It is estimated that 1 in 750 pregnancies is complicated by CKD stage 3-5.[14]

Preexistent CKD has effect on maternal and fetal outcomes. It is important to inform patients with CKD who are planning a pregnancy, because of increased risk for maternal and fetal complications, the risk for congenital malformations due to use of teratogenic medication, and genetic causes of kidney disease.

Search and select, Results, Conclusion

Not applicable. No systematic literature search was performed, because the Dutch working group found the question was not suitable for a systematic literature search.

Rationale

Anticonception

When the woman with CKD has no active pregnancy wish, safe and effective anticonception should be discussed. Unplanned pregnancies have probably worse pregnancy outcomes than planned pregnancies, especially with the use of teratogenic medication or progressive kidney disease.

Most of the patients with CKD can use all types of anticonception, with preference for most reliable methods such as oral anticonception with the combination of estrogens and progestogens or a hormonal intrauterine device (IUD). Because of the slightly increased risk for thrombosis in patients with CKD, estrogen dose should be 30 mcg ethinylestradiol. Lower doses have an increased risk of failure or irregular bleedings. Despite renal clearance, dose adjustment for kidney function is not necessary.

In patients with CKD and extra increased thrombosis risk such as previous thrombosis, atypical hemolytic uremic syndrome (aHUS), SLE or antiphospholipid syndrome, other methods are preferred.

Hormonal IUD and hormonal implants have better reliability than oral anticonception with progestogens only.
Patients can be referred to a gynecologist for personal advice. The patient should be instructed to discuss a future pregnancy wish with her nephrologist.

Timing and location of multidisciplinary counseling
A pregnancy wish develops under social and relational factors, but in patients with CKD, optimal timing is also determined by course of kidney disease and necessary treatments. The working group therefore advices to discuss a pregnancy wish with all (male and female) patients in fertile age (18-45 years).
When a pregnancy wish exists, the patient should be referred for multidisciplinary counseling, so that there is enough time to discuss all relevant topics. Sometimes it appears necessary to reconsider cause of kidney disease. Furthermore, teratogenic medication must be converted to suitable alternatives, and preferably effects of these alternatives are monitored and evaluated. Therefore, strive for preconception counseling 6-12 months before possible conception.
The preconception counseling is preferably performed multidisciplinary with at least a nephrologist and maternal-fetal medicine specialist, both with experience with pregnancy in patients with CKD. The working group recommends that it must be performed in a university medical center with expertise in CKD and high-risk pregnancies when the patient has a complex underlying disease and/or CKD stage 3-5 and/or a kidney transplantation.

Content of preconception advice
General preconception advices need to be discussed, as are the specific topics below.

1. Influence of pregnancy on course of kidney disease. If possible, effect on the underlying kidney disease should be discussed. CKD stage needs to be taken into account to discuss risk for decrease in kidney function.
2. Influence of kidney disease on pregnancy. Fertility can be decreased because of underlying disease or CKD stage. Menstrual cycle should be discussed. Risks for pregnancy complications including hypertension, preeclampsia (PE), intrauterine growth restriction and (iatrogenic) preterm birth should be discussed. Strive for stable disease. Discuss blood pressure and use of acetylsalicylic acid to prevent PE.
3. Risks of medication for the fetus and risks for the mother when medication needs to be converted. Discuss a time line to evaluate new medication and dose-adjust for kidney function.

4. Possible genetic cause of kidney disease. Evaluate if the patient or her spouse may have a genetic kidney disease. Refer to a clinical geneticist with a low threshold. In particular with monogenetic hereditary kidney disease preimplantation diagnostic should be discussed.[15]
5. Give individual advice. Be careful for too much focus on the pregnancy risks and try to address the risks positively.[16] Strive for an informed choice for the pregnancy and the timing in relation to stable disease.

General topics that need to be discussed are quitting smoking and use of alcohol/drugs (patient and spouse) and weight reduction when BMI>25 kg/m^2. Discuss the importance of use of folic acid 1dd400 mcg preconceptionally.
Discuss to whom and when the patient should call when she's pregnant, confirmed by a positive pregnancy test.

Recommendations (no GRADE)

1. Recommend CKD patients who do not want to become pregnant (yet) to use safe and effective contraception. Actively inquire into the desire to conceive.

2. Consultation with a CKD patient who wants to conceive – to be conducted by a nephrologist and a maternal-fetal medicine specialist – should at least address the following aspects:

- the possible effects of pregnancy on the underlying kidney disease and assessment of the risk of transient and/or permanently loss of renal function;
- timing of pregnancy;
- the effects of CKD on pregnancy, specifically the risks of pregnancy complications such as hypertension, preeclampsia, intrauterine growth restriction, and (iatrogenic) premature birth (Figures 1 and 2);
- if the woman becomes pregnant, her life expectancy and quality of life (especially in case of end-stage renal failure), so that the woman and her partner can make an informed choice

about how to fulfil their desire to have children. Those close to the patient and her partner can also be involved in this process;

- the risk of (potentially) teratogenic medication and, if possible, recommend discontinuation or switch to a safe alternative;
- periodical evaluations of the likelihood that patients (m/f) with a latent desire to have children may have hereditary kidney disease; do not hesitate to refer them to a clinical geneticist;
- if the kidney disease is hereditary and carries an elevated risk of inheritance for future children, inquire if the couple would like to be informed about the various options for fulfilling their desire to have healthy children without the genetic disorder;
- general preconception advice including stopping smoking, alcohol and/or drugs, weight reduction if BMI > 25 kg/m^2, and prophylactic use of daily 400 mcg of folic acid before conception.

3. The disease of CKD patients with a desire to conceive should be stable; if necessary, regulate their blood pressure (< 130/80 mmHg).

4. Formulate a multidisciplinary treatment plan depending on the patient's condition and an assessment of the risk of complications.

5. Also recommend that the patient takes acetylsalicylic acid from the 12th week of pregnancy (after last menstruation) to reduce the risk of hypertensive disorders during pregnancy (see also recommendation 3.2.2).

<u>Knowledge gaps</u>

Which aspects of preconception counseling should be different in patients with CKD in comparison to patients without CKD?

1.3 Preconception genetic counseling

Question

Which patients need to get preconception genetic counseling? When should preimplantation genetic diagnostics be used?

Background

Approximately 15% of patients with CKD have a hereditary disease. When CKD presents <20y of age, the risk for a hereditary disease is even 20-30%.[17] The last decades more insights in molecular pathogenesis of several kidney diseases have been gained because of technical improvements such as Next Generation Sequencing and single nucleotide polymorphism (SNP) array.[18] Genetic diagnostics can lead to finding the cause of kidney disease but also to adjustment of existing clinical diagnosis. Making the genetic diagnosis can have important consequences. Firstly, the diagnosis may guide follow-up and treatment of renal and (asymptomatic) extrarenal manifestations.[17] Secondly, a genetic cause may guide informing family members. Thirdly, a genetic diagnosis gives information about repeat risk for the child.

Search and select, Results, Conclusion

Not applicable. No systematic literature search was performed, because the Dutch working group found the question was not suitable for a systematic literature search.

Rationale

Preconception genetic counseling

When a specific genetic primary kidney disease is found, the preconception genetic counseling can be individualized:

1. Spectrum of clinical phenotype and risk of inheritance.
2. Is it possible or wanted to perform screening during pregnancy?
3. Is there an indication/possibility for preimplantation genetic diagnostics?
4. Can perinatal aspects be anticipated?
5. Is there an indication for postnatal follow-up or screening, and from which age?

Refer to a clinical geneticist for counseling with a low threshold, in particular with patients without diagnosis, with doubt for the existing diagnosis, with symptomatic patients <20y or severe disease <40y, and with positive family history. Consider referral for patients with determined genetic disease,

in particular when the adult patient has not been referred yet, or has been with the clinical geneticist >5y ago.

All reproductive options are discussed:

1. Accepting the risk of inheritance in the child.
2. Preimplantation genetic diagnostics
3. Germ cell donation
4. Prenatal diagnostics
5. Adoption / foster care
6. Deny child wish.

Consider referral to specialized social work.

Invasive Prenatal diagnostics (PND)

By placental biopsy (11-13 weeks of gestational age) or amniocentesis (16 weeks of gestational age) is DNA of the placenta or fetus investigated. Risk for abortion is 0.3% with amniocentesis and therefore only done when the parents consider terminating the pregnancy when the fetus has the genetic disease.

Preimplantation genetic diagnostics (PGD)

An IVF or ICSI procedure is performed and an embryo without the genetic disease is selected before placement in the uterus. Referral for PGD-care is always performed by the clinical geneticist.

Advanced ultrasound investigation

When there is a risk for congenital birth defects of the kidneys or urinary tract (e.g. when the mother of father has a monokidney or reflux nephropathy), there is an indication for advanced ultrasound investigation with focus for the kidneys and urinary tract with a gestational age of 18-20 weeks.

<u>Recommendations</u> (no GRADE)

1. At an early stage, assess whether CKD patients (m/f) with a potential or actual desire to have children may have hereditary kidney disease.

2. Do not hesitate to refer CKD patients (m/f) with a potential desire to have children to a clinical geneticist for counselling and, if necessary, additional testing. At least consider referral if:

- patients are undiagnosed or if there is doubt about the current diagnosis; this includes:
    - patients with a severe manifestation of their CKD, for example:
        - symptomatic CKD at a young age (< 20y),
        - CKD stage 4 or 5 in a patient under 40y;
- patients with a positive family history for primary kidney disease or hereditary disorders with renal involvement such as atypical hemolytic uremic syndrome;
- adult patients with a confirmed hereditary disorder if they have not had preconception counselling or if this was more than 5 years ago;
- patients who desire prenatal diagnostics (PND):
    - who consulted with a clinical geneticist more than two years ago,
    - whose PND report/instructions are not explicit enough,
    - who did not receive genetic counselling at the centre where PND will be conducted.

  In the cases above, contact the clinical geneticist at the centre where PND will be conducted to verify if a referral is indicated.
- Patients with a confirmed hereditary disorder who may wish to be eligible for pre-implantation genetic diagnostics (PGD; in The Netherlands patients will always have to be referred for such PGD via a clinical geneticist).

Part 2: Nephrological treatment during pregnancy

2.1 Diet restrictions

Questions

What is the role of diet restrictions in pregnant women with CKD?

1. What is the effect of diet interventions (namely salt and protein restriction) in healthy pregnant women on maternal and fetal pregnancy outcomes?
2. What is the effect of diet interventions (namely salt and protein restriction, but also potassium and phosphate restriction) in pregnant women with CKD stage 3b-5 and/or proteinuria >1 gram/day on maternal and fetal pregnancy outcomes?

Background

Patients with CKD stage 3b and higher often get diet restrictions to improve for instance blood pressure, proteinuria and phosphate homeostasis. It is not clear whether these diet restrictions can also safely be practiced during pregnancy, thus without negative effects for patient as well as fetus. In general, a 'healthy diet' is associated with a lower incidence of CKD and albuminuria.[20]

Search and select

A systematic literature search was performed for the following search question:

What are the positive and negative effects of diet restrictions during pregnancy in women with CKD?

P: patients	healthy pregnant women and pregnant women with CKD stage 3b-5 and/or proteinuria > 0.5 gram/day (stage A3)
I: intervention	diet interventions including restrictions of salt, protein, potassium and phosphate
C: control	no diet interventions
O: outcome	pregnancy outcomes in patients with CKD and/or proteinuria: incidence of maternal complications (severe hypertension, need for dialysis, symptomatic fluid overload) and fetal complications (including polyhydramnios)

Relevant outcome measures

The working group found the following outcomes crucial: maternal: severe proteinuria, need for dialysis and acute kidney injury, and fetal: birth weight, NICU admission and polyhydramnios. The

working group found the following outcomes important: maternal symptomatic fluid overload and severe hypertension.

Search and select

The search was performed on 16 March 2019 in the databases Medline (OVID), Embase (embase.com) and Cochrane Library (Wiley) from 1960. Relevant search terms were used to search for (randomized and observational) studies which compared a diet intervention with another diet intervention or standard care in pregnant women with CKD. N=556 studies were found. Studies were selected by the following criteria: original investigation or systematic review of original investigations; (randomized and observational) studies which compared a diet intervention with another diet intervention or standard care in pregnant women with CKD. No studies were found which investigated diet interventions in pregnant women with CKD. Therefore, also studies which investigated diet interventions in pregnant women were included, but these studies were downgraded with one level for directness (other patient population). N=71 studies were preselected by title and abstract. After full text reading, n=69 studies were excluded and n=2 studies included.

<u>Results</u>

Description of studies

We searched for randomized controlled trials or systematic reviews that studied the effects of dietary interventions on pregnancy outcomes in both healthy pregnant women and pregnant patients with chronic kidney disease. Unfortunately, no such studies were found that included patients with chronic kidney disease. Two systematic Cochrane reviews were found that reported the effects of dietary restrictions in healthy pregnant women.

Duley[20] included two studies in a Cochrane review in which 603 healthy pregnant women were randomized to either a low dietary salt intake (goal: 20 and 50 mmol/day, respectively) or normal dietary salt intake in the second and third trimester. Although the intended salt intake was not reached in the intervention arms, these patients reached an average daily sodium excretion of 70 and 84 mmol/day, respectively, which is considered a firm salt restriction. Most studied outcomes had wide confidence intervals and no statistically significant effects were reported on either maternal (hypertension, preeclampsia, hospital referral/admission, placental abruption, caesarean section) or fetal (perinatal mortality, low birthweight, preterm delivery, Apgar score or pediatric admission) outcomes. Thus, salt restriction in second and third trimester of pregnancy seemed to be neither beneficial nor harmful in healthy pregnant women.

Kramer[21] systematically reviewed 24 studies that evaluated various dietary interventions in pregnancy. In 4 of these studies, the effects of protein/energy restriction were examined in

overweight women or women with high weight gain during pregnancy. The timing of inclusion in these studies varied from 15 to 30 weeks gestation. In 3 studies, patients in the intervention arm were prescribed a low energy diet (1200, 1250 and 1500 to 2000 kcal/day, respectively) while in the fourth study, patients received 10 dietary consultations of 1 hour each. Patients in the control arms did not have any dietary restrictions. Total protein intake in these patients was not reported and it is not clear, therefore, whether or not these women used a protein-restricted diet. As expected, weekly weight gain was lower in the intervention arms (230 grams on average), but no effects were found on preeclampsia, pregnancy-induced hypertension, pre-term birth, birthweight, gestational duration or gestational diabetes.

Level of evidence

The level of evidence was graded as very low for the following outcome measures:

- pregnancy outcome with respect to kidney function and/or proteinuria;
- incidence of maternal complications (severe hypertension, preeclampsia, mortality);
- fetal complications (amongst which polyhydramnios, prematurity, growth restriction).

For all the outcome measures systematic reviews were available. Thus, the GRADE level started with high, but was decreased with 2 levels for indirectness (patients in studies were healthy, as opposed to our patients of interest: with chronic kidney disease); and 1 level due to imprecision (small number of patients).

<u>Conclusion</u>

**Very low GRADE**	In pregnant women with chronic kidney disease, the effect of sodium restriction as opposed to no sodium restriction is not clear for the following outcomes:<br>• maternal pregnancy outcome with respect to kidney function and/or proteinuria;<br>• incidence of maternal complications (severe hypertension, preeclampsia, mortality);<br>• incidence of fetal complications (amongst which polyhydramnios, prematurity, growth restriction).<br>*Sources: (Duley, 2005)*
**Very low GRADE**	In pregnant women with chronic kidney disease, the effect of protein restriction as opposed to no protein restriction is not clear for the following outcomes:<br>• maternal pregnancy outcome with respect to kidney function and/or proteinuria;

	• incidence of maternal complications (severe hypertension, preeclampsia, mortality);<br>• incidence of fetal complications (amongst which polyhydramnios, prematurity, growth restriction).<br>*Sources: (Kramer, 2003)*

Rationale

No RCTs or systematic reviews on the effects of diet interventions in pregnant women with CKD were found. The included systematic reviews investigated the effects of sodium and protein/caloric restriction in healthy women. Besides these systematic reviews 8 studies were found that reported on consequences of diet interventions in healthy pregnant women or pregnant women with CKD.

Effect of sodium restriction on pregnancy outcome

Knuist et al.[22] investigated healthy Dutch pregnant women with a normal blood pressure before 20 weeks gestational age. Women were randomized for sodium restriction (≤50 mmol/day) or a normal diet when diastolic blood pressure increased (≥85 mmHg), body weight gained >1 kg/week during 3 consecutive weeks, or severe edema developed (not further specified). In both groups approximately 180 women were randomized: 27% before 28 weeks, 30% between 28-36 weeks and 43% after 36 weeks. Sodium restriction did not change diastolic blood pressure nor decreased the number of women referred to the hospital. In the intervention group sodium excretion decreased from 117 to 84 mmol/24h and 24% reached <50 mmol/24h. Obstetric outcomes did not differ between groups.
Franx et al.[23] prospectively studied the association between sodium intake and blood pressure in the second and third trimester of pregnancy in a low-risk population of 667 patients with normal blood pressure (defined as diastolic pressure <90 mmHg). No association was found between changes in sodium excretion and changes in systolic or diastolic blood pressure during pregnancy. There were also no differences in sodium excretion between women who remained normotensive and women that developed hypertension.
Inoue et al.[24] neither found an association between sodium excretion and home blood pressure in 184 pregnant Japanese women before 20 and after 30 weeks, not even in patients with pregnancy induced hypertension (which occurred in 7.6% in this population).
In a Dutch prospective study in healthy nulliparae the uteroplacental circulation was compared between pregnant women randomized for sodium restriction (target 20 mmol/day) or no diet restriction (both groups n=72) from 14 weeks gestational age until delivery.[25] Sodium restriction had no influence on physiological decrease of pulsatility index and resistance index of the uterine artery.

Moreover, no prognostic negative flow patterns were observed in the salt restricted women and no statistically significant differences in pregnancy outcomes were found.
In summary, there is no evidence that a sodium restriction of 3-6 grams salt = 1200-2400 mg sodium from 14-28 weeks of pregnancy is associated with positive or negative maternal or fetal outcomes in healthy pregnancies.
However, since sodium restriction has been found to have positive effects in patients with CKD in general[26] and no evidence of harmful effects for mother or fetus during pregnancy exists, the working group recommends for all pregnant patients with CKD a maximal salt intake of 6 gram/day = 2400 mg sodium/day. On indication the salt intake can safely be restricted to 3 gram/day = 1200 mg sodium/day, e.g. in pregnant patients with a severe nephrotic syndrome, difficult to treat hypertension and/or (expected) severe sodium retention in the higher CKD stages. For compliance reasons, the working group recommends to check sodium excretion regularly.

Effect of protein restriction on pregnancy outcomes
A large observational cohort study from Japan showed a U-shaped association between protein intake during pregnancy and birth weight.[27] More than 80,000 healthy women responded to a questionnaire about food intake early and halfway their pregnancy. Birth weight was highest in women with a diet in which 12% of total energy intake was from protein. The lower the percentage, the lower the birth weight was, but the birth weight was also lower in higher percentages energy intake from protein. Thus, extreme protein deprivation could have negative effects on pregnancy outcome.
No RCTs or systematic reviews on the effects of protein restriction in pregnant women with CKD were found. However, 3 Italian uncontrolled studies evaluated protein restriction during pregnancy in women with CKD.[28-30] These studies suggest that a vegan/vegetarian diet supplemented with amino acids are at least not harmful for pregnant patients with CKD and may have positive effects on growth of their fetuses. Therefore, the working group recommends that patients with CKD, who have a nephrological indication for a protein restricted diet, this diet should be continued or started during pregnancy.
In patients with CKD and hemodialysis a high incidence of polyhydramnios was reported early in the second trimester, possibly because of osmotic diuresis in the fetus induced by increased urea. In a retrospective study, an association was found between level of maternal urea and birth weight as well as pregnancy duration. A urea level <17.1-17.5 mmol/L was associated with a high chance of pregnancy duration >32 weeks and birth weight >1500 g, respectively.[31]
In patients with CKD stage 4-5 non dialysis, there is no literature about the association between maternal serum urea and obstetric outcomes, but the working group recommends to hold on this

arbitrary upper level to decrease the risk of polyhydramnios and increase pregnancy duration. When this can only be reached by a protein restriction <0.8 g/kg ideal weight/day amino acid and ketoacid supplements should be considered. Protein intake should regularly be checked by 24h urea excretion and/or the Maroni formula.

<u>Recommendations</u> (no GRADE – very low GRADE)

1. Check sodium excretion in the 24-hour urine of pregnant patients with CKD (non-dialysis (ND)) and limit their daily salt intake to a maximum of 6 grams (= 100 mmol of sodium chloride equivalent to 2400 mg of sodium) as recommended for all CKD patients. (very low GRADE)

2. For pregnant women with CKD and (expected) high degree of sodium retention during pregnancy (manifesting as excessive weight gain combined with edema and/or hypertension), consider further limiting the daily salt intake to 3 grams (1200 mg sodium) during pregnancy. Refer patients to a dietician for this purpose. (very low GRADE)

3. Regularly check the salt excretion of pregnant women with CKD and a good indication for salt restriction by using 24-hour urine or the sodium/creatinine ratio (mmol sodium/10 mmol creatinine) in a spot urine sample. (no GRADE)

4. Continue or start pregnant patients with CKD and a good nephrological indication for protein restriction on a diet with 0.8 g/kg ideal weight/day throughout pregnancy and, therefore, be reluctant to increase protein intake in the second and third trimesters as recommended for healthy pregnant women. This recommendation does not apply to dialysis patients. (no GRADE)

5. For kidney patients with a (highly) advanced CKD stage (G3b-G5ND), consider prescribing a (stricter) protein-restricted diet of less than 0.8 g/kg ideal weight/day during pregnancy to maintain their serum urea level at < 17 mmol/L in order to prevent polyhydramnios, and thereby attain a longer gestational age and a higher birth weight. (no GRADE)

6. Consider supplementing this strictly protein-restricted diet with amino acid supplements to ensure an equivalent daily protein intake of at least 0.8 grams/kg ideal weight/day by referring patients to a dietician with expertise in this area. (no GRADE)

7. Consider regularly checking the urea level in 24-hour urine or a spot urine sample to estimate protein intake and thereby assess dietary compliance on the one hand and avoid excessively low protein intake on the other. (no GRADE)

<u>Knowledge gaps</u>

No randomized studies on the effect of salt and protein restriction in pregnant patient with CKD exist. No studies were found on potassium of phosphate restriction during pregnancy (healthy or CKD). No data were found on the effects of diet restrictions during lactation.

2.2 Treatment of hypertension

Questions

How should pregnant patients with CKD be treated for hypertension during the different trimesters? How does treatment for hypertension (including PE) in pregnant patients with CKD differ from patients without CKD or non-pregnant patients with CKD?

The following subquestions were formulated:

2.2.1 What is the target blood pressure before conception and during pregnancy in patients with CKD with and without proteinuria?

2.2.2 Which diuretic treatment can safely be used to treat sodium retention because of nephrotic syndrome of severe CKD? Which diuretics pass the placenta during the trimesters and could increase the risk of polyhydramnios?

2.2.3 Which antihypertensive drugs are allowed and safe during pregnancy?

2.2.4 Which antihypertensive drugs are preferred in patients with proteinuria before pregnancy and which additional diet interventions are useful before conception and during pregnancy?

2.2.5 Which antihypertensive drugs are preferred during lactation in patients with and without CKD?

2.2.1 Target blood pressure before conception and during pregnancy with and without proteinuria

Background

Chronic hypertension and CKD are two important risk factors for adverse pregnancy outcome, such as preeclampsia, fetal growth restriction, and premature birth. Furthermore, 6 to 31% of the CKD patients develop substantial irreversible kidney function decline after pregnancy, especially in the higher CKD stages.[2,4,6] Probably, glomerular hyperfiltration during pregnancy plays a role in accelerated decrease in kidney function, especially in hypertension,[32] although this is still debated.[2,33,34] Hypertension affects many women with CKD, up to 80% of the patients with end stage CKD.[35] In non-pregnant CKD patients, the aim of antihypertensive therapy is to prevent cardiovascular events and slow down kidney function deterioration.[36] When treating hypertension preconceptionally or during pregnancy, fetal and perinatal outcomes need to be addressed as well. In this context, mother (with and without CKD) and fetus can have conflicting interest. Understandably, there are discrepancies between guidelines about treatment of hypertension in pregnancy and guidelines about treatment of hypertension in CKD.[36,37] Also, there is discussion about the use of angiotensin converting enzyme inhibitors (ACEIs) during the first trimester of pregnancy. Therefore,

there is need for uniformity in the management of hypertension in women with CKD during their pregnancy as well as during the preconception and lactation period.

Search and select, Results, Conclusion

For answering this subquestion, there was no randomized controlled or observational research available that fulfilled the PICO and specifically studied the treatment of hypertension in pregnant women with chronic kidney disease. Advices and recommendations are therefore based on practical experiences and literature or guidelines in other, best suiting populations (pregnant patients without CKD, patients with CKD that are not pregnant).

Rationale

A higher blood pressure (BP) preconceptionally seems to be associated with unfavorable obstetric outcomes for mother and child. However, it is unclear whether this is consequence of the BP or of the underlying disease that causes increased BP. Insufficiently treated hypertension is an important risk factor for negative renal outcome as well. Therefore, the Dutch guideline on CKD recommends to start antihypertensive drugs in all CKD patients with a BP >130/80 mmHg and to strive for a BP <130/80 mmHg because of decrease of cardiovascular risk and because of positive effects on renal outcome.[36,38-40]

In the multicenter CHIPS-trial (Magee et al., 2015) n=987 pregnant women with hypertension but without proteinuria were included (75% preexistent and 25% pregnancy induced).[41] They were randomized for less strict (target diastolic BP 100 mmHg) or strict BP (diastolic BP 85 mmHg). Systolic BP eventually differed 5.8 mmHg (138.8 vs 133.1 mmHg) and diastolic 4.6 mmHg (89.9 vs 85.3). Only incidence of severe hypertension (>160/110 mmHg) differed between groups, 40.6% in less tight group vs 27.5% in the tight groups. There were no differences in other maternal or in fetal outcome measures.

Nielsen et al.[43] included also 10 patients with microalbuminuria and n=7 with diabetic nephropathy in a prospective observational study in n=117 patients with DM type 1 using a low target blood pressure of 135/85 during pregnancy. Positive pregnancy outcomes were found in their cohort. In comparison with diabetic patients from earlier studies using a higher threshold for treating hypertension longer pregnancy duration and higher birth weight were observed. Also, the NICE Guideline 2019 recommends to start with antihypertensive drugs when BP is ≥140/90 mmHg, and in women already on antihypertensive drugs to strive for BP 135/85 mmHg.[43] In pregnancy, the target BP must be as safe as possible for both mother and child. There is no evidence that the target BP must be lower than 130/80 mmHg during pregnancy, amongst others because of risk of hypotension and hypoperfusion of the placenta, amongst others.

Recommendations (no GRADE)

1. When treating hypertension in patients with CKD and a desire to conceive, aim preconceptionally for blood pressure < 130/80 mmHg, regardless of the proteinuria level. (no GRADE)

2. During pregnancy, only initiate antihypertensive treatment in CKD patients (with or without albuminuria) who have not used antihypertensives before or during pregnancy if their blood pressure is higher than 140/90 mmHg on repeated measurements. (no GRADE)

3. In CKD patients who already used antihypertensives before conception, only intensify the antihypertensive regimen during pregnancy if their blood pressure is higher than 140/90 mmHg on repeated measurements. (no GRADE)

4. For CKD patients (with or without albuminuria) who use antihypertensives during pregnancy, aim at a systolic blood pressure between 130-140 mmHg and a diastolic blood pressure between 80-90 mmHg. (no GRADE)

5. After birth, aim at a consistent blood pressure ≤ 130/80mmHg in patients with CKD. (no GRADE)

2.2.2 Diuretics during pregnancy

Background

Diuretic treatment is nephrologically indicated to treat or prevent sodium retention, in particular in patients with proteinuria which can severely increase during pregnancy because of hyperfiltration. It

is still unclear which diuretics are safe during the different trimesters of pregnancy, which diuretics pass the placenta and which diuretics are not safe.

<u>Search and select</u>

A systematic review of the literature was performed.

Question 1:

P pregnant women with CKD

I diuretics

C placebo or no treatment

O preeclampsia, cesarean section, maternal side-effects, perinatal death, stillbirth, neonatal death, premature birth, small for gestational age, gestation at birth, APGAR score

Question 2:

P pregnant women

I diuretics

C placebo or no treatment

O preeclampsia, cesarean section, maternal side-effects, perinatal death, stillbirth, neonatal death, premature birth, small for gestational age, gestation at birth, APGAR score

Relevant outcome measures

The working group considered perinatal death (stillbirth, neonatal death) as a critical outcome measure for decision making, and medication side-effects, preeclampsia (PE), small for gestational age (SGA), premature birth, APGAR score, cesarean section, as an important outcome measure for decision making.

Search and select: methods

The PubMed database were searched with relevant search terms until February 2019. The systematic literature search resulted in 3280 hits. Studies were selected based on the following criteria: RCTs or cohort studies including pregnant women with and without renal disease, where diuretics were compared with no treatment or placebo. 73 studies were initially selected based on title and abstract screening. After reading the full text, 67 studies were excluded and 6 studies were included.

<u>Results</u>

Question 1: No literature was found within the search parameters.

Question 2: Six studies were included in the analysis of the literature.

Summary of literature

Description of studies:

Churchill (2007) is a Cochrane systematic review[44] including five RCTs (from 1962-1984) where treatment with thiazide diuretics to prevent preeclampsia and its complications was evaluated. In total, 1836 healthy women without CKD or hypertension were included who were randomly assigned to either thiazide diuretics, or either placebo or no intervention. Chlorothiazide was used in three studies, hydrochlorothiazide in one study and unspecified thiazide diuretics in the remaining study.

Outcome 1: Preeclampsia

The outcome of preeclampsia was measured in four studies. There was insufficient evidence to demonstrate any clear differences between the two groups in the risk ratio of preeclampsia: RR 0.68 (0.45 to 1.03). While the summary statistic implies a trend towards a reduction in preeclampsia, the trials showed a high level of heterogeneity at 43% and it was a small study that contributed most to the summary statistic.

Outcome 2: Cesarean section

The outcome of cesarean section was measured in one of the five studies. There was no difference in number of Cesarean sections: RR = 1.0; (0.26 to 3.81), heterogeneity (I^2): not applicable.

Outcome 3: Maternal side-effects

The outcome of maternal side-effects such as nausea, vomiting and hypokalemia was measured in one study. More patients with a side-effect were found in the diuretic treatment arm compared to the control arm: RR = 8.70 (1.19 to 63.60), favoring the placebo condition. Women allocated to diuretics were more likely to stop treatment due to unacceptable nausea and vomiting: RR = 1.85 (1.04 to 32.46). Heterogeneity (I^2): not applicable.

Outcome 4: Perinatal death

The outcome of perinatal death was measured in all five included studies. There was no difference in number of perinatal deaths. The pooled effect of fixed effects model was: 0.72 (95% CI 0.40 to 1.27). Heterogeneity (I^2): 0.0%.

Outcome 5: Stillbirth

The outcome of stillbirth was measured in all five included studies. There was no difference in number of stillbirths. The pooled effect of fixed effects model was: 0.60 (95% CI 0.27 to 1.34). Heterogeneity (I^2): 0.0%.

Outcome 6: Neonatal death

The outcome of neonatal death was measured in four studies. There was no difference in number of neonatal deaths. The pooled effect fixed effects model was: 0.88 (95% CI 0.40 to 1.97). Heterogeneity (I^2): 0.0%.

Outcome 7: Premature birth

The outcome of premature births was measured in two studies. There was no difference in number of premature births. The pooled effect fixed effects model was: 0.67 (95% CI 0.32 to 1.41) (ns). Heterogeneity (I^2): 2.0%.

Outcome 8: Small-for-gestational age

The outcome of small-for-gestational age was measured in one study. However, no events were recorded. Therefore, the relative risk cannot be estimated.

Outcome 9: Birthweight

The outcome of birthweight was measured in one study. There was no difference in mean birthweight: MD = 139.00 (-484.40 to 762.40). Heterogeneity (I^2): not applicable.

Outcome 10: Gestation at birth

The outcome of gestation at birth was measured in one study. There was no difference in mean gestation at birth: MD = 0.70 (-0.71 to 2.11). Heterogeneity (I^2): not applicable.

Outcome 11: APGAR-score of less than 7 at 5 minutes

The outcome of the APGAR-score was measured in one study. There was no difference in number of APGAR-scores of less than 7 at 5 minutes: RR = 3.00 (0.14 to 65.90). Heterogeneity (I^2): not applicable.

Level of evidence of the literature

Outcome 1: The level of evidence regarding Preeclampsia was downgraded with 2 levels, due to risk of bias (1) and imprecision (1).

Outcome 2: The level of evidence regarding the outcome measure Cesarean section was downgraded by 3 levels, due to risk of bias (1) and imprecision (2).

Outcome 3: The level of evidence regarding Maternal side effects was downgraded with 2 levels, due to risk of bias (1) and imprecision (1).

Outcome 4: The level of evidence regarding Perinatal death was downgraded with 2 levels, due to risk of bias (1) and imprecision (1).

Outcome 5: The level of evidence regarding Stillbirth was downgraded with 2 levels, due to risk of bias (1) and imprecision (1).

Outcome 6: The level of evidence regarding Neonatal death was downgraded with 2 levels, due to risk of bias (1) and imprecision (1).

Outcome 7: The level of evidence regarding Premature birth was downgraded with 3 levels, due to risk of bias (1) and imprecision (2).

Outcome 8: The level of evidence regarding Small-for-gestational age was not graded, since this outcome could not be estimated.

Outcome 9: The level of evidence regarding Birthweight was downgraded with 3 levels, due to risk of bias (1) and imprecision (2).

Outcome 10: The level of evidence regarding Gestation at birth was downgraded with 3, due to risk of bias (1) and imprecision (2).

Outcome 11: The level of evidence was downgraded with 3, due to risk of bias (1) and imprecision (2).

<u>Conclusion</u>

Cesarean section

**Very low GRADE**	We are uncertain about the influence of diuretics use in pregnant women on number of cesarean sections.<br>*Sources: (Churchill, 2007)*

Maternal side effects

**Low GRADE**	Diuretics use in pregnant women may have a negative influence on the number of times maternal side effects occurred.<br>*Sources: (Churchill, 2007)*

Perinatal death

**Low GRADE**	Diuretics use in pregnant women does not seem to have an effect on number of perinatal deaths.<br>*Sources: (Churchill, 2007)*

Stillbirth

**Low GRADE**	Diuretics use in pregnant women does not seem to have an effect on number of stillbirths.<br>*Sources: (Churchill, 2007)*

Neonatal death

**Low GRADE**	Diuretics use in pregnant women does not seem to have an effect on number of neonatal deaths.<br>*Sources: (Churchill, 2007)*

Birhtweight

**Very low GRADE**	We are uncertain about the effect of diuretics use in pregnant women on mean birthweight.<br>*Sources: (Churchill, 2007)*

Gestation at birth

**Very low GRADE**	We are uncertain about the effect of diuretics use in pregnant women on mean gestation at birth.<br>*Sources: (Churchill, 2007)*

APGAR score

**Very low GRADE**	We are uncertain about the effect of diuretics use in pregnant women on number of APGAR-scores of less than 7 at 5 minutes.<br>*Sources: (Churchill, 2007)*

Rationale

Use of diuretics became controversial because they reduce plasma volume and therefore theoretically might decrease placenta perfusion with a higher risk for maternal and fetal complications.[45,46] Evidence on us of diuretics in pregnancy is restricted to older cohort studies and small trials with the goal to prevent PE. The level of evidence of the recommendations is low.

Prevention of PE

A Cochrane review describes the effect of thiazide diuretics on prevention of PE in healthy women without hypertension or CKD compared to placebo.[44] None of the relevant pregnancy outcomes differed between groups. This review does not support prescription of diuretics for prevention of pregnancy-related hypertensive disorders. On the other hand, these placebo-controlled studies did not show any harmful effect on perinatal mortality or preterm birth.

Maternal side-effects

Churchill (2007) describes an increased risk for maternal side-effects with diuretics although these were not severe.[44] More women had to stop use of diuretics compared to the placebo group because of side-effects.

Theoretically, in case of (impending) preeclampsia diuretics can lead to further plasma volume depletion which activates RAS system and and possibly lead to progressive hypertension. Furthermore, there is the possibility of diminished placenta perfusion with theoretical harm for the fetus.[47]

Risk for neonatal complications/congenital defects

Most diuretics pass the placenta. A large Danish/Scottish cohort did not show any association between loop or thiazide diuretics and congenital defects.[48] A few case reports reported on neonatal thrombocytopenia, hypoglycemia, hypovolemia and jaundice.[49] The theoretical risk of polyhydramnios is not described in studies. Loop and thiazide diuretics are safe during lactation, although in high dosages they can diminish breast milk production.[50]

Literature on potassium sparing diuretics is scarce and restricted to amiloride which seems safe.[51,52]

Triamterene should definitely not be used during the first trimester because of its antagonistic effect on folic acid.[53] Spironolactone should not be used because of the anti-androgenic effect on the fetus.[54]

Because of the theoretical effect of hypoperfusion of the placenta during use of diuretics, it is recommended to regularly evaluate fetal growth.

Indications for diuretics in pregnant women with CKD

When there is a nephrological indication to continue use of diuretics and no alternative treatment is available, it can be considered to prescribe diuretics preconceptionally and continue them during pregnancy.

<u>Recommendations</u> (no GRADE – low GRADE)

1. Consider prescribing diuretics to CKD patients with a desire to conceive only if:

- their blood pressure doesn't reach the desired target <130/80 mmHg with the central alpha-blocker methyldopa, beta blockers and/or calcium channel blockers due to (possible) sodium retention caused by their CKD;
- their proteinuria is higher than 0.5 grams/day in order to reduce the likelihood of sodium retention and associated hypertensive disorders during pregnancy. (low GRADE)

2. Do not prescribe diuretics to CKD patients before conception:

- to prevent pregnancy-specific hypertensive disorders (including preeclampsia),
- as preferred treatment of (essential) hypertension and nephrogenic hypertension without relevant proteinuria. (low GRADE)

3. Only start thiazide or loop diuretics in pregnant CKD patients if there is a strict indication. (low GRADE)

4. Avoid starting diuretics if preeclampsia is suspected since preeclampsia is usually due to excessive vasoconstriction with intravascular underfilling. This may be aggravated by diuretics, with a potentially negative effect on placental perfusion. (no GRADE)

5. CKD patients should continue to take thiazide diuretics during pregnancy if they were started before pregnancy *and* there is no good alternative. (low GRADE)

6. Only if there is a strict indication for potassium-sparing diuretics, consider prescribing amiloride during pregnancy. Avoid triamterene or aldosterone antagonists (e.g. spironolactone) during pregnancy due to the risk of severe teratogenic effects. (no GRADE)

Knowledge gaps

Does continuation of diuretics in pregnant women with nephrogenic hypertension lead to less pregnancy complications compared to medication change preconceptionally?

2.2.3 Safety of antihypertensive drugs used in pregnancy

Search and select

An exploratory analysis of the literature was performed to answer the following question:
What are the effects of the pharmaceutical treatment of hypertension compared to expectative management in pregnant women with CKD and hypertension?

P Pregnant women with CKD and hypertension

I anti-hypertensive medication

C expectative management

O prevention of hypertensive crisis/maternal complications (mortality and morbidity), hospitalization, decline in kidney function

Relevant outcome measures

The working group considered:
-maternal: mortality and prevention of hypertensive crisis/eclampsia/HELLP critical maternal outcome measures for decision making; and morbidity, hospitalization and decline in kidney function as important outcome measures for decision making;
-fetal: intra-uterine fetal death and congenital abnormalities critical fetal outcome measures; and intra-uterine growth restriction and APGAR score <7 after 5 min as important fetal outcome measures.

Search and select: methods

We searched for randomized controlled trials or systematic reviews or observational studies that studied the safety profile, side-effects and association with congenital anomalies of various antihypertensive agents in pregnant women with CKD. Based on title and abstract, initially 141 studies were selected. After examination of full text all studies were excluded, since none reported the correct patient population (CKD).
A description of studies that reported the safety profile, side-effects and association with congenital anomalies of various antihypertensive agents in pregnant women overall is reported in this summary. The description of the studies below is limited to articles published after finishing the Dutch Multidisciplinary guideline "*Hypertensive disorders in Pregnancy*" in 2011 because this guideline was used as starting point for answering the current question. A total of 8 studies are described below.

<u>Results</u>
No study exactly answered the search question. N=6 studies were useful for answering the search question and are described below.

Description of studies
Yakoob (2013)[55] performed a meta-analysis to determine teratogenicity of β-blockers in early pregnancy. After a systematic literature search meta-analyses were performed using random-effects models based on odds ratios (ORs). Prespecified subgroup analyses were performed to explore heterogeneity. Randomized controlled trials or observational studies examining risks of congenital malformations associated with first trimester β-blocker exposure compared with no exposure were included. Thirteen population-based case-control or cohort studies were identified. Based on meta-analyses, first-trimester oral β-blocker use showed no increased odds of all or major congenital anomalies (OR=1.00; 95% confidence interval, 0.91 to 1.10; 5 studies). However, in analyses examining organ-specific malformations, increased odds of cardiovascular defects (OR=2.01; 95% confidence interval, 1.18 to 3.42; 4 studies), cleft lip/palate (OR=3.11; 95% confidence interval, 1.79 to 5.43; 2 studies), and neural tube defects (OR=3.56; 95% confidence interval, 1.19 to 10.67; 2 studies) were observed. The effects on severe hypospadias were nonsignificant (1 study). Causality is difficult to interpret given the small number of heterogeneous studies and possibility of biases.

Bergman (2018)[56] investigated whether first-trimester use of beta-blockers increased the risk of specific congenital anomalies. They performed a population-based case-malformed control study, conducted in 117,122 registrations of congenital anomalies from 17 European (EUROCAT) registries in the period between 1995 and 2013. EUROCAT registries collect data on all pregnancy outcomes:

livebirths, fetal deaths ≥ 20 weeks of gestational age and terminations of pregnancy for fetal anomalies with a major congenital anomaly. Bergman (2018)[56] performed a systematic literature review to identify associations previously reported on maternal first trimester use of beta-blockers and congenital anomalies. These associations were tested, and an exploratory analysis was performed to identify new signals. Odds ratios of exposure to any beta-blocker or to a beta-blocker subgroup were calculated for each anomaly compared to two control groups (non-chromosomal, non-signal anomaly compared with all the anomalies).
A total of 320 congenital anomaly cases were exposed to beta-blockers. The selective beta-blockers (metoprolol, atenolol, bisoprolol) were most widely used (in 45.3%), followed by labetalol (31.6%). There was no information on medication dose and duration of use. The authors found no increased odds for congenital heart defects, oral clefts, neural tube defects and hypospadias after first trimester betablocker use, but they did reveal increased odds of 3.8 (95%-CI 1.3 to 11.0) for multi-cystic renal dysplasia (MCRD) after maternal exposure to labetalol. However, the absolute risk for MCRD for offspring of women using labetalol would be very low (less than 1 in 500). This new finding needs further investigation in a prospective study. A limitation of the study was that the control group was malformed as well, which makes that the ORs are relative to other malformations and may therefore not be translated directly to the general population.

Hoeltzenbein (2017)[57] performed a prospective observational cohort study to analyze the rate of major birth defects and spontaneous abortions in women with methyldopa therapy for chronic hypertension. Outcomes of 261 pregnancies with first trimester exposure to methyldopa and 526 randomly selected comparison pregnancies without chronic hypertension were evaluated.
Prospective pregnancies with chronic hypertension and exposure to methyldopa in the first trimester were included in the cohort at risk. The median daily dose was 500 mg. 54% had started methyldopa before conception. Methyldopa was used as single antihypertensive drug in the first trimester in 49% of the pregnancies.
Patient characteristics were different at some points: the methyldopa cohort had a higher BMI compared with the controls (28 versus 23 kg/m^2) and a lower rate of smokers (8% versus 17% in the controls). As expected, the methyldopa group had a higher risk of preeclampsia, gestational diabetes mellitus and caesarian section. The rate of major birth defects in the methyldopa cohort was not significantly increased compared with the comparison cohort (3.7% versus 2.5%; adjusted OR, 1.24; 95%-CI 0.4 to 4.0). Among the 9 methyldopa-exposed mothers with children with major birth defects, 2 mothers had preexisting diabetes mellitus type 2. There was a tendency toward a higher rate of spontaneous abortions in methyldopa-exposed women (both in normal range). Adjusted birth weight scores were significantly lower in the methyldopa group.

To account for possible disease-related effects, a subgroup of pregnancies with methyldopa monotherapy (n=127) was compared with pregnancies with metoprolol monotherapy (n=215) in the first trimester. In this sensitivity analysis, there was no evidence for an increase in birth defects, early fetal loss or growth restriction in the methyldopa group compared with the metoprolol group.
In conclusion, this study does not indicate a teratogenic risk of methyldopa. However, because of the limited sample size, the authors were only able to detect a 2.5-fold increased risk of major birth defects in the exposed cohort. Another main discussion point is the teratogenic potential of the underlying chronic hypertension and its comorbidities associated, like high BMI and diabetes mellitus. To disentangle effects of hypertension and medication, a nonexposed hypertensive comparison cohort would be desirable. However, similar to other chronic diseases, untreated patients are probably less severely affected and, therefore, present different disease characteristics.

Kahn (2010)[58] performed a systematic review and meta-analysis to evaluate the fetomaternal safety of calcium channel blockers (CCB) in pregnancy in the context of treatment of pregnancy induced hypertension or as a tocolytic agent in case of imminent spontaneous preterm birth (PTB). Systematic searches were performed between 1983 and 2005. Data were extracted from the full articles, by independent reviewers. Quantitative adverse events rates calculations were restricted to series of women given CCBs identified from RCTs and observational studies, not from case series. Meta-regression with generalized estimation equations modelling explored reasons for heterogeneity, seeking factors increasing the rates of the most commonly reported adverse events. Of 269 relevant reports, including 5607 women, adverse fetomaternal events varied according to the total dose of nifedipine and study design. The main CCBs administered were nifedipine (3685 women); verapamil (475 women); and nicardipine (368 women). Quantitative data analysis was possible for 197 reports. Adverse events were highest amongst women given more than 60 mg total dose of nifedipine (OR 3.78, 95%CI 1.27 to 11.2, p = 0.017) and in reports from case series compared to controlled studies (OR 2.45, 95% CI 1.17 to 5.15, p = 0.018). Headache, flushing, nausea and vomiting were most frequently reported. Reported maternal adverse events considered to be potentially fatal were myocardial infarction; palpitations, pulmonary edema, cyanosis, hypoxia. Reported fetal or neonatal adverse events considered to be potentially fatal were respiratory distress, non-reassuring fetal heart rate patterns, cardiomyopathy, acidosis, and renal failure.
The main limitations of this study were the heterogeneity and a lot of lacking data of the studies included in the review. Dose administered was stated in 68.0% of the reports, gestational age in 62.5% and duration of treatment only in 30.5%. When it was stated, in 55% CBB-treatment duration was less than 24 hours. The indication for starting CCB (PTB, hypertension, or fetal tachycardia) was not described in the review but seemed to be for preventing PTB in the vast majority of the cases and

will probably be restricted to late second and third trimesters of pregnancy. Treatment regime of CCB in preconceptional or pregnancy-induced hypertension is usually totally different than when treating PTB. This makes interpretation of the results to guide treatment of hypertension in daily practice quite difficult. However, this systematic review does demonstrate that CCBs in pregnancy, especially a high dose of nifedipine, are associated with a wide range of fetomaternal adverse events that could also be at least partially related to the underlying disease necessitating the start of these drugs.

Magee (2016)[59] did a secondary analysis on the data of the CHIPS trial cohort[41] comparing pregnancy outcomes between methyldopa- and labetalol-treated women, while accounting for allocated group ("less tight" versus "tight" control of hypertension). Recruited patients were women between 14^{+0} to 33^{+6} weeks gestation with non-proteinuric pre-existing or gestational hypertension and office diastolic blood pressure 90 to 105 mmHg. At randomization, 243 (25%) and 238 (24%) of the 987 women enrolled used methyldopa and labetalol respectively. Post-randomization, this was 23% on methyldopa and 44% on labetalol of the 981 women included in the randomized study (labetalol was the recommended antihypertensive, but women could stay on their existing antihypertensive agent). Logistic regression was performed to compare outcomes among women who took methyldopa or labetalol, adjusting for stratification factors (for example hypertension type) and key prognostic factors (for example antihypertensive therapy, systolic blood pressure and gestational age at randomization, BMI, ethnicity, parity, age, diabetes).

Composite primary outcome was pregnancy loss or high-level neonatal care for > 48 hours in the first 28 days of life. The authors found that methyldopa (versus labetalol) at randomization was associated with fewer babies with birthweight < 10th percentile (aOR 0.48; 95% CI 0.20 to 0.87). Post-randomization, methyldopa was associated with decrease in the primary outcome (aOR 0.63, 95%CI 0.40 to 1.00) and babies with birthweight < 10th percentile (aOR 0.54, 95% CI 0.32 to 0.92), but maternal outcomes were also improved in mothers continuing methyldopa: severe hypertension (aOR of 0.51, 95% CI 0.31 to 0.83), preeclampsia (aOR of 0.55, 95% CI 0.36 to 0.85), and delivery at < 34 weeks (aOR of 0.53, 95% CI 0.29 to 0.96) or < 37 weeks (aOR of 0.55, 95% CI 0.35 to 0.85). Sensitivity analyses accounting for a switch from methyldopa or labetalol to another drug post-randomization showed an attenuated impact of methyldopa on outcomes (results were no longer significant). Particularly, those treated with methyldopa with pre-existing hypertension had better outcomes.

The most important weakness of this study is that it is a non-randomized comparison and high risk of bias by indication. Although the authors adjusted for many prognostic factors, the results are still subject to residual confounding. Since methyldopa had the name to be a "less robust" antihypertensive agent, it could have been that clinicians prescribed methyldopa to women with the

mildest disease profile. For this reason, the authors conclude that pregnancy outcomes with methyldopa are probably at least as good as with labetalol.

Diav-citrin (2011)[60] performed a prospective observational cohort study to examine first trimester safety of angiotensin-converting-enzyme-inhibitors (ACEIs) and angiotensin-receptor-blockers (ARBs). They included pregnant patients who contacted two Teratology Information Services in Israel (1994 till 2007) and Italy (1990 till 2008) about their exposure to ACEIs or ARBs. The rate of major congenital anomalies in offspring of patients with first trimester exposure to RAS blockade was compared with two groups: (1) exposed to other antihypertensives (OAH), and (2) after non-teratogenic exposure (NTE) in similar time frames. The distribution of the OAHs was: betablockers 60.1%; calcium channel blockers 20.7%; methyldopa in 12.5%; thiazides in 5.1%.

A total of 252 ACEI/ARBs-exposed (89% ACEIs), 256 OAH-exposed and 495 NTE-exposed pregnancies were followed-up. Indications for the treatment with ACEI/ARBs were: hypertension (76.3%), kidney disease (10.3%), cardiac disease (5.4%), SLE (4.9%), and other (3.1%). Median gestational week of ACEI/ARB discontinuation was 6 (75% between week 5 to 9).

There was a significantly higher rate of pre-gestational diabetes in the ACEI/ARB group (11.9%) compared to the OAH group (3.9%), more women were treated with lipid lowering agents in the beginning of their pregnancy (i.e. statins) (ACEI/ARB 12.7% versus OAH 2.0%, $p < 0.001$) and more had additional antihypertensive medications during pregnancy (ACEI/ARB 54.0% versus OAH 27.3%, $p < 0.001$). The rate of miscarriages and elective terminations of pregnancy was higher in the ACEI/ARB group compared to the NTE-group.

The rate of major congenital anomalies was comparable between the three groups (12/193, 6.2%, ACEI/ARB; 10/213, 4.7%, OAH; 22/471, 4.7% NTE; $p=0.688$). After excluding those with chromosomal /genetic anomalies or related to other known teratogens, including elective termination of pregnancy due to prenatally diagnosed anomalies, the rate of major congenital anomalies was still comparable between the 3 groups (4.2% 8/190 in ACEI/ARB; 4.2% 9/212 in OAH; 3.8% 18/471 in NTE; $p = 0.954$). For the most common ACEI/ARBs the rate of major congenital anomalies was: enalapril 1/63 (1.6%), ramipril 3/47 (6.4%), cilazapril 3/13 (23.1%), captopril 0/10 (0%), fosinopril 0/10 (0 %), quinapril 1/6 (16.7%), losartan 0/9 (0%), lisinopril 0/7 (0%). The mean birth weight was lower and rate of preterm delivery higher in the ACEI/ARB and OAH groups.

This study supports that ACEI/ARBs in the first trimester of pregnancy do not represent a major teratogenic risk. Due to the limited sample size of 190, they were only able to detect a 2.6-fold increase for major anomalies. The higher rate of miscarriages, the earlier gestational age at delivery, the higher rate of preterm deliveries, and the lower birth weight in the ACEI/ARB group may be associated with these drugs, other medications, the underlying disease, or a combination of all three

factors. The fact that similar changes were seen in the OAH group supports the association with the underlying disease.

Polifka (2012)[61] performed a critical review of the literature on the association between first-trimester exposure to ACEIs or ARBs and development of embryopathy. Because adequate functioning of the RAS is essential for normal fetal kidney development, embryopathy can be expected when these drugs are used during pregnancy and the potential for ACEIs and ARBs to impair fetal and neonatal renal function if taken after the first trimester of pregnancy has been well documented. Although these drugs were not found to be teratogenic in animals, until recently little was known about the teratogenic effects of ACEIs and ARBs in humans when exposure was limited to the first trimester of pregnancy. New evidence from epidemiologic studies indicates that there may be an elevated teratogenic risk when these drugs are taken during the first trimester of pregnancy. However, this elevated risk does not appear to be specific to ACEIs and ARBs but is instead related to maternal factors and diseases that typically coexist with hypertension in pregnancy, such as diabetes, advanced maternal age, and obesity. Women who become pregnant while being treated with an ACEI or ARB should be advised to avoid exposure to these drugs during the second and third trimesters of pregnancy by switching to a different class of antihypertensive drugs after 8 and 10 weeks after conception.

Opperman (2012)[62] aimed to assess the risk of fetopathy, the sensitive time window and possible new symptoms in prospective and retrospective cases with ARBs treatment during the second and third trimester of pregnancy.
Patients were enrolled by the Berlin Institute for Clinical Teratology in Pregnancy (1999 till 2011) through risk consultation. Symptoms indicative of ARB fetopathy were defined as: oligo-/anhydramnios, renal insufficiency, lung hypoplasia, joint contractures, skull hypoplasia and fetal or neonatal death.
Authors included 29 prospective cases, all treated for hypertension. In most cases, treatment was started before pregnancy and continued after the first trimester, due to late pregnancy diagnosis or unawareness of the risk. In 5/29 (17.2%) oligo/anhydramnios was observed, but reversible after ARB withdrawal. In all the 5 cases, the ARB was used at least until 20 weeks of pregnancy. Two infants showed additional symptoms of fetopathy. The risk is more than 30% if treatment continues beyond the 20th week of pregnancy. Among 16 retrospective case reports, 3 infants presented with a thrombosis of the inferior caval vene in the vicinity of the renal veins. Four out of 13 live births did not survive. This survey suggests that the risk increases with duration of ARB treatment beyond the first trimester and oligo-/anhydramnios may be reversible after discontinuation. ARB medication

during second and third trimester of pregnancy constitutes a considerable risk and must be discontinued immediately.

Conclusion

- GRADE	No studies were found that reported the effects of the pharmaceutical treatment of hypertension compared to expectative management in pregnant women with chronic kidney disease (CKD) and hypertension.

Rationale

Methyldopa, labetalol and nifedipine are still the drugs most often prescribed in the second half of pregnancy because of much practical experience and because of their safety. Table 1 presents considerations for use of antihypertensive agents before and during pregnancy.

- Methyldopa: prospective studies show no increased risk for congenital defects nor for growth restriction, also when started preconceptionally. [57,63-65] A secondary analysis in the CHIPS-trial showed that methyldopa started before the 2nd trimester leads to lower incidence of small for gestational age babies than labetalol.[59] Choice of antihypertensive drugs was made by the treating physician and the data suggest that there was selection bias by indication. Methyldopa may be less effective in prevention or treatment of severe hypertension compared to labetalol.[66] Approximately 17% stops methyldopa because of central side-effects and there may be a risk for postpartum depression.[67,68] Furthermore, preconceptionally methyldopa may lead to hyperprolactinemia with amennorhoea.[69]
- Labetalol and other beta blocking agents: there is no increased risk of congenital defects.[55,56] Labetalol and metoprolol are preferred during pregnancy, and also bisoprolol may be safe.[70] However, these drugs may be associated with higher incidence of SGA, although it is hard to conclude whether the growth restriction is a consequence of use of the beta-blocking agent or of the underlying disease which is also associated with fetal growth restriction.[71]
- Calcium antagonists: most experience exists with nifedipine which is therefore recommended in most guidelines.[37,43,72] Small studies showed no teratogenicity for nifedipine or amlodipine when used in the first trimester.[59,73,74] Nifedipine has a positive effect on prevention and treatment of severe hypertension in the 2nd half of pregnancy.[65,66] Short working agents should not be used because of unpredictable BP lowering effect and thereby also placental flow.[75] Severe side effects are rare and mainly seen with higher dosages that are needed for patients with more severe underlying complications.[58] Because almost all studies started nifedipine or amlodipine in (late) 2nd trimester for impending preterm birth or in 3rd trimester for pregnancy induces

hypertension or PE, evidence is weak that these agents are safe in first trimester, but they may be started when no alternatives are available.

- Diuretics: see 2.2.2
- RAS inhibitors (RASIs) (ACE inhibitors (ACEi), angiotensin receptor blockers (ARBs) and direct renin inhibitors): all have fetotoxic effects when used during the 2nd and 3rd trimester and lead to a severely increased risk for diverse congenital defects (heart, kidneys and central nervous system of the fetus). Furthermore, it has been postulated that RASIs lead to fetal hypotension during the 2nd and 3rd trimester, which leads to tubular pathology, anuria and oligohydramnios, and the consequent fetal complications (e.g. contractures and lung hypoplasia).[62,76] Use in the 2nd and 3rd trimester should be avoided, with a possible exception for short temporary use for renal crisis in scleroderma in the 3rd trimester.[77] The recommendation to continue RASIs preconceptionally can be considered in patient groups that have great health gain by these drugs, e.g. patients with CKD and diabetes mellitus and/or substantial proteinuria.[36,43,78,79] Three studies show no evident increased risk for congenital defects with (early) exposure in the first trimester, and the underlying disease seems to be responsible for the possible slightly increased risk for congenital (heart) defects.[60,61,80]

<u>Recommendations</u> (Table 1; no GRADE)

1. Choice of antihypertensives:

- When prescribing methyldopa preconceptionally, be aware on the one hand of the possibility of amenorrhoea due to hyperprolactinaemia and the other hand central side effects such as lethargy, dizziness and sadness making it a less attractive drug in patients with (a history of) depression.
- If a beta-blocker must be prescribed, select an agent that is considered safe (labetalol, pindolol, metoprolol, or bisoprolol).
- Be very careful when prescribing short-acting dihydropyridine calcium antagonists such as direct-acting nifedipine and starting high doses in short periods.

  There is only limited evidence that dihydropyridine calcium antagonists such as nifedipine and amlodipine are safe in the first trimester. However, case series and extensive obstetrical

experience have revealed no serious problems. Such agents can be prescribed preconceptionally if there are no good alternatives.

- Stop RAS inhibitors (RASIs) either preconceptionally or early in the first trimester, certainly before 8 weeks of amenorrhoea, since RASIs can cause severe congenital defects and oligohydramnios, due to fetal anuria induced by fetal hypotension.
- Only recommend continuing RASIs in women with a desire to conceive if they are sufficiently aware of needing to stop taking the drug immediately if two consecutive pregnancy tests are positive (no later than 8 weeks of amenorrhoea) *and* have a strong indication, that is:
    - diabetic nephropathy with at least moderate albuminuria (A2, ACR ≥ 3 mg/mmol),
    - CKD and severely elevated albuminuria (A3, ACR ≥ 30 mg/mmol, corresponding to proteinuria ≥ 0.5 g protein/24 hours or protein/creatinine ratio ≥ 0.5 g/10 mmol creatinine).
- Even before pregnancy, consider testing which combination of non-RASI antihypertensives is effective and well-tolerated to ensure that the correct combination can be prescribed immediately on pregnancy confirmation.

2.2.4 Antihypertensive drugs and diet interventions in patients with proteinuria preconceptionally

<u>Background</u>

Women with hypertension and proteinuria have an increased cardiovascular risk profile. Moderate increased albuminuria (A2) is associated with left ventricular hypertrophia, vascular disease, and disturbances of lipid profile and glucose tolerance.[81,82] In DM type 1 and 2 moderate severe albuminuria (A2) is a good predictor for diabetic nephropathy, end-stage kidney disease and negative cardiovascular outcome.[83] Patients with a sufficient treated BP (≤ 130/80 mmHg) have less cardiovascular events, less decrease in kidney function and longer preservation of kidney function. [38,84] Severeness of proteinuria is also an important risk factor for kidney failure. Treatment of hypertension and proteinuria is crucial for renal as well as cardiovascular survival in patients with CKD.

Search and select, Results, and Conclusion

For answering this subquestion, we did not find any randomized controlled trial or observational study in the 1591 articles from our systematic search that specifically studied the possible advantages of different antihypertensive drugs on maternal and fetal outcome in pregnant patients with CKD and proteinuria. Advices and recommendations are therefore based on literature, and/or guidelines in other, best suiting populations (pregnant patients without CKD, patients with CKD that are not pregnant), and practical experiences.

Rationale

Outside pregnancy, RASIs are preferred in treatment of hypertension in CKD, in particular in patients with DM type 1 or 2 and nephropathy, and also in patients with CKD and proteinuria, because of their positive effects on renal and cardiovascular survival. Their positive effect can be intensified by adequate salt restriction (<2400 mg sodium/day) and/or addition of a thiazide diuretic.[85,86]

Preconception treatment of hypertension in patients with CKD and severe increased albuminuria.

Because an important part of hypertension in patients with CKD with severe increased albuminuria and/or later stages of CKD is a consequence of sodium retention, it is useful to start preconceptionally with a sodium restricted diet (<2400 mg/day). Furthermore, it seems sensible to start with a thiazide diuretic when BP >130/80 mmHg. With hydrochlorothiazide is obstetrically the most experience.

Timing of stopping RAS inhibitors in patients with CKD with severe albuminuria and pregnancy wish.

The preconceptional phase has an unknown duration and therefore it is unattractive to withhold women with severe albuminuria their RASI.[87] The RASI can be stopped when pregnancy test is positive and at least before amenorrhea duration of 8 weeks. It is useful to test before pregnancy which antihypertensive medication without RAS inhibitor is effective and well tolerated. Proteinuria without RASI can then also be measured.

Treatment of hypertension in patients with CKD with severe albuminuria or nephrotic syndrome during pregnancy

In most patients with CKD and proteinuria because of glomerular diseases the proteinuria will increase particularly during the 3rd trimester.[6] Blood pressure particularly increases in the 3rd trimester in patients with preexisting hypertension, and other way around, increase of proteinuria can lead to sodium retention and further increase of blood pressure.[88] Treatment starts with more strict sodium restriction (<1400 mg/day) and with nephrotic range proteinuria also a more strict

protein restriction (0.6-0.7 g/kg ideal weight/day supplemented with amino and keto acids).[29,30] When insufficient effect is reached on blood pressure of proteinuria and the patient already uses hydrochlorothiazide, amiloride can be relatively safely added after 20 weeks gestational age.

Recommendations (no GRADE)

1. In CKD patients with proteinuria who develop nephrotic range proteinuria (> 3 grams/day) during pregnancy, consider one or more of the following measures:

- further restricting the dietary sodium intake to approximately 1200 mg/day;
- limiting the daily protein intake to 0.6 to 0.7 grams/kg ideal weight, supplemented with amino and keto acids equivalent to 0.2 to 0.3 grams of protein/kg ideal weight;
- adding the potassium-sparing diuretic amiloride in patients who already use a diuretic and develop (severe) sodium retention from the 20th week onwards, because of its beneficial effect on sodium retention caused by proteinuria;
- starting with a dihydropyridine calcium antagonist only if the patient's systolic blood pressure is above 140 mmHg or diastolic blood pressure above 90 mmHg on repeated measurement despite the maximum dose of the thiazide diuretic and beta-blocker.

2.2.6 Antihypertensive drugs during lactation

Background

Most women with CKD have chronic hypertension that persists after birth and often worsens on day 3-6 postpartum because of mobilization of fluid from extra- to intravascular compartment.[89] Antihypertensive drugs can most often safely be used during lactation, because most drugs arrive in the breast milk only in very small amounts.

Search and select, Results, Conclusion

The working group did not perform a systematic search for this clinical question. The expectation was that no literature would be found that specifically studied the possible neonatal risks of the use of different anti-hypertensive drugs in lactating patients with CKD. Advices and recommendations are

based on literature or guidelines in other, best suiting populations (such as lactating patients with hypertension but without CKD), and practical experiences.

Rationale

Most antihypertensive drugs have not been investigated in lactating women in controlled studies. The scarce available studies are not specific for CKD. All studies included term born neonates. All studies found that the older the neonate, the lower the relative child dose (medication level), and the lower risk of effect of the drug. On theoretical grounds, precaution must be taken in (extreme) premature babies and extra monitoring of blood pressure and heart frequency can be necessary. The advantages of breast feeding (less necrotizing enterocolitis, sepsis and mortality) outweigh the risks most often in this group.[90] Supplementary Table S1 presents considerations for use of antihypertensive agents during lactation.

RAS inhibitors

Short acting RASIs captopril and enalapril are considered safe.[43,91-94] Other RASIs are not recommended during lactation because of lack of evidence.[95]

Diuretics

Diuretics are not preferred during lactation because they can decrease milk production and give complaints of excessive thirst.[96,97] Chlorotalidone should not be used because it can accumulate in the neonate because of its long half-life. Spironolactone can safely be used on strict indication. The risk for gynecomastia seems small for male neonates.

Methyldopa

Methyldopa is safe during lactation but is not preferred because of association with postpartum depression and no once daily regimen.[98]

Calcium antagonists

Calcium antagonists barely arrive in breast milk and can safely be used.[97,99,100]

Beta-blocking agents

Labetalol, pindolol and propranolol have a high degree of protein binding and therefore diffuse barely in the milk and are relative safe for the neonate.

Recommendations (no GRADE)

1. For women who need an antihypertensive during lactation, the potential advantages and disadvantages of the various agents must be considered.

- When there is proteinuria > 0.5g/24h or diabetic nephropathy, start with enalapril if antihypertensive treatment is indicated, as soon as possible after birth.
- For women with CKD who need a beta-blocker during lactation, choose a beta-blocker with favorable pharmacokinetic properties (high protein binding, low kidney excretion: labetalol, metoprolol, pindolol, or propranolol) since these can be safely used during lactation.
- Avoid prescribing alpha-blockers during lactation because it is largely unknown whether these drugs find their way into breast milk and the neonatal risks are still unknown.

2.3 Treatment of anemia

Questions:

How should anemia be treated in a pregnant patient with CKD?

1. Are target values for iron parameters different in CKD during pregnancy?
2. What are the conditions for treatment with parenteral iron preparations?
3. In which trimesters can the patient safely be treated by erythropoietin (EPO) and what is the hemoglobin (Hb) level targeted for?
4. What is the cut-off for Hb for treatment with a blood transfusion and which precautions should be taken?

Background

During pregnancy circulating volume increases by 40-50% while erythrocytes only increase by 25%. During healthy pregnancy EPO production increases 2-4 times the concentration of non-pregnant experimental subjects from the first trimester.[100-103] EPO peak concentration is in the third trimester, but is also postpartum still increased.[104] Furthermore, fetus and placenta need iron which leads to an increased iron need of 1-2mg normally to 7.5mg per day during third trimester.[105] This increased need cannot be met by a physiological increased uptake in the gut. Net iron balance therefore is negative during pregnancy, birth and postpartum.[106] All this may lead to anemia, while patients with CKD already often have anemia because of iron deficiency, EPO deficiency, medication's side effects and diet restrictions.[36]

Anemia during pregnancy increases the risk for prematurity, low birth weight, placenta praevia and premature placenta abruption.[87,106-112]

Search and select

A systematic review of the literature was performed to answer the following subquestion:

What are the beneficial and harmful effects of treating anemia in a pregnant patient with chronic kidney disease, compared to not treating the anemia?

P: pregnant patient with chronic kidney disease;

I: oral iron suppletion, intravenous iron suppletion, erythropoietin (rhEPO - recombinant human erythropoietin), blood transfusion;

C: no treatment, iron +/- rhEPO;

O: increase in Hb, increase in reticulocyte count.

Relevant outcome measures

The guideline development group considered congenital abnormalities as a critical outcome measure for decision making; and live birth rate, low birth rate as an important outcome measure for decision making.

Search and select: methods

The PubMed database was searched with relevant search terms until 15th March 2019. The systematic literature search resulted in 538 hits. 81 studies were initially selected based on title and abstract screening by two independent members of the working group. After reading the full texts, none of the studies could be included because none of the studies compared treatments for anemia in women with renal disease.

Results

No literature was found within the search parameters.

Literature summary

Not applicable. No studies were found that answered the search question.

Conclusion

Not applicable. No studies were found that answered the search question.

Rationale

1. *Are target values for iron parameters different in CKD during pregnancy?*

Most frequent cause of anemia during pregnancy is iron deficiency which is estimated to occur in >40% of pregnancies.[87] No RCTs have been performed on target values of iron parameters during pregnancy in CKD. The Dutch guideline working group found no evidence to follow separate iron target values in CKD stage 1-2 than in the general population: when iron supplementation is needed to aim for a ferritin >80 mcg/L in pregnancy (Dutch national guideline Blood Transfusion). For CKD stage 3-5 the target should be transferrin saturation (TSAT) >30% and ferritin >200 mcg/L (maximum 500 mcg/L) In pregnant dialysis patients the target should be TSAT 30-50% and a ferritin >300 mcg/L (maximum 800 mcg/L).[36,113-114]

2. *What are the conditions for treatment with parenteral iron preparations?*

No RCTs have been performed on the subject of parenteral iron preparations in pregnancy in CKD. Two RCTs and a prospective observational study have been performed on pregnant women with iron

deficiency anemia but without CKD.[115-117] In these studies ferric sucrose and ferricarboxymaltosis were effective in correcting anemia, safe for the mother and characteristics of the neonate were similar compared to oral ferrosulphate. However, parenteral iron preparations have a little risk for allergic reactions.[87] Ferric sucrose passes the placenta and may lead to fetal iron accumulation. Therefore, reduced dosing in pregnant dialysis patients is advised (62.5-100 mg).[101] Teratogenicity or neonatal effects have not been reported, but has unknown risk in the first trimester. It has been reported to be probably safe when given during breastfeeding period.[87,103] Ultimately, oral iron preparations are safe, cheap and easily available and therefore should be first line of treatment.[114]

3. *In which trimesters can the patient safely be treated by erythropoietin (EPO) and what is the hemoglobin (Hb) level targeted for?*

No RCTs have been performed on this subject.

Deficiencies of iron, folic acid and vitamin B12 have to be excluded and adequately treated before considering prescribing rhEPO. In animal studies in monkeys and sheep in the last part of pregnancy and in ex-vivo human placenta perfusion experiments has been shown that rhEPO does not pass the placenta.[118,119] Scarce case series in humans have not shown teratogenicity in all trimesters of pregnancy. Therefore, rhEPO can in any case be, for the fetus safely, be prescribed in the third trimester and probably also in the first two trimesters.[87,101-104,112,115,118,120-122] Also during lactation it can probably safely be given because the rhEPO protein will probably be broken down in the fetus' gut.[87,103] The main risk of using rhEPO for the mother is (worsening) hypertension.[123] Therefore, increase in Ht should not increase more rapid than 5% per 4 weeks or higher than 30-35%.[104,124]

Hypoxia Inducible Factor (HIF)-activators are new medication in the treatment of anemia. However, treatment during conception and pregnancy is discouraged because these are small molecules possibly passing through the placenta, and having direct and indirect effects on physiological developmental processes.[87]

Target value for pregnant CKD patients with or without dialysis is Hb 6.2-6.8 mmol/L and Ht 30-35%.[101,113,114,120,125] During pregnancy a hyporesponsiveness for rhEPO may develop. When the patient was already treated with rhEPO before pregnancy, the dose may need to be increased 50-100% in order to imitate the physiological increase of EPO during pregnancy.[101-103,113,114] Dosages should frequently be evaluated, at least every 2-3 weeks.[104]

4. *What is the cut-off for Hb for treatment with a blood transfusion and which precautions should be taken?*

No RCTs have been performed on this subject.

In CKD blood transfusions should be avoided and in particular in potential kidney transplant candidates because of the risk of allosensitization.[126] Therefore, taken into account complaints, age, cause and rapidity of development of the anemia, and cardiopulmonal problems, a blood transfusion can be considered with a Hb <5.0 in the pregnant CKD patient. The trigger value may be Hb <6.0 mmol/L with expected increased blood loss in case of placenta praevia or caesarean section. Transmission of viruses, e.g. Parvo-B19 and CMV, should be prevented because of risk for fetal morbidity and mortality.

<u>Recommendations</u> (no GRADE)

1. Aim at a ferritin level > 80 mcg/L to 500 mcg/L in pregnant CKD patients with intact renal function (eGFR > 60; stage G1-2) and anemia.

2. Aim at a ferritin level > 200 mcg/L to 500 mcg/L in pregnant CKD patients with impaired renal function (eGFR < 60; stage G3-5ND) and anemia.

3. In principle, do not prescribe iron in pregnant CKD patients with ferritin level > 500 mcg/L and/or TSAT > 30%.

4. Aim at a TSAT between 30% and 50% and a ferritin level > 300 mcg/L to 800 mcg/L in pregnant dialysis patients with anemia.

5. Start pregnant CKD patients with iron-deficiency anemia on oral iron supplements; aim at a ferritin level > 80 mcg/L.

6. Consider starting pregnant CKD patients with even mild iron deficiency on such supplements even if their Hb is normal.

7. In the second and third trimesters, switch to parenteral iron supplements if the target levels cannot be achieved with oral agents and regularly check Hb and iron levels.

8. If pregnant CKD patients must be given parenteral iron supplements, prescribe one of the modern, stable iron supplements such as iron carboxymaltose (Ferinject) with a maximum dose of 1000 mg (up to 15 mg/kg/dose) or iron isomaltoside (Monofer/Diafer) in doses of up to 1000 to 2000 mg (maximum 20 mg/kg/dose).

9. If one of the older, less stable iron supplements such as iron sucrose (Venofer) is selected for pregnant hemodialysis patients, give a low dose of up to 62.5 to 100 mg during dialysis because of the possibility of fetal iron accumulation.

10. Aim for an Hb of 6.2 to 6.8 mmol/L (corresponding with an Ht of 30 to 35%) in pregnant CKD patients (with or without dialysis), since the obstetrical outcomes are proven to be better if Hb > 6.2 mmol/L.

11. Exclude iron, folic acid, and vitamin B12 deficiencies and adequately treat any deficiencies before considering prescribing rhEPO.

12. Consider starting pregnant CKD patients with (renal) anemia (Hb < 6.2 mmol/L) on rhEPO. When doing so, always weigh the potential benefits for the well-being of the mother and the obstetrical outcome against the potential risks of vasoconstriction with aggravation of hypertension, particularly if Hb rises quickly.

13. If a rapid increase in Hb is desired, for example because labor is approaching, consider leukocyte-depleted, Parvovirus B19-free, and cEK-compatible erythrocyte transfusions as a

good alternative to rhEPO. Because of the leukocyte depletion, these transfusions are CMV-free too.

14. Because of EPO resistance during pregnancy, consider increasing the rhEPO dose for pregnant patients who already used rhEPO before pregnancy by 50-100% to achieve and/or maintain the Hb target value.
    Regularly review the rhEPO dose in the light of the patient's Hb. For most patients, a maximum dose of twice the normal starting dose can be used as a safe dose.

15. For pregnant CKD patients, perioperative transfusions should be given if Hb < 4.5 mmol/L and considered if Hb < 5.0 mmol/L in a stable situation. However, consider transfusions if Hb < 6.0 mmol/L and major blood loss is expected (e.g. around birth) since the obstetrical outcomes are better if Hb > 6.2 mmol/L.

16. Consider blood transfusions for pregnant dialysis patients who are classified as ASA 3 due to the dialysis if their Hb < 6.0 mmol/L and it cannot be expected that Hb can be corrected within a few weeks with parenteral iron supplements and/or rhEPO. Always give blood transfusions if Hb < 6.0 mmol/L and major blood loss is expected (e.g. around birth).

17. When considering the need for transfusions, take into account the risk of allosensitization in view of organ transplantation in the near or far future. Discuss the potential consequences of blood transfusions.

2.4 New onset nephrological problems during pregnancy

Questions

1. How should diagnostics and treatment be of new nephrological problems (proteinuria/ nephrotic syndrome/ thrombotic microangiopathy (TMA)) in the trimester of pregnancy?
2. How can be distinguished between PE and a primary nephrological problem, and are markers like sFlt1:PIGF ratio helpful?
3. When does an indication for kidney biopsy exist?

Background

New kidney diseases may emerge rarely during pregnancy. When proteinuria is new, it is important to distinguish between PE and a primary kidney problem.

Search and select, Results, Conclusion

Not applicable. The guideline working group did not perform a systematic literature search and analysis, since the working group found the topic not suitable for a literature overview.

Rationale

1. *Diagnostics and treatment of new nephrological problems (proteinuria /nephrotic syndrome/ TMA)*

The glomerular filtration rate increases in the first half of pregnancy because of hemodynamic changes. This physiological hyperfiltration may lead to limited proteinuria (<300 mg/24h) without being caused by kidney disease.[127] Pregnant women who show a decrease instead of increase in eGFR or those who have more proteinuria than expected, should be investigated for the cause. The risk for acute kidney injury (AKI) is higher in patients with preexistent CKD and the incidence is highest in the 3rd trimester. Most often it is caused by hypertensive complications of pregnancy (PE, HELLP syndrome). AKI emerges in 1% of patients with PE and 7-15% with HELLP.[128] On the other hand, in the 1st trimester AKI is most often caused by infections.[129,130]

TMA may be a special cause of AKI, where the pregnancy is the precipitating factor for a latent present problem (e.g. atypical hemolytic uremic syndrome (aHUS) or thrombotic thrombocytopenic purpura (TTP). A French study showed that aHUS presented in 20% of patients during pregnancy or direct after birth.[131] An important differentiating factor for placental and non-placental causes of TMA is the severeness of hemolysis.[130] Hemolysis is severe in aHUS and TTP, while it is most often subtle in HELLP. On the other hand, HELLP is characterized by high serum liver enzymes.[132]

ADAMTS13 activity is necessary to rule out TTP. In placental causes of TMA birth is curative, while it is not in non-placental causes.[130]
The diagnostics of AKI should include investigation of prerenal and postrenal causes. Immunological work-up (including ANCA, anti-dsDNA, anti-GBM, anti-PLA2R) and selectivity index in nephrotic range proteinuria may be useful. High selective proteinuria is associated with steroid sensitive nephrotic syndrome in pregnant women and does not suit the diagnosis of PE.[133]
The treatment of AKI during pregnancy is supportive. Renal and placental perfusion should be guaranteed. More specific treatment is depending on the underlying cause. Pregnant women with symptomatic urolithiasis should primarily receive conservative treatment. In case of urolithiasis that does not respond sufficiently to conservative treatment and/or renal insufficiency with hydronephrosis and especially in the case of (suspected) urinary tract infection, be reluctant to apply retrograde urological interventions in the urinary tract due to the risks of inducing contractions and urosepsis.[134,135] The multidisciplinary team of maternal-fetal medicine specialist, nephrologist, and urologist should weigh the advantages and disadvantages of a more definitive solution in the form of ureterorenoscopy with or without a Double J catheter and a more temporary solution in the form of a nephrostomy catheter.

2. *How can be distinguished between PE and a primary nephrological problem, and are markers like sFlt1:PIGF ratio helpful?*

In the majority of patients with hypertension, proteinuria and/or kidney function decline after 20 weeks of pregnancy, the cause will be PE, although hard to distinguish from primary kidney disease. Furthermore, PE may be a complication of a primary kidney disease.
Placental Growth Factor (PIGF) is a VEGF-related molecule that is highly expressed by trophoblasts in the placenta. During healthy pregnancy the plasma concentration of PIGF increases from <50 pg/mL to >500 pg/mL after 30 weeks amenorrhea.[136] A substantial part of patients with PE has an insufficient increase of PIGF preceding PE. Soluble fms-like tyrosine kinase-1 (sFlt1) is a soluble type VEGR receptor and behaves like an anti-angiogenic protein that binds VEGF and PIGF. This leads to a high sFlt1-PIGF ratio, characteristic for PE. An increased sFLt1 is associated with a decreased circulating VEGF and PIGF, which leads to endothelium and podocyte dysfunction. In the same study administration of sFlt1 to pregnant rats lead to hypertension, proteinuria and glomerular endotheliosis.[137] However, a review/meta-analysis of 34 studies concluded that both markers, when decreased for 30 weeks, do not predict PE later in pregnancy.[138] On the other hand, the sFlt1-PIGF ratio was shown to have a high negative predictive value and thus could help to rule out PE.[139,140] It is

unclear whether the diagnostic value of the ratio decreases with lower renal function, but indeed PIGF is cleared by the kidneys and therefore higher serum values harder to interpret.[141,142]

3. *When does an indication for kidney biopsy exist?*

It must be determined whether the result of the kidney biopsy will lead to a change of treatment and only be performed if so.[143] The most important indications for a kidney biopsy during pregnancy are restricted to the following indications:

-severe nephrotic syndrome in 1st or 2nd trimester,

-nephrotic syndrome with progressive kidney function decline in all trimester,

-suspicion of glomerulonephritis in patients with progressive kidney function decline with glomerular erythrocyturia,

-suspicion of acute rejection in kidney transplant recipients.

After birth persistent non-nephrotic range proteinuria without (progressive) renal insufficiency is only pathological when it persists after 6 months after birth.

The risk for complications of the biopsy is low when performed with ultrasound and thus relatively safe.[143-145]

Recommendations

2.4.1 Proteinuria / nephrotic syndrome / thrombotic microangiopathy (no GRADE)

1. In pregnant women showing a decline in eGFR and/or proteinuria > 300 mg/day during the first half of their pregnancy, analyze the cause of the renal insufficiency using diagnostic tools that would also be used outside pregnancy (ultrasonography, urine tests, and, if indicated, specific immunological tests or a selectivity index).

2. Pregnant women with symptomatic urolithiasis should primarily receive conservative treatment. Be reluctant to apply retrograde urological interventions in the urinary tract due to the risks of inducing preterm labor and urosepsis.

3. Consider thrombotic microangiopathy that is not part of HELLP for women with acute kidney injury and haemolysis during pregnancy and after birth.

2.4.2 Differentiation between preeclampsia and kidney disease (no GRADE)

1. Do not use the sFlt1/PIGF ratio in daily practice to distinguish between preeclampsia and (underlying) primary kidney disease since the available data is not yet sufficiently reliable.

2.4.3 Indication of kidney biopsy

1. Do not refrain from a kidney biopsy in pregnancy if there is a strict indication to do so since the risks of a kidney biopsy under ultrasonographic guidance with an automatic biopsy device are the same for pregnant and non-pregnant women.

   The main indications are:

- severe nephrotic syndrome in the first or second trimester,
- nephrotic syndrome with progressive renal failure in all trimesters,
- suspicion of acute glomerulonephritis in patients with progressive renal failure with glomerular erythrocyturia with or without proteinuria,
- suspicion of acute rejection of a kidney transplant.

2. In patients with postpartum persistent non-nephrotic range proteinuria without (progressive) renal failure, only perform a renal biopsy at least 6 months after the birth due to the high likelihood of spontaneous recovery of pregnancy-related glomerular abnormalities in this postpartum period.

Knowledge gaps

How should new nephrological problems (proteinuria/ nephrotic syndrome) be approached in pregnant patients with CKD?

2.5 Dialysis and pregnancy

Questions

How should the treatment for a pregnant patient on dialysis be arranged?

1. What is the preferred modality of dialysis?
2. What is the required dialysis plan and efficiency?
3. Are there special requirements of the dialysis treatment during pregnancy?

Background

Pregnancy outcomes are better in patients after kidney transplantation than in those whom are treated by dialysis, and therefore most times patients are advised to postpone pregnancy after successful transplantation.[146] However, some dialysis patients cannot have a kidney transplant and still want to fulfill their pregnancy wish.

Search and select

In order to formulate recommendations for dialysis patients with a pregnancy or pregnancy wish we performed a systematic literature review to answer the following question: What are the effects of different dialysis schemes and modalities (intensive hemodialysis, overnight hemodialysis, peritoneal dialysis, dialysis modality, dialysis schedule, dialysis dose, dialysis efficiency) compared to standard intermittent hemodialysis on maternal and fetal or neonatal outcomes?

P: pregnant patient on dialysis;

I: intensive hemodialysis, overnight hemodialysis, peritoneal dialysis, dialysis modality, dialysis schedule, dialysis dose, dialysis efficiency;

C: standard intermittent hemodialysis (three times a week);

O: pregnancy outcome, pregnancy complication, live birth, perinatal mortality, asphyxia, Apgar < 7 5min, gestational age, birth weight, intrauterine growth restriction, mode of delivery, polyhydramnios, preeclampsia/HELLP.

Description of studies

The databases Medline (via OVID) and Embase (via Embase.com)) were searched with relevant search terms from 1st of January 1998 until 15th of March 2019. The systematic literature search resulted in 8526 hits. Studies were selected based on the following criteria: original studies (randomized controlled trials or observational studies) or systematic reviews that compared the maternal and fetal or neonatal outcomes in pregnant women with end stage kidney disease undergoing standard intermittent hemodialysis to another dialysis modality or schedule (intensive

hemodialysis, overnight hemodialysis, peritoneal dialysis, dialysis modality, dialysis schedule, dialysis dose, dialysis efficiency).
71 studies were initially selected based on title and abstract screening. After reading the full text, all studies were excluded, and no studies were included that fulfilled all the inclusion criteria.

Results and Conclusion
Not applicable. No studies were found that answered the search question.

Rationale
No studies were found that answered the search question. The working group identified 3 studies that guided the recommendations.[147-149]

1. In a meta-analysis by Piccoli et al.[147] (2016) all studies published between 1-1-2000 and 31-12-2014 were analyzed: 26 case-series and 90 case reports. Data of 574 pregnancies in 543 dialysis patients (523 on hemodialysis and 51 on peritoneal dialysis (PD)) were analyzed. Dialysis prescriptions were clearly described for hemodialysis but not for PD. Fetal and maternal outcomes were reported. Prematurity was frequent with a median gestational age of 33 weeks (range 26-39). Abortions (<24 weeks) as well as fetal and neonatal mortality could not reliably be extracted due to heterogeneity of the reports. Fetal growth restriction (FGR) was reported in 32% of the babies in only 6 studies that reported on this subject. Congenital defects were seen in ~2% which is similar to the 1-5% in the general population. Maternal mortality was reported in 7 women during 10-year follow-up. More dialysis hours and more dialysis sessions per week tended to better outcomes. Number of dialysis sessions per week was significantly inversely correlated with the risk for prematurity and FGR. A significant higher prevalence of FGR was seen in PD (66.7%) vs HD (31%).
2. Saliem et al.[148] (2016) reported a retrospective cohort study in the USA that concluded a lower risk of pregnancy complications in kidney transplant recipients (KTR) than in dialysis patients. Mothers with end-stage renal disease between 1998 and 2011 were identified: n=264 babies in KTR and n=267 babies in dialysis patients. In the latter diabetes gravidarum and hypertension were more common. The following pregnancy complications were significantly more often reported in dialysis than in KTR: solution placentae (6.7 vs 0%), need for blood transfusion (27 vs 5.7%), postpartum infection (4.9 vs 0%), intra-uterine fetal death (7.1 vs 0%), FGR (33 vs 15%). Incidence of preeclampsia was similar among groups (25 vs 27%). The Dutch guideline working group comments on this study that more pregnancies in dialysis patients were reported than in KTR which suggests a high number of unplanned pregnancies that could lead to worse outcomes.

3. Normand et al.[149] (2018) reported on a retrospective multicenter study in France. They conclude on high risks of pregnancy complications and poor fetal outcomes, despite intensive hemodialysis prescriptions. N=100 pregnancies were reported in n=84 women undergoing hemodialysis in n=41 hemodialysis centers. In 89.4% women had daily dialysis sessions started at a mean gestational age of 13.1±6.5weeks. Dialysis time per week increased from 14.6±4.7 hours during the first trimester to 20.5±3.9hours during the third. Fetal survival was 78% without significant associations with e.g. maternal age or daily dialysis sessions during first or third trimester. Preeclampsia and polyhydramnios were seen in n=18 and n=38 pregnancies respectively. Mean gestational age was 33.2±3.9 weeks and 77% was born preterm (<37 weeks). Mean birth weight was 1719±730g.

In summary, in comparison to KTR, pregnant hemodialysis patients have a higher risk for pregnancy complications, e.g. solution placentae, need for blood transfusion, postpartum infection, intra-uterine fetal death and FGR.[148] PD patients have a higher risk for FGR (67%) versus hemodialysis patients (31%)[147] An inverse linear correlation was reported between number of hemodialysis sessions per week / hemodialysis hours per week and risk for prematurity / FGR.[147] Despite intensive hemodialysis of at least 20 hours per week, mean gestational age is 33 weeks with a high risk for FGR / low birth weight in 32-42% of pregnancies and fetal mortality in 18-22%.[147,149]

Planning a pregnancy

In dialysis patients, there is an increased risk for preeclampsia (19-45%).[147,149,150] Pregnant dialysis patients have an increased risk for blood transfusion despite treatment with rhEPO. A hemoglobin <6.8 mmol/L has been associated with prematurity (gestational age <37 weeks).[106,151] Pregnant hemodialysis patients have an increased risk for fetal mortality (including spontaneous and induces abortions, fetal mortality >24 weeks, neonatal mortality). Chance for a live-born baby is 78-83%,[3,149,152] with an increased risk for prematurity (80% born <37 weeks).[147] FGR (<10th percentile) was reported in 32-44% with a median birth weight of 1700-1800g.[147,149] Risk for congenital defects is not increased in comparison to the general population.[147]

Dialysis modality

There is scarce literature on pregnancy in PD patients.[153,154] Physiological changes during pregnancy might impair the possibility for PD. Furthermore, clearance during PD might be insufficient during pregnancy, especially when residual kidney function is absent. Therefore, hemodialysis seems to be the preferable treatment.

Dialysis intensity and efficiency

A meta-analysis of 574 pregnancies in 543 hemodialysis patients concluded that a higher number of dialysis hours per week was associated with a decreased risk for (severe) prematurity and FGR.[147] Preferably, during pregnancy, the dialysis intensity should be increased to > 36 hours/week with the help of, for example, frequent night dialysis. Alternatively, with low evidence level, some studies refer to the use of urea. Improved pregnancy outcomes were reported with a maternal urea level of 12.5-17.5 mmol/L.[155,156] Use of Kt/V has not been validated for dialysis efficiency during pregnancy.

Timing of initiations of dialysis in pregnancy

In patients with preexisting severe CKD or severe decrease in kidney function during pregnancy, intensive hemodialysis might be started when maternal urea concentration is 17.5 mmol/L despite adequate protein intake restriction. However, course of kidney function, fluid balance, biochemical parameters, blood pressure and uremic symptoms should be considered. Pregnant patients with CKD should therefore be treated in a medical center with experience in the treatment of pregnancy during hemodialysis.

Practical aspects during intensive hemodialysis

A weekly evaluation of the ultrafiltration need is necessary since the mother's weight will increase. In the second trimester the woman's weight increases 300g per week and 300-500 g in the third trimester. Intradialytic hypotension should be avoided.[3,157] Nutritional status should frequently be evaluated by a dietician.[114] Expert consensus is to only restrict sodium and fluid intake, and to aim for a protein intake of 1.5-1.8 g/ideal weight in kg/day; ideal weight = weight with BMI 22 kg/m^2). Biochemical parameters should be evaluated every 2 weeks. Water-soluble vitamins should be increased to double dose. Folic acid should be taken in a dose of 5mg already from preconceptional period.

<u>Recommendations </u>(no GRADE)

1. In hemodialysis patients who want to become pregnant, consider whether a kidney transplant is possible and whether pregnancy can be postponed until one year after transplantation since the pregnancy outcomes after kidney transplantation are significantly better than during hemodialysis.

2. Preferably treat pregnant dialysis patients with intensive hemodialysis instead of peritoneal dialysis.

3. Refer pregnant dialysis patients to a centre that has a multidisciplinary team experienced in treating this rare patient group.

4. Depending on their residual renal function, intensify the hemodialysis schedule of pregnant patients to at least 20 hours/week so that the maternal urea level will always be lower than 17.5 mmol/L from the second trimester onwards.

5. Consider treating hemodialysis patients without residual renal function during pregnancy with high-intensity hemodialysis, preferably with a dialysis intensity of > 36 hours/week with the help of, for example, frequent night dialysis.

6. Do *not* use Kt/V measurements to assess the efficiency of high-intensity hemodialysis of pregnant patients.

7. Consider starting intensive hemodialysis for pregnant women with severe kidney failure who are not on dialysis yet, if – despite an adequate low-protein diet (if necessary, supplemented with amino acids) – the maternal urea level cannot be maintained below 17.5 mmol/L.

8. Regularly conduct fetal ultrasound (growth and doppler) throughout the pregnancy given the high fetal morbidity and mortality.

9. Pregnant dialysis patients treated with intensive hemodialysis should have a daily protein intake of 1.5 to 1.8 g/kg ideal weight/day.

10. Double the dosages of water-soluble vitamins for pregnant patients treated with intensive hemodialysis and start with deliberately high doses of folic acid (5 mg/day) even before conception because these substances will be removed in higher quantities through high-intensity hemodialysis.

11. Have a dietician frequently evaluate the nutritional status of pregnant dialysis patients since prolonged treatment with high-intensity hemodialysis significantly affects the patient's fluid balance and nutritional status.

Knowledge gaps

- How should care for pregnant dialysis patients be provided?
- Is PD inferior to (intensive) hemodialysis in the pregnant population?
- What is the optimal strategy for target urea levels and number of dialysis hours per week in the pregnant population and is this different in the different trimester?

2.6 Immunosuppressive therapy

<u>Questions</u>

10.1 Which immunosuppressive medication used in patients with CKD are allowed in the various trimesters of pregnancy?

10.2 How should dosages of the immunosuppressive medication be adjusted in the various trimesters?

10.3 Which biologicals with nephrological indication (alemtuzumab, belatacept, eculizumab, rATG, rituximab, everolimus and other mTOR inhibitors) can safely be prescribed in the various trimesters?

<u>Background</u>

Medication used by women with CKD and planning a pregnancy should be evaluated for teratogenicity, increased risk for abortion or increased risk for complications in the second or third trimester. In particular, immunosuppressive medication use should be evaluated preconceptionally, also to evaluate the course of underlying disease or kidney transplant function.

<u>Search and select, Results, Conclusion</u>

The guideline working group did not perform a systematic literature search and analysis, since the working group found the topic not suitable for a literature review. The European League Against Rheumatism (EULAR) guideline and the British Society of Rheumatology (BSR) guideline were used as starting point. When these guidelines had no position on particular immunosuppressive medication, the Dutch guideline working group performed a literature search on that medication.

<u>Rationale</u>

Table 2 presents a summary of immunosuppressive medication used in CKD and its periconceptional advise, teratogenicity, pregnancy advice, lactational advice and paternal advice.[98,158-171]

Dosage of immunosuppressive medication in the various trimesters

Immunosuppressive medication should have the lowest effective dosage during pregnancy.[163] Ciclosporin and tacrolimus can decrease in serum concentration from the first trimester on but the unbound part of tacrolimus increases during pregnancy whereby with low trough levels the unbound part still can be sufficient.[158,170,172]

Effect of maternal use of immunosuppressive medication on the neonate

Studies on the long-term effects of immunosuppressive medication used by the mother during pregnancy on the child are scarce. When biologicals are used after 22 weeks gestational age, vaccinations with a live attenuated vaccine are discouraged.[163] Almost all biologicals can be used during breastfeeding period since they reach the child in very low concentrations; when they reach the child, the child's low gastric pH will break down the biological.[169]

Recommendations (no GRADE)

1. Refer CKD patients with a desire to have children for preconception counselling in a university hospital. A medication review and, if necessary, changing (teratogenic) medication must be part of the counselling.

2. If necessary, modify immunosuppression depending on the type of agent at least 3 to 6 months before any conception attempt to evaluate their effectiveness and minimize the risk of teratogenicity (Table 2).

3. Only consider prescribing biopharmaceuticals before conception and during pregnancy if no other safe and effective treatment of a maternal disease is available and only in a centre experienced in the treatment of pregnant CKD patients using immunosuppression. For most biopharmaceuticals, there is little or no data on potential adverse fetal outcomes due to preconceptional or perinatal use and no data at all about safety when used by the male partner. For the latest recommendations, please refer to the concept guidelines on biopharmaceuticals and pregnancy. Almost all biopharmaceuticals can be taken without neonatal risk during breastfeeding.

4. After maternal exposure to immunosuppressants, do not hesitate to conduct neonatal blood tests with leukocyte differentiation and be aware of potential neutropenia because studies of the long-term effects on the child's health after exposure to immunosuppressants during pregnancy are scarce and of low quality.

Knowledge gaps

Which biologicals can be safely used in pregnant women with CKD?

2.7 Anticoagulant therapy

Question

How should pregnant CKD patients with an indication for anticoagulation be treated?

Background

The combination of pregnancy, CKD and indication for anticoagulation can diversely be treated, dependent of underlying diseases and risk factors for thrombosis and bleeding.[173]

Search and select, Results, Conclusion

Not applicable. The guideline working group did not perform a systematic literature search and analysis, since the working group found the topic not suitable for a literature overview.

Rationale

Type of anticoagulation (low molecular weight heparin (LMWH) or vitamin K antagonists (VKA))

When there is an indication for therapeutic anticoagulation, LMWH are most often preferred because of low risk of maternal bleeding 1.5% antepartum and 2% postpartum and do not pass the placenta.[174-176] VKA pass the placenta for approximately 20% in perfusion experiments.[177] Furthermore, during use of VKA, risk of fetal bleeding including intracranial bleeding during labor and 6% risk of embryopathy when used during first trimester.

Anti-Xa-levels

Evaluation of underdosing with reduced dose LMWH might be important in pregnant CKD patients. In pregnancy without CKD the half-life of LMWH decreases 30% because of the increased glomerular filtration rate, but in CKD the hyperfiltration decreases.[176] Also, evaluation of overdosing with anti-Xa-levels can be useful in (pregnant) CKD patients. However, even with full dose based on body weight instead of decreased dose by eGFR, subtherapeutic levels can be measured.[178,179] Thus, the working group argues to not dose higher than full dose.

DOAC

Currently, there is a contraindication for use of DOAC during pregnancy. A higher percentage of miscarriages and congenital defects were reported to relate to use of DOAC.[180]

Specific kidney diseases: glomerular kidney disease

Risk for venous thromboembolic events (VTE) is highest in patients with nephrotic syndrome due to membranous nephropathy followed by minimal change disease/FSGS with a hazard ratio (HR) of 22 and 7.8 respectively, even when adjusted for sex, serum albumin and proteinuria (HR 10.8 and 5.9 respectively). Furthermore, the level of proteinuria (mainly >3.5g/24h) and serum albumin (mainly <29 g/L) are predictors for the VTE risk.[181] Pregnancy and postpartum period (6-12 weeks) are further risk factors.[182,183]
Patients with still active or during pregnancy newly diagnosed glomerular disease might be treated with prophylactic or even therapeutic LMWH during the full pregnancy or when serum albumin decreases <25 g/L due to progression of nephrotic syndrome.[181,184] When to start and in prophylactic or therapeutic dose should be determined with all risk factors (including medical history of VTE, immobilization, obesity, start treatment with glucocorticoids) by a multidisciplinary team consisting of a maternal-fetal medicine specialist, nephrologist, and, if necessary, a coagulation specialist.

Specific kidney diseases: lupus nephritis or SLE with antiphospholipid antibodies
Pregnant patients with an increased risk for VTE due to lupus nephritis or SLE in combination with positive antiphospholipid antibodies have an indication for acetylsalicylic acid (80mg) during full pregnancy for prevention of arterial thrombosis and decreased risk for preeclampsia.[185,186] In case of obstetric antiphospholipid syndrome (with or without CKD) the treatment consists of combination of acetylsalicylic acid (80mg) and prophylactic LMWH.[185-189] Risk for successful pregnancy increases from 20 to 54-80%.[186] From 36 weeks gestational age should individually be determined whether to treat with prophylactic or therapeutic dose of LMWH. However, when there was a prepregnancy indication for therapeutic anticoagulation, therapeutic LMWH should be continued full pregnancy.[185,186]

Postpartum period
In all cases with an indication for prophylactic or therapeutic anticoagulation during pregnancy, this treatment should be continued minimal 6 weeks postpartum.

<u>Recommendations</u> (no GRADE)

1.Pregnant women with CKD and an indication for antithrombotic treatment should preferably be treated by a multidisciplinary team consisting of a maternal-fetal medicine specialist, nephrologist, and, if necessary, a coagulation specialist.

2. The type of anticoagulant (LMWH or VKA), the prophylactic or therapeutic dose, and the duration of treatment strongly depend on the underlying (nephrological) disorder, concomitant co-morbidity, and estimated thrombosis and bleeding risks.

3. During pregnancy, VKA should, in principle, be replaced by therapeutic LMWH due to VKA's teratogenic effects and increased fetal bleeding tendency during labor.
An exception to this rule is pregnant women with a strict indication for VKA such as patients with certain types of low-flow mechanical heart valves.

4. The treatment of pregnant women with CKD who have mechanical valves depends on the thrombosis risk associated with their type of artificial valve and should proceed in close consultation with the attending cardiologist. In this respect, pregnant CKD patients do not differ from healthy pregnant women, and VKA should be given during (part of) the pregnancy in accordance with the multidisciplinary guidelines on antithrombotic treatment, anticoagulants and pregnant women. It is important to know that any change in anticoagulation treatment may result in increased thrombosis risk during pregnancy.

5. Preconceptionally and, at the latest, after the first positive pregnancy test, CKD patients treated with a DOAC should be switched to therapeutic LMWH due to the increased risk of miscarriage or congenital defects.

6. Treat pregnant CKD patients at stage G3-5ND with an indication for therapeutic anticoagulation with an LMWH in accordance with the multidisciplinary guidelines on antithrombotic treatment and the modules for patients with impaired renal function. If reduced doses of LMWH are given, consider monitoring peak anti-Xa levels to prevent both under and overdoses. However, avoid giving doses higher than the normal maximum based on body weight, even if the measured anti-Xa activity should suggest this.

6. For pregnant CKD patients with an indication for anticoagulation, continue the LMWH or VKA even after birth for at least 6 weeks due to the increased thrombosis risk in the postpartum period. Mothers using LMWH or VKA can breastfeed safely.

7. Treat pregnant women with severe nephrotic syndrome and serum albumin < 25 g/L with LMWH in prophylactic or, if necessary, therapeutic doses based on any accessory thrombosis risk factors.

8. Consider giving LMWH in prophylactic doses to pregnant CKD patients with elevated thrombosis risk due to the CKD (especially membranous nephropathy) without severe nephrotic syndrome since the pregnancy should be regarded as an accessory thrombosis risk factor.

9. Treat pregnant women with SLE nephritis and/or antiphospholipid syndrome with low doses of acetylsalicylic acid to reduce the risk of arterial thrombosis and preeclampsia. Combine this drug with prophylactic doses of LMWH if the patient has obstetrical antiphospholipid syndrome.

10. If LMWH is given in therapeutic doses to pregnant patients with a higher-stage CKD (G3b-G5ND), consider monitoring their anti-Xa levels to prevent overdose or accumulation.

11. For dialysis patients, consider giving the daily LMWH when dialysis starts, to avoid having to give additional LMWH to declot the extracorporeal circuit of the dialysis device.

12. For pregnant CKD patients, stop therapeutic LMWH 24 hours before and prophylactic LMWH 10 hours before a planned birth (with or without neuraxial anaesthesia) in accordance with the multidisciplinary guidelines on antithrombotic treatment, anticoagulation, and labor and the sections in the NVA guidelines on neuraxial blocking and anticoagulation applying to pregnant women without CKD.
Do not give LMWH to dialysis patients if a birth (with or without neuraxial anaesthesia) is planned within 24 hours.

Knowledge gaps

How differs treatment with anticoagulants in pregnant patients with CKD from non-CKD?

2.8 Diabetes mellitus

Question

How does treatment of diabetes mellitus differ in pregnant women with CKD compared to pregnant women without CKD?

Background

The combination of diabetes mellitus and pregnancy increases the risk for maternal, fetal and neonatal complications. Furthermore, diabetes mellitus and CKD both increase the cardiovascular risk of the pregnant woman.

Search and select, Results, Conclusion

Not applicable. No systematic literature search was performed, because the Dutch working group found the question was not suitable for a systematic literature overview.

Rationale

Influence of pregnancy on kidney function in diabetic nephropathy

The risk for maternal and fetal complications in pregnant patients with CKD and diabetes mellitus is determined by kidney function and level of proteinuria on the one hand, and glycemic control on the other.[190-192] The risk for worsening kidney function by the pregnancy is increased in patients with eGFR < 60 ml/min and/or proteinuria >1-3 g/24h compared to patients with preserved kidney function and microalbuminuria. However, prospective research with large sample size is lacking and thus it is still not clear whether kidney function decline is worse in pregnant patients with diabetes than in non-pregnant patients with diabetic nephropathy.[190-192]

Influence of diabetes mellitus on obstetric outcomes

In a recent cohort study, obstetric outcomes in 8690 pregnant patients with diabetes mellitus type 1 and 2 were reported.[193] Preterm birth was reported in 42.5% in type 1 and in 23.4% in type 2. Low birth weight was reported in 52.2% and 26.2%, respectively, and admission to the neonatal intensive care unit in 12.7% and 7.8%, respectively. Neonatal mortality and stillborn was 0.7% and 1.0% in type 1, and 1.1% and 1.3% in type 2, respectively. No obstetric outcomes on diabetic kidney disease were described.

Treatment of diabetes mellitus during pregnancy in women with CKD

Sufficient glycemic control before, during and after pregnancy is essential to decrease maternal and fetal complications. Insulin therapy is the treatment of choice during the periconceptional period and pregnancy in both diabetes type 1 and 2. Intensive control is warranted since need for insulin varies during pregnancy (remarkably increase during 2nd and 3rd trimester until 36 weeks). Furthermore, there is a potentially increased insulin sensitivity and extended duration of action of insulin, particularly in patients with higher CKD stages and insulin may accumulate with risk for hypoglycemias in CKD.[194-196]
The limited indication for metformin and glibenclamide is further influenced by the degree of kidney injury.[197] Only one study reported on the effect of DPP4 inhibitor (sitagliptin) during pregnancy (from second trimester on) vs placebo in 263 Chinese women.[198] After 16 weeks follow-up significant better glucose and insulin profile were found in the treatment group. There was no difference in neonatal outcomes between groups. The Dutch guideline working group discourages the use of DPP4 inhibitors, GLP1 agonists and SGLT2 inhibitors in pregnancy until more research on efficacy and safety have been published.

Regulation of diabetes mellitus during corticosteroid treatment for fetal lung maturation.
Strict glucose monitoring and regulation is advised for pregnant patients with CKD who are treated with corticosteroids to promote fetal lung maturation. Pregnant patients with CKD and diabetes should therefore be admitted to the hospital.

Recommendations (no GRADE)
1. Treat pregnant patients with diabetes mellitus and CKD in the same way as pregnant patients with only diabetes mellitus, considering the potentially increased insulin sensitivity and extended duration of action of insulin, particularly in patients with higher CKD stages.

2. Consider prescribing shorter-acting insulin types (e.g. insulin isophane) to pregnant patients with diabetes mellitus and CKD in stage 3b or higher given the extended duration of action of insulin in these patients.

3. Consider more frequent blood sugar checks with a glucose sensor for pregnant patients with diabetes mellitus and CKD of stage 3b or higher.

4. In exceptional cases, treat pregnant patients with type 2 diabetes and CKD with metformin or glibenclamide, considering the dose adjustments and contraindications applying to specific CKD stages.

5. At this time, do not prescribe DPP4 inhibitors, GLP-1 agonists, or SGLT-2 inhibitors during pregnancy, not even to pregnant CKD patients with diabetes.

6. Prescribe corticosteroids to pregnant patients with diabetes mellitus (whether they have CKD or not) to promote fetal lung maturation only in clinical settings due to the need for intensive blood sugar monitoring and frequent insulin adjustments.

Knowledge gaps

How does treatment of diabetes mellitus differ in pregnant women with CKD compared to pregnant women without CKD?

2.9 Follow-up of preeclampsia or new onset kidney disease after delivery

Question

How should the follow-up after birth be for patients with PE or nephrological problems during pregnancy?

Background

The follow-up of patients who had a new nephrological problem during pregnancy, is dependent on the disease.

Search and select, Results, Conclusion

Not applicable. No systematic literature search was performed, because the Dutch working group found the question was not suitable for a systematic literature overview.

Rationale

Patients who had an AKI superposed on PE or HELLP syndrome, have a higher risk for negative renal outcome, in particular when the patient had preexistent CKD. These patients should have follow-up at the outpatient clinic of nephrology because of possible persisting CKD. Similarly, also patients with a nephrotic syndrome, glomerulonephritis or TMA should have this follow-up.

Patients who had PE in at least one pregnancy have higher risk for cardiovascular disease later in life.[199,200] Covella et al.[201] (2019) investigated in a review/meta-analysis the risk for development of CKD or increased albuminuria after PE in more than 110,000 patients from 21 retrospective/observational studies with a minimal follow-up of 4 years, compared to 2.5 million control pregnancies. The relative risk (RR) for development of CKD stage 3 or higher after PE was not statistic significant (RR 2.03, 95%CI 0.58-7.32). RR for development of albuminuria was neither statistic significant (RR 4.31, 95%CI 0.95-19.58). The only significant outcome parameter was development of end-stage kidney disease (RR 6.35, 95%CI 2.75-14.79; absolute risk 0.3 vs 0.06%). The number needed to be in follow-up was calculated n=310 patients after PE to find n=1 patient with end-stage kidney disease in the future. It is not clear how many patients with preexisting CKD were included.

In summary, the working recommends that patients with PE but without AKI should have follow-up at the general practitioner (GP) because of increased life-time risk for cardiovascular events.

Recommendations (no GRADE)

1. Patients with the following postpartum characteristics must be monitored at the nephrology clinic:

- patients with acute renal failure superimposed on preeclampsia or HELLP syndrome whose renal failure does not resolve quickly and completely after the birth;
- patients who develop nephrotic syndrome, glomerulonephritis, thrombotic microangiopathy, or any other primary nephrological disorder during pregnancy.

2. Recommend that these patients be given nephrological and obstetrical preconception counselling before a future pregnancy.

3. Inform patients who have had preeclampsia about their increased lifetime cardiovascular risk and the need for regular follow-ups and, if necessary, treatment of other cardiovascular risk factors.

4. Advise patients who have only had preeclampsia to have their GP conduct life-long follow-ups due to their increased lifetime cardiovascular risk.

Part 3: Obstetric treatment during pregnancy

3.1 Advanced ultrasonography

Question

Should advanced ultrasonography be used in pregnant patients with CKD or pregnant women with a spouse with CKD, and when should it be used?

Background

Advanced ultrasonography is and ultrasonography of the unborn child. Advanced ultrasonography type 1 is performed in pregnant patients with an increased risk for the fetus (more than population risk of 2.5%).[202] Advanced ultrasonography type 2 is performed for a fetus when structural defects are supposed because of earlier investigations. When there is no indication for advanced ultrasonography, then the pregnant woman could be investigated by regular structural ultrasonography.

Search and select

Not applicable. No systematic literature review was performed, because a national list with indications for advanced ultrasonography did already exist.

Results and Conclusion

Not applicable. No systematic literature search was performed, because the Dutch working group found the question was not suitable for a systematic literature search.

Rationale

Development of the fetus' kidneys start in the first trimester. Glomerular filtration starts 9 weeks gestational age, but does not contribute to amniotic fluid until 16 weeks. Fetal urinary production starts having an important contribution to amniotic fluid until 300 mL/kg fetal weight/day. The bladder can first be seen 10-14 weeks gestational age and is 10mL 30 weeks gestational age and 50mL at term.[203]

In the healthy population, in 1 to 4 per 1000 pregnancies a defect on kidneys of urinary tract is found by ultrasound, but in patients with CKD this incidence is probably higher. For example, Congenital Anomalies of the Kidney and Urinary Tract (CAKUT) can be found, but amongst others, also ADPKD which can rarely present in (pre)neonatal period.[203,204]

All prospective parents with CKD due to a structural defect not based on a known genome abnormality and women with CKD who must use teratogenic medication during pregnancy have an indication for advanced ultrasonography.

Recommendations (no GRADE)

1. For any prospective parent (m/f) with CKD, consider whether there is an indication for advanced ultrasonography of the fetus.

2. All prospective parents with CKD due to a structural defect not based on a known genome abnormality and women with CKD who must use teratogenic medication during pregnancy have an indication for advanced ultrasonography of the fetus.

Knowledge gaps

Should advanced ultrasonography be used in patients with CKD and when should it be performed?

3.2 Decreasing preeclampsia risk

Questions

Which interventions are useful to diminish the risk for preeclampsia (PE) in pregnant patients with CKD?

1. Which medication can diminish the risk for PE in patients with CKD?
2. Which dietary measures can diminish the risk for PE in patients with CKD?

Background

The kidneys undergo several physiological changes during pregnancy. Early in pregnancy renal blood flow increases with 70% and the glomerular filtration rate with more than 50%. Besides this hyperfiltration, tubular and endocrine functions of the kidneys change as well. In women with CKD, this physiological adaption has probably been disrupted.[14] Therefore, the risk for hypertension, PE and HELLP syndrome is increased in this population, despite stage of CKD. The risk is also influenced by the grade of kidney damage, preexisting hypertension and/or proteinuria, and the underlying disease.[2] The precise influence of every factor is not clear.

Search and select, Results, Conclusion

Not applicable. No systematic literature search was performed, because the Dutch working group found the question was not suitable for a systematic literature review.

Rationale

Acetylsalicylic acid

In the Dutch guideline 'Hypertensive disorders of pregnancy' has been determined that women with an increased risk for PE has to be treated with acetylsalicylic acid.[37] An increased risk profile has been characterized by a heterogenous diversity of risk factors in literature. Here, the Dutch working group used the NICE guideline (2010), the USPSTF guideline and meta-analysis by Bartsch.[43,205,206] These define women with CKD as high risk without further explanation.

The meta-analysis by Roberge et al.[207] (2017) included 21 RCTs for the effect of acetylsalicylic acid compared to placebo/no treatment. In 12 of these RCTs women were included who had an increased risk of PE because of history of chronic hypertension, cardiovascular and endocrine disorders, pregnancy induced hypertension, fetal growth restriction and/or PE. This meta-analysis gives proof for reduction of the risk of PE with an absolute risk reduction of 7.4% after treatment with acetylsalicylic acid in women with an increased risk profile.

Costs of acetylsalicylic acid are low (5 eurocent per tablet). Side effects are rare with low doses. The effects of acetylsalicylic acid per individual risk factor or a combination thereof, have not been investigated sufficiently. No literature exists about the effect of acetylsalicylic acid compared to placebo/no treatment in women with CKD.

The Dutch working group recommends prescribing prophylactic acetylsalicylic acid to all pregnant women with CKD because of the increased risk of PE. The recommendation is to start prophylactic treatment of acetylsalicylic acid in a dose of 80-150 mg/day from 12 weeks amenorrhea and preferably before finishing the 16th week.[37] Quit the treatment minimally one week before labor or a surgery (caesarian section), or at 36 weeks amenorrhea. Preferably acetylsalicylic acid is taken in the evening because of possible more effect and it was done so in research.

Calcium suppletion

WHO recommends to advice all pregnant women to take 1 gram elementary calcium per day.[208] Women with a lower calcium intake should start with calcium suppletion (500-1000 mg elementary calcium once a day on a sober stomach). These advices are based on a systematic review of 13 RCTs which included 15370 women.[209] Calcium suppletion reduced the risk of PE (13 studies, RR 0.45, 95%CI 0.31-0.65). Calcium suppletion seems to have no effect in women with an adequate calcium intake (4 studies, 5022 women, RR 0.62, 95%CI 0.32-1.20). There was an increased risk of HELLP in the group with calcium suppletion (2 studies, 12901 women, RR 2.67, 95%CI 1.05-6.82), but with a low incidence (16 vs 6 women).

Sodium restriction

Sodium restriction in patients with CKD does probably not lead to improvement of kidney function, but indeed to better blood pressure regulation and decrease of albuminuria, as a surrogate endpoint of loss of kidney function.[210] Therefore, patients with CKD are advised to restrict sodium intake to maximum 2400 mg sodium per day. There is no literature about the effect of sodium restriction on the risk of PE in patients with CKD. On the other hand, a sodium restriction of 1200-2400 mg sodium from 14-36 weeks gestational age is not associated with maternal or fetal complications in healthy pregnancies.[22,25] Thus, if necessary, because of nephrological reasons e.g. nephrotic range proteinuria), a strict sodium restriction may probably be safe in women with CKD.

Protein restriction

There are no RCTs for the effect of protein restriction on pregnancy outcomes including PE in women with CKD. In healthy pregnancies, there seems to be a U-shaped association between protein intake during pregnancy and birth weight.[27] Low and high protein intake have an unfavorable influence on

birth weight. On the other hand, uncontrolled studies by Piccoli et al. show no unfavorable outcomes of protein restricted veganistic /vegetarian diet supplemented with amino acids in pregnant women with CKD and possibly favorable for the growth of the children.[28-30]

<u>Recommendations</u> (no GRADE)

1. Prescribe acetylsalicylic acid to any pregnant woman with CKD because they have a (substantially) higher preeclampsia risk.

2. Start prophylactic acetylsalicylic acid in doses of 80 to 150 mg/day from 12 weeks (after last menstruation) and preferably before the end of the 16th week. Stop the treatment at least one week before the expected natural birth or planned caesarean section, that is, usually at 36 weeks pregnancy, unless the birth is expected earlier.
   Recommend that the woman takes the acetylsalicylic acid in the evening.

3. Recommend that any pregnant woman with CKD use at least 1000 mg/day of elemental calcium (preferably in their food) because this may lower the preeclampsia risk.

4. If insufficient calcium is ingested with food, start elemental calcium suppletion of 500-1000 mg (preferably a combination of calcium and 400-800 IU of colecalciferol), to be taken in one dose on an empty stomach, with the dose depending on the dietary calcium intake.
   Patients should not take calcium supplements at the same time as iron supplements, which should also be taken on an empty stomach.
   Be cautious when prescribing calcium supplements to patients with advanced CKD stages (G4 and higher) and hyperphosphatemia despite adequate dietary measures.

5. Do *not* recommend sodium or protein restriction to prevent preeclampsia in pregnant women with CKD. If necessary, recommend this as part of their CKD treatment.

Knowledge gaps

Which interventions ca decrease the risk of PE in pregnant women with CKD?

3.3 Delivery plan

Question

How should the delivery plan be in a pregnant woman with CKD?

The following subquestions were formulated:

1. Which type of delivery is safe?
2. Which drugs can be given during labor?
3. Should blood umbilical blood be drawn?
4. What should be discussed after birth with the patient?
5. How should kidney function be followed-up?
6. When should the development of the child be followed by a pediatrician?

Background

It is not clear whether there are special delivery issues for a pregnant woman with CKD compared to non-CKD.

Search and select, Results, Conclusion

Not applicable. No systematic literature search was performed, because the Dutch working group found the question was not suitable for a systematic literature review.

Rationale

1. *Which type of delivery is safe?*

Literature is scarce on mode of delivery in pregnant women with CKD. With regard to mode of delivery the primary outcome is solely cesarian section (CS) with a variation of 17-100%, mainly because of variation in practice.[2,147] Pregnancy outcome does not seem to be influenced by mode of delivery. The working group finds, based on (inter)national experience, a vaginal birth should be preferred in women with CKD. CS should only be performed for usual obstetric indications. Timing of delivery is dependent of clinical condition of mother and child. When obstetric complications are absent and kidney disease is stable, spontaneous birth can be awaited.

In women with a kidney transplant, the transplant is located in the right or left retroperitoneal space in the fossa iliaca. When a CS is performed, the aberrant course of the ureter must be noticed. Sometimes it is useful to perform surgery of CS together with urologist or transplant surgeon.

For the rare possibility of a CS in a patient on peritoneal dialysis, it can be considered to perform a transperitoneal CS and temporary switch to hemodialysis, since experience with extraperitoneal CS is limited.[211] The risk of development of peritonitis should be considered.

There is a high chance that the anesthesiologist needs to be consulted in pregnant women with CKD. Therefore, it is recommended to refer every pregnant woman with CKD stage 3b or higher at least beforehand to the preoperative screening.
Neuroaxial techniques can safely be administered in women with CKD independent of severeness of CKD, although eGFR <30 mL/min/1.73m2 in combination with a selective NSAID like acetylsalicylic acid is a relative contraindication.[212] In case of (expected) bleeding because of uremic thrombocytopathy a transfusion with thrombocytes cannot diminish the problem because of the uremic situation. The only options for uremic thrombocytopathy are:

- Blood transfusion: Hb is scavenger for NO and erythrocyte flow lead to movement of thrombocytes to the endothelium which improves the interaction between vessel wall and thrombocytes and thus coagulation.[213]
- Desmopressin: high dose desmopressin causes release of Von Willebrand factor (vWF) from the endothelium which might compensate for the relative adhesion defect of thrombocytes in uremic blood.[214] Furthermore, desmopressin can improve thrombocyte function in patients who use platelet aggregation inhibitors like acetylsalicylic acid.[215] Desmopressin does not pass the placenta and does not arrive in breast milk.[216,217] No RCTs are available on efficacy and safety of desmopressin in pregnant women with congenital bleeding problems, but uncontrolled case series have shown its efficacy.[218,219] When it is administered, the first 24h fluid intake should be restricted to 1500 mL and blood pressure and serum sodium should be regularly checked because of the antidiuretic effect.

After birth, women should be weighted daily for evaluation of volume status and women with CKD have an increased risk of complications through fluid overload or deficiency.

2. Which drugs can be given during labor?

In general, in patients with eGFR >30 mL/min/1.73m2 no adjustments in drugs are necessary. If eGFR is <30 mL/min/1.73m2 it is necessary to evaluate necessity of dose adjustments since most obstetric drugs have a renal clearance. For a number of situations with high prevalence recommendations are described below:

- Initiation of delivery: administration of oxytocin is safe and does not need dose adjustment. Priming of the cervix can be done by Foley catheter or misoprostol vaginally or orally. However, in end-stage kidney disease (eGFR <10 mL/min/1.73m2) a doubling of half-life, maximal plasma concentration and AUC of its active form is described.[220] Therefore, in end-stage kidney disease it is preferred to choose the Foley balloon catheter.

- Corticosteroids: pregnant patients with CKD who have been treated with glucocorticoids longer than 2 weeks in a dose of >7.5 mg prednisone or its equivalents should get a glucocorticoid stress treatment during labor when the treatment was ended <1 year ago or is still active. When there are complications, instable hemodynamics or a superposed infection, the dose increase or duration should be evaluated with the nephrologist.
- Tocolysis: when tocolysis is necessary because of premature contractions nifedipine is preferred in The Netherlands. In patients with CKD the advantage is no dose adjustments are necessary based on kidney function. When acute tocolysis is necessary, the oxytocin receptor antagonist atosiban is preferred instead of beta-receptor agonists.[221] However, there is a lack of pharmacokinetic data in patients with (severe) decreased kidney function. Risks for the fetus must be weighed against the possible risk for the mother.
- Antibiotics: many used antibiotics like benzylpenicillin, feneticillin, amoxicillin and amoxicillin/clavulanic acid need to be dose adjusted based on kidney function. Local guidelines and the Renal Drug Handbook should be consulted.[220]
- Antihypertensive drugs: methyldopa, labetalol and other lipophilic beta-blocking agents, and dihydropyridine calcium antagonists like nifedipine and nicardipine can be used safely.
- Magnesium sulphate: when eGFR is <30 mL/min/1.73m2 there is an increased risk for accumulation of magnesium sulphate and thus intoxication. Its obstetric indication is a severe PE and aim is to decrease the risk of eclamptic insult.[37] With an imminent preterm birth <30 weeks it is also administered for the neuroprotective effect on the child with a decreased risk for development of cerebral paresis. Dose adjustments should be done when eGFR <30 mL/min/1.73m2:
    - Similar loading dose 4 g in 10-30 minutes,
    - Continuation dose 0.5 g/h (instead of 1.0 g/h),
    - Evaluate the patient regularly for signs of intoxication (EKG changes with prolonged PQ interval, broadening of QRS complex, diminished patella reflex),
    - Evaluate regularly serum magnesium levels with a target level 2-3 mmol/L,[37]
    - When overdose has occurred, administer directly 1000 mg calcium in 5 minutes intravenously.
- Fluxus postpartum: most important is to find the cause of bleeding and stop this.
    - In case of uterus atony it is safe to administer oxytocin and carbetocin and no dose adjustment is necessary.
    - For the prostaglandin E2 derivative sulproston risks for complications (hypertension, myocardial ischemia, decrease of diuresis) should be weighed against its advantages.

- The competitive plasminogen inhibitor tranexamic acid can be used but needs dose adjustments from eGFR <50 mL/min/1.73m2.[220] It should not be administered for bleeding in the upper urine tract because of the possibility of clotting.

3. *Should blood be umbilical blood be drawn?*

An indication exists for drawing umbilical blood when the mother used immunosuppressive agents or biological during pregnancy, and results should be evaluated with a pediatrician if applicable.
When there is a risk for a genetic kidney disease in the child that could have consequences in its first years of life, it can be useful to draw blood and keep it for storage at the department of clinical genetics. The indication for direct genetic diagnostics from umbilical blood should before or during pregnancy be discussed with the clinical geneticist.

4. *What should be discussed after birth with the patient?*

After birth a critical review of medication is necessary, including compatibility with lactation. Before discharge from the hospital appointments for check-ups with the nephrologist should be made. The woman should have instructions when to call with emergency symptoms (e.g. from hypertension). Furthermore, anticonception needs to be discussed.

5. *How should kidney function be followed-up?*

Kidney function should be checked at least once <24h postpartum. When there were severe maternal complications (PE, decreased kidney function), it should be checked daily until maternal condition has improved and kidney function has stabilized.

6. *When should the development of the child be followed by a pediatrician?*

In principle the pediatric guidelines are followed. Children born prematurely will always be followed, as well as children with congenital defects.

<u>Recommendations</u> (no GRADE)

1.In principle, aim at a vaginal birth in women with CKD and/or a functioning kidney transplant. Perform a caesarian section in accordance with the applicable guidelines if the obstetrical parameters suggest this.

2.Time the birth based on the clinical parameters. Discuss the advantages and disadvantages of inducing labor in CKD patients after the 38th week due to the increased risks of preeclampsia, loss of renal function, and stillbirth.

3.In the event of obstetrical complications such as fetal growth delay, preeclampsia, or deterioration of the mother's condition and/or kidney function, consider inducing labor early, preferably in consultation with the nephrologist.

4.In patients with CKD stages G4 and above (eGFR < 30 mL/min/1.73m^2), consider one or more of the following measures before or during labor:

- restraint before applying neuroaxial techniques in patients who have stopped taking acetylsalicylic acid less than 5 days before;
- if potentially severe bleeding is expected or occurs due to uremic thrombocytopathy, especially if the patient stopped taking acetylsalicylic acid less than 5 days before:
    - blood transfusions if their Hb drops below 6.2 mmol/L,
    - 0.3 to 0.4 mcg/kg of desmopressin i.v. When desmopressin is given, limit the patient's fluid intake to a maximum of 1500 mL all-in over the first 24 hours after the desmopressin administration to prevent overfilling and hyponatraemia with the associated risk of epileptic seizures. Regularly check the patient's blood pressure and serum sodium levels for the first 24 hours after administration;
- preferably prime the cervix with a Foley balloon catheter because misoprostol may lead to higher plasma levels of its active form;
- if labor must be induced, do so with oxytocin since this does not require adjusting the dose in kidney patients;
- lower the maintenance dose of magnesium sulphate: normal 4 g loading dose in 10 to 30 minutes and 0.5 g/hour maintenance dose. Frequently check the patient for signs of intoxication and regularly monitor their blood magnesium levels;
- any drug given during labor should be checked to verify that it is allowed and whether the dose should be adjusted; do not hesitate to consult with a nephrologist and/or pharmacist.

5.Consider one or more of the following measures if CKD patients have an antepartum haemorrhage:

- focus on finding the cause of the problem and, if possible, solve it; do not hesitate to move the patient to an operating theatre;
- give oxytocin or carbetocin if indicated and independent of renal function;

- be reluctant to give sulprostone (Nalador) because of its serious cardiovascular side effects;
- if necessary, give intravenous tranexamic acid but reduce the dose if eGFR < 50 mL/min/1.73m^2.

6.Apply the usual transfusion limits (4-5-6 rule), also in women with CKD. If a massive transfusion is given, regularly check the patient's potassium levels because women with renal failure run an increased risk of hyperkalaemia.

7.Test a sample of umbilical cord blood if the mother has used immunosuppressants such as azathioprine or biopharmaceuticals during pregnancy. These agents may cause maternal leukopenia and/or thrombocytopenia and are known to cause neonatal bone marrow suppression.

8.Check the renal function of CKD patients daily until the mother's condition is stable and her renal function has stabilized; also ensure adequate monitoring at the maternity ward. Ask the original physician treating the patient's chronic kidney disease (nephrologist, internist, or GP) to continue such checks.

9.Around the date of birth, treat CKD patients who have used glucocorticoids for more than 2 weeks in the past year by following a steroid stress dosing plan according to the local protocol and, if necessary, in consultation with a nephrologist.

Knowledge gaps

Which medical treatment issues during labor should be different in pregnant patients with CKD compared to non-CKD?

References

1. Piccoli GB, Fassio F, Attini R, et al. Pregnancy in CKD: whom should we follow and why? *Nephrol Dial Transplant.* 2012;27(Suppl 3):111-118.
2. Piccoli GB, Cabiddu G, Attini R, et al. Risk of adverse pregnancy outcomes in women with CKD. *J Am Soc Nephrol.* 2015;26(8):2011-2022.
3. Hladunewich M, Hou S, Odutay A, et al. Intensive hemodialysis associates with improved pregnancy outcomes: a Canadian and United States cohort comparison. *J Am Soc Nephrol.* 2014;25(5):1103-1109.
4. Zhang JJ, Ma XX, Hao L, Liu LJ, Lv JC, Zhang H. A systematic review and meta-analysis of outcomes of pregnancy in CKD and CKD outcomes in pregnancy. *Clin J Am Soc Nephrol.* 2015; 10(11):1964-1978.
5. Bramham K, Seed PT, Lightstone L, et al. Diagnostic and predictive biomarkers for pre-eclampsia in patients with established hypertension and chronic kidney disease. *Kidney Int.* 2016;89(4):874-885.
6. Piccoli G, Attini R, Cabiddu G, et al. Maternal-fetal outcomes in pregnant women with glomerulonephritides. Are all glomerulonephritides alike in pregnancy? *J Autoimmun*. 2017; 79: 91-98.
7. He Y, Liu J, Cai Q, et al. The pregnancy outcomes in patients with stage 3-4 chronic kidney disease and the effects of pregnancy in the long-term kidney function. *J Nephrol.* 2018; 31(6):953-960.
8. Shah S, Venkatesan RL, Gupta A, et al. Pregnancy outcomes in women with kidney transplant: meta-analysis and systematic review. *BMC Nephrol.* 2019;20(1):24.
9. Wiles K, Webster P, Seed PT, et al. The impact of chronic kidney disease stages 3-5 on pregnancy outcomes. *Nephrol Dial Transplant.* 2021;36(11):2008-2017.
10. Schunemann HJ, Oxman AD, Brozek J, et al. Rating Quality of Evidence and Strength of Recommendations: Grading quality of evidence and strength of recommendations for diagnostic tests and strategies. *BMJ.* 2008;336(7653):1106.
11. Schünemann HJ, Brożek J, Guyatt G, Oxman A. GRADE handbook for grading quality of evidence and strength of recommendations. Updated October 2013. The GRADE Working Group, 2013. Available from http://gdt.guidelinedevelopment.org/central_prod/_design/client/handbook/handbook.html
12. Rahman FZ, Rahman J, Al-Suleiman SA, Rahman MS. Pregnancy outcome in lupus nephropathy. *Arch Gynecol Obstet.* 2005;271(3):222–226.
13. KDIGO 2012 Clinical Practice Guideline for the Evaluation and Management of Chronic Kidney Disease. *Kidney Int*. 2013;3:1. http://www.kidney-international.org.

14. Williams D, Davison J. Chronic kidney disease in pregnancy. *BMJ*. 2008;336(7637):211-215.
15. Van Eerde AM, Krediet CTP, Rookmaker MB, Van Reekum FE, Knoers NVAM, Lely AT. Pre-pregnancy advice in chronic kidney disease: do not forget genetic counseling. *Kidney Int.* 2016;90(4):905-906.
16. Jesudason S, Tong A. The patient experience of kidney disease and prepregnancy. *Best Practice Research Clinical Obstetrics Gynaecology.* 2019;57:77-88.
17. Vivante A, Hildebrandt F. Exploring the genetic basis of early-onset chronic kidney disease. *Nat Rev Nephrol*. 2016;12(3):133-146.
18. Groopman EE, Rasouly HM, Gharavi AG. Genomic medicine for kidney disease. *Nat Rev Nephro*l. 2018;14(2):83-104.
19. Bach KE, Kelly JT, Palmer SC, Khalesi S, Strippoli GFM, Campbell KL. Healthy Dietary Patterns and Incidence of CKD: A Meta-Analysis of Cohort Studies. *Clin J Am Soc Nephrol.* 2019;14(10):1441-1449.
20. Duley L, Henderson-Smart D, Meher S. Altered dietary salt for preventing pre- eclampsia, and its complications. *Cochrane Database Syst Rev.* 2005;(4):CD005548.
21. Kramer MS, Kakuma R. Energy and protein intake in pregnancy. *Cochrane Database Syst Rev.* 2003;(4):CD000032.
22. Knuist M, Bonsel GJ, Zondervan HA, Treffers PE. Low sodium diet and pregnancy-induced hypertension: a multi-centre randomised controlled trial. *Br J Obstet Gynaecol.* 1998;105(4): 430-434.
23. Franx A, Steegers EA, de Boo T, Thien T, Merkus JM. Sodium-blood pressure interrelationship in pregnancy. *J Hum Hypertens.* 1999;13(3):159-166.
24. Inoue M, Tsuchihashi T, Hasuo, Y, et al. Salt Intake, Home Blood Pressure, and Perinatal Outcome in Pregnant Women. *Circ J.* 2016;80(10):2165-2172.
25. Delemarre FM, van Leest LA, Jongsma HW, Steegers EA. Effect of low-sodium diet on uteroplacental circulation. *J Matern Fetal Med.* 2000;9(4):197-200.
26. McMahon EJ, Campbell KL, Bauer JD, Mudge DW. Altered dietary salt intake for people with chronic kidney disease. *Cochrane Database Syst Rev.* 2015;(2):CD010070.
27. Morisaki N, Nagata C, Yasuo S, et al. Optimal protein intake during pregnancy for reducing the risk of fetal growth restriction: the Japan Environment and Children's Study. *Br J Nutr.* 2018;120(12):1432-1440.
28. Piccoli GB, Attini R, Vasario E, et al. Vegetarian supplemented low-protein diets. A safe option for pregnant CKD patients: report of 12 pregnancies in 11 patients. *Nephrol Dial Transplant.*2011;26(1):196-205.

29. Piccoli GB, Leone F, Attini R, et al. Association of low-protein supplemented diets with fetal growth in pregnant women with CKD. *Clin J Am Soc Nephrol.* 2014;9(5):864-873.
30. Attini R, Leone F, Montersino B, et al. Pregnancy, Proteinuria, Plant-Based Supplemented Diets and Focal Segmental Glomerulosclerosis: A Report on Three Cases and Critical Appraisal of the Literature. *Nutrients*. 2017;9(7):770.
31. Asamiya Y, Otsubo S, Matsuda Y, et al. The importance of low blood urea nitrogen levels in pregnant patients undergoing hemodialysis to optimize birth weight and gestational age. *Kidney Int.* 2009;75(11):1217–1222.
32. Helal I, Fick-Brosnahan G, Reed-Gitomer B, Schrier R. Glomerular hyperfiltration: definitions, mechanisms and clinical implications. *Nat Rev Nephrol*. 2012;8(5):293–300.
33. Piccoli GB, Attini R, Vigotti FN, et al. Is renal hyperfiltration protective in chronic kidney disease-stage 1 pregnancies? A step forward unravelling the mystery of the effect of stage 1 chronic kidney disease on pregnancy outcomes. *Nephrology.* 2015;20(3):201-208.
34. Gumus II, Uz E, Bavbek N. Does glomerular hyperfiltration in pregnancy damage the kidney in women with more parities? *Int Urol Nephrol*. 2009;41(4):927-932.
35. Ku E, Lee B, Wei J, Weir M. Hypertension in CKD: Core Curriculum 2019. *Am J Kidney Dis*. 2019;4(1):120-131.
36. Stevens PE, Levin A. Evaluation and management of chronic kidney disease: synopsis of the kidney disease: improving global outcomes 2012 clinical practice guideline. *Ann Intern Med.* 2013; 158: 825-830.
37. Dutch Association of Obstetrics & Gynecology. Dutch Guideline Hypertensive Disorders of Pregnancy 2011. Available from: https://richtlijnendatabase.nl/richtlijn/hypertensieve_aandoeningen_in_de_zwangerschap/hypertensieve_aandoeningen_-_startpagina.html
38. Dutch Federation of Nephrology. Dutch guideline Chronic Kidney Disease 2018. Available from: https://richtlijnendatabase.nl/richtlijn/chronische_nierschade_cns/startpagina_-_chronische_nierschade_cns.html
39. Klahr S, Levey AS, Beck GJ, et al. The effects of dietary protein restriction and blood pressure control on the progression of chronic renal disease. Modification of Diet in Renal Disease Study Group. *N Engl J Med*. 1994;330(13): 877-884.
40. Wright JT, Williamson JD, Whelton PK, et al. A randomized trial of intensive versus standard blood- pressure control. *N Engl J Med*. 2015;373(22):2103-2116.
41. Magee LA, von Dadelszen P, Rey E, et al. Less tight versus tight control of hypertension in pregnancy. *New Engl J Med*. 2015;372(5):407-417.

42. Nielsen LR, Damm P, Mathisen ER. Improved pregnancy outcome in type 1 diabetic women with microalbuminuria or diabetic nephropathy: effect of intensified antihypertensive therapy? *Diabetes Care.* 2009;32(1):38-44.
43. Webster K, Fishburn S, Maresh M, Findlay SC, Chappell LC. Diagnosis and management of hypertension in pregnancy: summary of updated NICE guidance. *BMJ*. 2019;366:l5119.
44. Churchill D, Beevers GD, Meher S, Rhodes C. Diuretics for preventing preeclampsia. *Cochrane Database Syst Rev*. 2007;2007(1):CD004451.
45. Report of the National High Blood Pressure Education Program Working Group on High Blood Pressure in Pregnancy. *Am J Obstet Gynecol.* 2000;183(1):S1-s22.
46. Sibai BM, Grossman RA, Grossman HG. Effects of diuretics on plasma volume in pregnancies with long-term hypertension. *Am J Obstet Gynecol.* 1984;150(7):831-835.
47. Jim B, Sharma S, Kebede T, Acharya A. Hypertension in pregnancy: a comprehensive update. *Cardiol Rev.* 2010;18(4):178-189.
48. Olesen C, de Vries CS, Thrane N, MacDonald TM, Larsen H, Sorensen HT. Effect of diuretics on fetal growth: A drug effect or confounding by indication? Pooled Danish and Scottish cohort data. *Br J Clin Pharmacol.* 2001;51(2):153-157.
49. Collins R, Yusuf S, Peto R. Overview of randomised trials of diuretics in pregnancy. *Br Med J (Clin Res Ed).* 1985;290(6461):17-23.
50. Wiles K, Chappell L, Clark K, et al. Clinical practice guideline on pregnancy and renal disease. *BMC Nephrol.* 2019;20(1):401.
51. Awadalla M, Patwardhan M, Alsamsam A, Imran N. Management of Liddle Syndrome in Pregnancy: A Case Report and Literature Review. *Case Rep Obstet Gynecol.* 2017;2017:6279460.
52. Caretto A, Primerano L, Novara F, Zuffardi O, Genovese S, Rondinelli M. A Therapeutic Challenge: Liddle's Syndrome Managed with Amiloride during Pregnancy. *Case Rep Obstet Gynecol.* 2014;2014:156250.
53. Hernandez-Diaz S, Werler MM, Walker AM, Mitchell AA. Folic acid antagonists during pregnancy and the risk of birth defects. *N Engl J Med.* 2000;343(22):1608-1614.
54. Liszewski W, Boull C. Lack of evidence for feminization of males exposed to spironolactone in utero: A systematic review. *J Am Acad Dermatol.* 2019;80(4):1147-1148
55. Yakoob MY, Bateman BT, Ho E, et al. The risk of congenital malformations associated with exposure to β-blockers early in pregnancy: a meta-analysis. *Hypertension*. 2013;62(2):375-381.

56. Bergman JEH, Lutke LR, Gans ROB, et al. Beta-blocker use in pregnancy and risk of specific congenital anomalies: a European case-malformed control study. *Drug Saf.* 2018;41(4):415-427.
57. Hoeltzenbein M, Beck E, Fietz AK, et al. Pregnancy Outcome After First Trimester Use of Methyldopa: A Prospective Cohort Study. *Hypertension.* 2017;70(1):201-208.
58. Kahn K, Zamora J, Lamont RF, et al. Safety concerns for the use of calcium channel blockers in pregnancy for the treatment of spontaneous preterm labour and hypertension: a systematic review and meta-regression analysis. *J Matern Fetal Neonatal Med.* 2010;23(9):1030-1038.
59. Magee LA, von Dadelszen P, Singer J, et al. The CHIPS randomized controlled trial (Control of Hypertension in Pregnancy Study): is severe hypertension just an elevated blood pressure? *Hypertension.* 2016;68(5):1153-1159.
60. Diav-Citrin O, Shechtman S, Halberstadt Y, et al. Pregnancy outcome after in utero exposure to angiotensin converting enzyme inhibitors or angiotensin receptor blockers. *Reprod Toxicol.* 2011;31:540–545.
61. Polifka JE. Is there an embryopathy associated with first-trimester exposure to angiotensin-converting enzyme inhibitors and angiotensin receptor antagonists? A critical review of the Evidence. *Birth Defects Res A Clin Mol Teratol*. 2012;94(8):576-598
62. Opperman M, Padberg S, Kayser A, Weber-Schoendorfer C, Schaefer C. Angiotensin-II receptor 1 antagonist fetopathy - risk assessment, critical time period and vena cava thrombosis as a possible new feature. *Br J Clin Pharmacol*. 2013;75(3):822-830.
63. Weitz C, Khouzami V, Maxwell K, Johnson JW. Treatment of hypertension in pregnancy with methyldopa: a randomized double-blind study. *Int J Gynaecol Obstet*. 1987;25:35–40.
64. Sibai BM, Mabie WC, Shamsa F, Villar MA, Anderson GD. A comparison of no medication versus methyldopa or labetalol in chronic hypertension during pregnancy. *Am J Obstet Gynecol*. 1990;162:960–966.
65. Abalos E, Duley L, Steyn DW. Antihypertensive drug therapy for mild to moderate hypertension during pregnancy. *Cochrane Database Syst Rev.* 2014;(2):CD002252.
66. Easterling T, Mundle S, Bracken H, et al. Oral antihypertensive regimens (nifedipine retard, labetalol, and methyldopa) for management of severe hypertension in pregnancy: an open label, randomised controlled trial. *Lancet*. 2019;394(10203):1011-1021.
67. Redman C, Beilin, J Bonnar J. Treatment of Hypertension in Pregnancy With Methyldopa: Blood Pressure Control and Side Effects. *Br J Obstet Gynaecol.* 1977;84(6):419-426.
68. Nayak A, Nachane H. Risk analysis of suicidal ideations and postpartum depression with antenatal alpha methyldopa use. *Asian J Psychiatr*. 2018;38:42-44.

69. Steiner J, Cassar J, Mashiter K, Dawes I, Russel Fraser T, Breckenridge A. Effects of methyldopa on prolactin and growth hormone. *Br Med J.* 1976;1(6019):1186-1188.
70. Hoeltzenbein M, Fietz AK, Kayser A, et al. Pregnancy outcome after first trimester exposure to bisoprolol: an observational cohort study. *J Hypertens*. 2018;36(10):2109-2117.
71. Fitton CA, Steiner MFC, Aucott L, et al. In-utero exposure to antihypertensive medication and neonatal and child health outcomes: a systematic review. *J Hypertens.* 2017;35:2123–2137.
72. Butalia S, Audibert F, Cote AM, et al. Hypertension Canada's 2018 Guidelines for the Management of Hypertension in Pregnancy. *Can J Cardiol.* 2018;34(5):526-531.
73. Weber-Schoendorfer C, Hannemann D, Meister R, et al. The safety of calcium channel blockers during pregnancy: a prospective, multicenter, observational study. *Reprod Toxicol*. 2008;26:24–30.
74. Mito A, Murashima A, Wada Y, et al. Safety of amlodipine in early pregnancy. *J Am Heart Assoc*. 2019:8(15):e012093.
75. Brown MA, Buddle ML, Farrell T, Davis GK. Efficacy and safety of nifedipine tablets for the acute treatment of severe hypertension in pregnancy. *Am J Obstet Gynecol*. 2002;187:1046–1050.
76. Tabacova S, Little R, Tsong Y, Vega A, Kimmel C. Adverse pregnancy outcomes associated with maternal enalapril antihypertensive treatment. *Pharmacoepidemiol Drug Saf.* 2003;12(8):633-646.
77. Sobanski V, Launay D, Depret S, Ducloy-Bouthors AS, Hachulla E. Special considerations in pregnant systemic sclerosis patients. *Expert Rev Clin Immunol.* 2016;12(11);1161-1173.
78. Molnar M, Kalantar-Zadeh K, Lott E, et al. Angiotensin-converting enzyme inhibitor, angiotensin receptor blocker use, and mortality in patients with chronic kidney disease. *J Amer Coll Cardiol.* 2014:63(7):650-658.
79. Pucci M, Sarween N, Knox E, Lipkin G, Martin U. Angiotensin-converting enzyme inhibitors and angiotensin receptor blockers in women of childbearing age: risks versus benefits. *Expert Rev Clin Pharmacol*. 2015;8:221-231.
80. Li DK, Yang C, Andrade S, Tavares V, Ferber JR. Maternal exposure to angiotensin converting enzyme inhibitors in the first trimester and risk of malformations in offspring: a retrospective cohort study. *BMJ.* 2011;343:d5931.
81. Pedrinelli R, Di Bello V, Catapano G, et al. Microalbuminuria is a marker of left ventricular hypertrophy but not hyperinsulinemia in nondiabetic atherosclerotic patients. *Arterioscler Thromb*. 1993;13(6):900-906.

82. Matsushita K, Coresh J, Sang Y, et al. Estimated glomerular filtration rate and albuminuria for prediction of cardiovascular outcomes: a collaborative meta- analysis of individual participant data. *Lancet Diabetes Endocrinol*. 2015;3:514e525.
83. Parving H, Mauer M, Fioretto P, Rossing P, Ritz E. Diabetic nephropathy. M.W. Taal (Ed.), Brenner and Rector: The Kidney. Elsevier, Philadelphia, PA;2012: pp. 1411-1454.
84. Klag MJ, Whelton PK, Randall BL, et al. Blood pressure and end-stage renal disease in men. *N Engl J Med*. 1996;334(1):13-18 .
85. Buter H, Hemmelder M, Navis G, et al. The blunting of the antiproteinuric efficacy of ACE inhibition by high sodium intake can be restored by hydrochlorothiazide. *Nephrol Dial Transplant*. 1998;13:1682–1685.
86. De Borst M, Navis G. Sodium intake, RAAS-blockade and progressive renal disease. *Pharmacological Research.* 2016;107:344–351.
87. Wiles KS, Nelson-Piercy C, Bramham K., Reproductive health and pregnancy in women with chronic kidney disease. *Nat Rev Nephrol.* 2018;14(3):165-184.
88. Macdonald-Wallis C, Lawlor D, Fraser A, et al. Blood pressure change in normotensive, gestational hypertensive, preeclamptic, and essential hypertensive pregnancies. *Hypertension*. 2012;59:1241-1248.
89. Magee L, von Dadelszen P. Prevention and treatment of postpartum hypertension. *Cochrane Database Syst Rev*. 2013;30(4).
90. Abrams S, Schanler R, Lee M, Rechtman D. Greater mortality and morbidity in extremely preterm infants fed a diet containing cow milk protein products. *Breastfeed Med*. 2014;9(6):281-285.
91. Devlin RG, Fleiss PM. Captopril in human blood and breast milk. *J Clin Pharmacol*. 1981;21: 110- 113.
92. Redman C, Kelly J, Cooper W. The excretion of enalapril and enalaprilat in human breast milk. *Eur J Clin Pharmacol.* 1990;38(1):99.
93. American Academy of Pediatrics Committee on Drugs. Transfer of drugs and other chemicals into human milk. *Pediatrics*. 2001;108(3):776-789.
94. Briggs G, Freeman R, Towers C, Forinash A. Drugs in pregnancy and lactation: A Reference Guide to Fetal and Neonatal Risk. 11th ed. Wolters Kluwer, 2017.
95. Podymow T, Joseph G. Preconception and pregnancy management of women with diabetic nephropathy on angiotensin converting enzyme inhibitors. *Clin Nephrol*. 2015;83:73–79.
96. White W. Management of hypertension during lactation. *Hypertension*. 1984;6(3):297–300.
97. Beardmore K, Morris J, Gallery E. Excretion of antihypertensive medication into human breast milk: a systematic review. *Hypertens Pregnancy*. 2002;21:85–95.

98. Drugs and Lactation Database (LactMed). Bethesda (MD): National Library of Medicine (US). Available from: https://www.ncbi.nlm.nih.gov/books/NBK501922

99. Naito T, Kubono N, Deguchi S, et al. Amlodipine passage into breast milk in lactating women with pregnancy-induced hypertension and its estimation of infant risk for breastfeeding. *J Hum Lact*. 2015;31(2):301-306.

100. Morgan J, Kogutt B, Meek C, et al. Pharmacokinetics of amlodipine besylate at delivery and during lactation. *Pregnancy Hypertens*. 2018;11:77-80.

101. Reddy SS, Holley JL. Management of the pregnant chronic dialysis patient. *Adv Chronic Kidney Dis.* 2007;14:146-155.

102. Sienas L, Wong T, Collins R, Smith J. Contemporary uses of erythropoietin in pregnancy: a literature review. *Obstet Gynecol Surv*. 2013;68(8):594-602.

103. Hladunewich MA, Melamed N, Bramham K. Pregnancy across the spectrum of chronic kidney disease. *Kidney Int.* 2016;89:995-1007.

104. Scott LL. Ramin SM, Richey M, Hanson J, Gilstrap 3rd LC. Erythropoietin use in pregnancy: two cases and a review of the literature. *Am J Perinatol*. 1995;12(1):22-24.

105. Breymann C. Iron deficiency anemia in pregnancy. *Semin Hematol*. 2015;52(4):339-347.

106. Milman N, Bergholt T, Byg K-E, Eriksen L, Graudal N. Iron status and iron balance during pregnancy: a critical reappraisal of iron supplementation. *Acta Obstet Gynecol Scand.* 1999;78:749–757.

107. Allen LH. Anemia and iron deficiency: effects on pregnancy outcome. *Am J Clin Nutr.* 2000;71:1280S-1284S.

108. Steer PJ. Maternal hemoglobin concentration and birth weight. *Am J Clin Nutr.* 2000;71:1285–1287s.

109. Breymann C. Iron deficiency and anemia in pregnancy: modern aspects of diagnosis and therapy. *Blood Cells Mol Dis.* 2002;29:506-516.

110. Levy A, Fraser D, Katz M, Mazor M, Sheiner E. Maternal anemia during pregnancy is an independent risk factor for low birthweight and preterm delivery. *Eur J Obstet Gynecol Reprod Biol*. 2005;122:182–186.

111. Scholl TO. Maternal iron status: relation to fetal growth, length of gestation and the neonate's iron endowment. *Nutr Rev.* 2011;69:S23-29.

112. Sanchez-Gonzalez LR, Castro-Melendez SE, Angeles-Torres AC, Castro-Cortina N, Escobar-Valencia A, Quiroga-Garza A. Efficacy and safety of adjuvant recombinant human erytropoietin and ferrous sulfate as treatment for iron deficiency anemia during the third trimester of pregnancy. *Eur J Obstet Gynecol Reprod Biol.* 2016;205:32-36.

113. Piccoli GB, Conijn A, Consiglio V, et al. Pregnancy in dialysis patients: is the evidence strong enough to lead us to change our counseling policy? *Clin J Am Soc Nephrol.* 2010;5(1):62-71.

114. Cabiddu G, Castellino S, Gernone G, et al. Best practices on pregnancy on dialysis: the Italian Study Group on Kidney and Pregnancy. *J Nephrol.* 2015;28(3):279-288.

115. Breymann C, Visca E, Huch R, Huch A. Efficacy and safety of intravenously administered sucrose with and without adjuvant recombinant human erythropoietin for the treatment of resistant iron-deficiency anemia during pregnancy. *Am J Obstet Gynecol.* 2001;184(4):662-667.

116. Froessler B, Collingwood J, Hodyl NA, Dekker G. Intravenous ferric carboxymaltose for anaemia in pregnancy. *BMC Pregnancy Childbirth.* 2014;14:115.

117. Breymann C, Milman N, Mezzacasa A, Bernard R, Dudenhausen J. Ferric carboxymaltose vs. oral iron in the treatment of pregnant women with iron deficiency anemia: an international, open-label, randomized controlled trial (FER-ASAP). *J Perinat Med*. 2017;45(4):443-453

118. Schneider H, Malek A. Lack of permeability of the human placenta for erythropoietin. *J Perinat Med.* 1995;23:71-76.

119. Widness JA, Schmidt RL, Sawyer ST. Erythropoietin transplacental passage – review of animal studies*. J Perinat Med.* 1995;23(1-2):61-70.

120. Hou SH. Pregnancy in women on hemodialysis and peritoneal dialysis. *Baillieres Clin Obstet Gynaecol*. 1994;8(2):481-500.

121. Blowey DL, Warady BA. Neonatal outcome in pregnancies associated with renal replacement therapy. *Adv Ren Replace Ther.* 1998;5(1):45-52.

122. Rudnyk VT. Effectiveness of the recombinant erythropoietin use in anemia of pregnant women. *Pharmacia*. 2018;65;11-16.

123. Agarwal R. Mechanisms and mediators of Hypertension Induced by Erythropoietin and Related Molecules. *Nephrol Dial Transplant.* 2018;33:1690-1698.

124. Maschio G. Keynote Lecture: Erythropoietin and systemic hypertension. *Nephrol Dial Transplant*. 1995;10(Suppl 2):74–79.

125. Giofre F, Pugliese C, Alatai G, Messina A, Tramontana D. Three successive pregnancies in a patient with chronic renal disease progressing from chronic renal dysfunction through to institution of dialysis during pregnancy and then on to maintenance dialysis. *Nephrol Dial Transplant.* 2007;22(4):1236-1240.

126. Ferrandiz I, Congy-Jolivet N, Del Bello A, jiet al. Impact of early blood transfusion after kidney transplantation on the incidence of donor-specific anti-HLA antibodies. *Am J Transplant.* 2016;16(9):2661-2669.

127. Gammill HS, Jeyabalan A. Acute renal failure in pregnancy. *Crit Care Med.* 2005;33(10 Suppl):S372-384.

128. Sibai BM, Ramadan MK. Acute renal failure in pregnancies complicated by hemolysis, elevated liver enzymes, and low platelets. *Am J Obstet Gynecol.* 1993;*168*(6 Pt 1):1682-1690.

129. Fakhouri F, Vercel C, Fremeaux-Bacchi V. Obstetric nephrology: AKI and thrombotic microangiopathies in pregnancy. *Clin J Am Soc Nephrol.* 2012;*7*(12):2100-2106.

130. Jim B, Garovic VD. Acute Kidney Injury in Pregnancy. *Semin Nephrol.* 2017;37(4):378-385.

131. Fakhouri F, Roumenina L, Provot F, et al. (2010). Pregnancy-associated hemolytic uremic syndrome revisited in the era of complement gene mutations. *J Am Soc Nephrol.* 2010;21(5):859-867.

132. Pourrat O, Coudroy R, Pierre F. Differentiation between severe HELLP syndrome and thrombotic microangiopathy, thrombotic thrombocytopenic purpura and other imitators. *Eur J Obstet Gynecol Reprod Biol.* 2015;189*:*68-72.

133. Hamilton P, Myers J, Gillham J, Ayers G, Brown N, Venning, M. Urinary protein selectivity in nephrotic syndrome and pregnancy: resurrection of a biomarker when renal biopsy is contraindicated. *Clin Kidney J.* 2014;7(6):595-598.

134. Ishii H, Aboumarzouk O, Somani B. Current status of ureteroscopy for stone disease in pregnancy. *Urolithiasis*. 2014;42:1–7.

135. Butticè S, Laganà A, Vitale S. Ureteroscopy in pregnant women with complicated colic pain: Is there any risk of premature labor? *Arch Ital Urol Androl*. 2017;89(4):287-292.

136. Krauss T, Pauer HU, Augustin HG. Prospective analysis of placenta growth factor (PlGF) concentrations in the plasma of women with normal pregnancy and pregnancies complicated by preeclampsia. *Hypertens Pregnancy.* 2004;23(1):101-111.

137. Maynard SE, Min JY, Merchan J, et al. Excess placental soluble fms-like tyrosine kinase 1 (sFlt1) may contribute to endothelial dysfunction, hypertension, and proteinuria in preeclampsia. *J Clin Invest.* 2003;111(5):649-658.

138. Kleinrouweler CE, Wiegerinck MM, Ris-Stalpers C, et al. Accuracy of circulating placental growth factor, vascular endothelial growth factor, soluble fms-like tyrosine kinase 1 and soluble endoglin in the prediction of preeclampsia: a systematic review and meta-analysis. BJOG*.* 2012;*119*(7):778-787.

139. Zeisler H, Llurba E, Chantraine F, et al. Predictive Value of the sFlt-1:PlGF Ratio in Women with Suspected Preeclampsia. *N Engl J Med.* 2016;374(1):13-22.
140. Agrawal S, Cerdeira AS, Redman C, Vatish M. Meta-Analysis and Systematic Review to Assess the Role of Soluble FMS-Like Tyrosine Kinase-1 and Placenta Growth Factor Ratio in Prediction of Preeclampsia: The SaPPPhirE Study. *Hypertension.* 2018;71(2):306-316.
141. Rolfo A, Attini R, Nuzzo AM, et al. Chronic kidney disease may be differentially diagnosed from preeclampsia by serum biomarkers. *Kidney Int.* 2013;83(1):177-181.
142. Wiles K, Chappell LC, Lightstone L, Bramham K. (2020). Updates in Diagnosis and Management of Preeclampsia in Women with CKD. *Clin J Am Soc Nephrol*. 2020;15(9);1371-1380.
143. Day C, Hewins P, Hildebrand S, et al. The role of renal biopsy in women with kidney disease identified in pregnancy. *Nephrol Dial Transplant.* 2008;23(1):201-206.
144. Chen HH, Lin HC, Yeh JC, Chen CP. Renal biopsy in pregnancies complicated by undetermined renal disease. *Acta Obstet Gynecol Scand.* 2001;80(10):888- 893.
145. Wide-Swensson D, Strevens H, Willner J. Antepartum percutaneous renal biopsy. *Int J Gynaecol Obstet.* 2007;98(2):88-92.
146. Mohammadi FA, Borg M, Gulyani A, McDonald SP, Jesudason S. (2017). Pregnancy outcomes and impact of pregnancy on graft function in women after kidney transplantation. *Clin Transplant*. 2017 ;31(10):e13089.
147. Piccoli GB, Minelli F, Versino E, et al. Pregnancy in dialysis patients in the new millennium: a systematic review and meta-regression analysis correlating dialysis schedules and pregnancy outcomes. *Nephrol Dial Transplant*. 2016;31(11):1915-1934.
148. Saliem S, Patenaude V, Abenhaim HA. Pregnancy outcomes among renal transplant recipients and patients with end-stage renal disease on dialysis. *J Perinat Med*. 2016;44:321-327.
149. Normand G, Xu X, Panaye M, et al. Pregnancy Outcomes in French Hemodialysis Patients. *Am J Nephrol*. 2018;47(4):219-27.
150. Sachdeva M, Barta V, Thakkar J, Sakhiya V, Miller I. Pregnancy outcomes in women on hemodialysis: a national survey. *Clin Kidney J*. 2017;10(2):276-281.
151. Rahmati S, Azami M, Badfar G, Parizad N, Sayehmiri K. The relationship between maternal anemia during pregnancy with preterm birth: a systematic review and meta-analysis. *J Matern Fetal Neonatal Med*. 2020;33:2679-689.
152. Hou S. Pregnancy in chronic renal insufficiency and end-stage renal disease. *Am J Kidney Dis*. 1999;33(2):235- 52.

153. Okundaye I, Abrinko P, Hou S. Registry of pregnancy in dialysis patients. *Am J Kidney Dis.* 1998;31(5):766-773.

154. Piccoli GB, Cabiddu G, Daidone G, et al. The children of dialysis: live-born babies from on-dialysis mothers in Italy--an epidemiological perspective comparing dialysis, kidney transplantation and the overall population. *Nephrol Dial Transplant*. 2014;29(8):1578-1586.

155. Asamiya Y, Otsubo S, Matsuda Y, et al. The importance of low blood urea nitrogen levels in pregnant patients undergoing hemodialysis to optimize birth weight and gestational age. *Kidney Int*. 2009;75(11):1217-1222.

156. Luders C, Castro MC, Titan SM, et al. Obstetric outcome in pregnant women on long-term dialysis: a case series. *Am J Kidney Dis*. 2010;56(1):77-85.

157. Tangren J, Nadel M, Hladunewich MA. Pregnancy and End-Stage Renal Disease. Blood Purif. 2018;45(1-3):194-200.

158. Kainz A, Harabacz I, Cowlrick IS, Gadgil SD, Hagiwara D. Review of the course and outcome of 100 pregnancies in 84 women treated with tacrolimus. *Transplantation.* 2000;70(12):1718-1721.

159. Petri M. Immunosuppressive drug use in pregnancy. *Autoimmunity*. 2003;36(1):51-56.

160. Huyghe E, Zairi A, Nohra J, Kamar N, Plante P, Rostaing L. Gonadal impact of target of rapamycin inhibitors (sirolimus and everolimus) in male patients: an overview. *Transpl Int.* 2007;20(4):305-311.

161. Kelly R, Arnold L, Richards S, et al. The management of pregnancy in paroxysmal nocturnal haemoglobinuria on long term eculizumab. *Br J Haematol*. 2010;149(3):446-450.

162. Flint J, Panchal S, Hurrell A, et al. BSR and BHPR guideline on prescribing drugs in pregnancy and breastfeeding- Part I: standard and biologic disease modifying anti-rheumatic drugs and corticosteroids. *Rheumatology (Oxford).* 2016;55(9):1693-1697.

163. Gotestam Skorpen C, Hoeltzenbein M, Tincani A, et al. The EULAR points to consider for use of antirheumatic drugs before pregnancy, and during pregnancy and lactation. *Ann Rheum Dis*. 2016;75(5):795-810.

164. Servais A, Devillard N, Fremeaux-Bacchi V, et al. Atypical haemolytic uraemic syndrome and pregnancy: outcome with ongoing eculizumab. *Nephrol Dial Transplant*. 2016;31(12):2122-2130.

165. Ponticelli C, Moroni G. Fetal Toxicity of Immunosuppressive Drugs in Pregnancy. *J Clin Med*. 2018;7(12):552..

166. Teng YKO, Bredewold EOW, Rabelink TJ, et al. An evidence-based approach to pre-pregnancy counselling for patients with systemic lupus erythematosus. *Rheumatology (Oxford).* 2018;57(10):1707-1720.

167. Langer-Gould AM. Pregnancy and Family Planning in Multiple Sclerosis. *Continuum (Minneap Minn).* 2019;25(3):773-792.

168. Sarno L, Tufano A, Maruotti GM, Martinelli P, Balletta MM, Russo D. Eculizumab in pregnancy: a narrative overview. *J Nephrol*. 2019;32(1):17-25.

169. Soh MC, Moretto M. The use of biologics for autoimmune rheumatic diseases in fertility and pregnancy. *Obstet Med*. 2020;13(1):5-13.

170. Le HL, Francke MI, Andrews LM, de Winter BCM, van Gelder T, Hesselink DA. Usage of Tacrolimus and Mycophenolic Acid During Conception, Pregnancy, and Lactation, and Its Implications for Therapeutic Drug Monitoring: A Systematic Critical Review. *Ther Drug Monit*. 2020;42(4);518-531.

171. Perez-Garcia LF, Dolhain R, Vorstenbosch S, et al. The effect of paternal exposure to immunosuppressive drugs on sexual function, reproductive hormones, fertility, pregnancy and offspring outcomes: a systematic review. *Hum Reprod Update*. 2020;26(6):961-1001.

172. Fischer T, Neumayer HH, Fischer R, et al. Effect of pregnancy on long-term kidney function in renal transplant recipients treated with cyclosporine and with azathioprine. *Am J Transplant*. 2005;5(11):2732-2739.

173. Dager WE, Kiser TH. Systemic anticoagulation considerations in chronic kidney disease. *Adv Chronic Kidney Dis.* 2010;17(5): 420-427.

174. Saivin S, Giroux M, Dumas J, et al. Placental transfer of glycosaminoglycans in the human perfused placental cotyledon model. *Eur J Obstet Gynecol Reprod Biol.* 1991;42:221-225.

175. Schneider D, Heilmann L, Harenberg J. Zur Plazentagängigkeit von niedermolekularem Heparin. *Geburtshilfe Frauenheilkd* . 1995;55(2): 93-98.

176. Lu E, Shatzel J.J, DeLoughery TG. The Safety of Low-Molecular-Weight Heparin During and After Pregnancy. *Obstet Gyncecol Surv*. 2017;72(12):721-729.

177. Bajoria R, Sooranna S, Chatterjee R. Effect of lipid composition of cationic SUV liposomes on materno-fetal transfer of warfarin across the perfused human term placenta. *Placenta*. 2013;34:1216-1222.

178. Van Ojik AL, Hemmelder M, Hoogendoorn M, et al. (2016). Anti-Xa activity of therapeutic nadroparin in patients with renal impairment treated according to the Dutch Federation of Nephrology guideline: Comparison with standard dosing in patients with normal renal function. *Pharmaceutisch Weekblad*. 2016;151(46): 19-22.

179. Olie RH, Meertens NEL, Henskens YMC, Ten Cate H. Empirically Reduced Dosages of Tinzaparin in Patients with Moderate-to-Severe Renal Insufficiency Lead to Inadequate Anti-Xa Levels. *Nephron*. 2017;137(2):113-123.

180. Lameijer H, Aalberts JJJ, van Veldhuisen DJ, Meijer K, Pieper PG. Efficacy and safety of direct oral anticoagulants during pregnancy; a systematic literature review. *Thromb Res*. 2018;169: 123-127.

181. Barbour SJ, Greenwald A, Djurdjev O, Levin A., et al. Disease-specific risk of venous thromboembolic events is increased in idiopathic glomerulonephritis. *Kidney Int*. 2012;81(2):190-195.

182. Kamel H, Navi BB, Sriram N, Hovsepian DA, Devereux RB, Elkind M. Risk of a thrombotic event after the 6-week postpartum period. *New Engl J Med*. 2014;370:1307-1315.

183. Bates SM, Middeldorp S, Rodger M, James AH, Greer I. Guidance for the treatment and prevention of obstetric-associated venous thromboembolism. *J Thromb Trombolysis*. 2016;41:92-128.

184. Hladunewich MA, Bramham K, Jim B, Maynard S. Managing glomerular disease in pregnancy. *Nephrol Dial Transplant*. 2017; 32:i48-i56.

185. Andreoli L, Bertsias G.K, Agmon-Levin N, et al. EULAR recommendations for woman's health and the management of family planning, assisted reproduction, pregnancy and menopause in patients with systemic lupus erythematosus and/or antiphospholipid syndrome. *Ann Rheum Dis*. 2017;76:476-485.

186. Limper M, de Leeuw K, Lely AT, et al. Diagnosis and treating antiphospholipid syndrome: consensus paper. *Neth J Med*. 2019;77(3):98-108.

187. Ziakas PD, Pavlou M, Voulgarelis M. Heparin treatment in antiphospholipid syndrome with recurrent pregnancy loss: a systematic review and meta-analysis. *Obstet. Gynecol.* 2010;115(6):1256-1262.

188. Branch DW. What's new in obstetric antiphospholipid syndrome. *Hematology Am Soc Hematol Educ Program*. 2019;2019(10;421–425.

189. Sammaratino LR, Bermas BL, Chakravarty EE, et al. 2020 American College of Rheumatology Guideline for the management of reproductive health in Rheumatic and musculoskeletal diseases. *Arthritis Rheumatol.* 2020;72(4):529-556.

190. Picolli GB, Clari R, Ghiotto S. Type 1 diabetes, diabetic nephropathy, and pregnancy: a systemetic review and meta-study. *Rev Diabet Stud*. 2013;10(1):6-26.

191. Bramham K. Diabetic nephropathy and pregnancy. *Semin Nephrol*. 2017;37(4):362-369

192. Spotti D. Pregnancy in women with diabetes nephropathy. *J Nephrol*. 2019;32:379-388.

193. Murphy HR, Howgate C, O'keefe J, et al. Characteristics and outcome of pregnant women with type 1 or 2 diabetes: a 5-year national population-based cohort study. *Lancet Diabetes Endocrinol*. 2021;9(3):153-164.

194. Scheen AJ. Pharmacokinetic considerations for the treatment of diabetes in patients with chronic kidney disease. *Expert Opin Drug Metab Toxicol*. 2013;9:529-550.

195. Tuttle KR, Bakris GL, Bilous RW, et al. Diabetic kidney disease: a report from an ADA consensus conference. *Diabetes Care*. 2014;37(10):2864–2883.

196. Rahhal M, Gharaibeh N, Rahimi L, smail-Beigi F. Disturbances in Insulin–Glucose Metabolism in Patients With Advanced Renal Disease With and Without Diabetes*. J Clin Endocrinol Metab*. 2019;104:4949–4966.

197. Kidney Disease: Improving Global Outcomes (KDIGO) Blood Pressure Work Group. KDIGO Clinical Practice Guideline for the Management of Blood Pressure in Chronic Kidney Disease. *Kidney Int Suppl.* 2012;2:337–414.

198. Sun X, Zhang Z, Ning H, et al. Sitagliptin down-regulates retinol-binding protein 4 and reduces insulin resistance in gestational diabetes mellitus: a randomized and double-blind trial. *Metab Brain Dis.* 2017;32:773–778.

199. Ahmed R, Dunford J, Mehran R, Robson S, Kunadian V. Preeclampsia and future cardiovascular risk among women: a review. *J Am Coll Cardiol,* 2014;63(18):1815- 1822.

200. McDonald SD, Malinowski A, Zhou Q, Yusuf S, Devereaux PJ. Cardiovascular sequelae of preeclampsia/eclampsia: a systematic review and meta- analyses. *Am Heart J.* 2008;156(5):918-930.

201. Covella B, Vinturache AE, Cabiddu G, et al. A systematic review and meta-analysis indicates long-term risk of chronic and end-stage kidney disease after preeclampsia. *Kidney Int,* 2019;96(3):711-727.

202. Dutch Association for Obstetrics & Gynecology. Leidraad indicatiestelling prenatale diagnostiek. Utrecht, 2019.

203. Rosenblum S, Pal A, Reidy K. Renal development in the fetus and premature infant. *Semin Fetal Neonatal Med.* 2017;22(2):58-66.

204. Yulia A, Winyard P. Management of antenatally detected kidney malformations. *Early Hum Dev*. 2018;126:38-46.

205. LeFevre ML, U.S. Preventive Services Task Force. Low-dose aspirin use for the prevention of morbidity and mortality from preeclampsia: U.S. Preventive Services Task Force recommendation statement. *Ann Intern Med*. 2014;161(11):819-826.

206. Bartsch E, Park AL, Kingdom JC, Ray JG. Risk threshold for starting low dose aspirin in pregnancy to prevent preeclampsia: an opportunity at a low cost. *PLoS One*. 2015;19;10(3):e0116296.

207. Roberge S, Nicolaides K, Demers S, Hyett J, Chaillet N, Bujold E. The role of aspirin dose on the prevention of preeclampsia and fetal growth restriction: systematic review and meta- analysis. *Am J Obstet Gynecol*. 2017;216(2):110-120.e6. 10.1016/j.ajog.2016.09.076. Epub 2016 Sep 15. Review. PubMed PMID: 27640943.

208. WHO recommendation: Calcium supplementation during pregnancy for the prevention of pre-eclampsia and its complications. Geneva: World Health Organization; 2018.

209. Hofmeyr GJ, Manyame S. Calcium supplementation commencing before or early in pregnancy, or food fortification with calcium, for preventing hypertensive disorders of pregnancy. *Cochrane Database Syst Rev*. 2017;9(9):CD011192.

210. Levey AS, Gansevoort RT, Coresh J, et al. Change in Albuminuria and GFR as End Points for Clinical Trials in Early Stages of CKD: A Scientific Workshop Sponsored by the National Kidney Foundation in Collaboration With the US Food and Drug Administration and European Medicines Agency. *Am J Kidney Dis*. 2020;75(1):84-104.

211. Kapoor N, Makanjuola D, Shehata H. Management of women with chronic renal disease in pregnancy. *The Obstetrician & Gynecologist* 2009; 11:185-191.

212. Dutch Assocation of Anesthesiology. Duch Guideline Neuraxis blockade 2014. Available from: https://richtlijnendatabase.nl/richtlijn/neuraxisblokkade_en_antistolling/neuraxisblokkade_en_antistolling_-_startpagina.html

213. Gäckler A, Rohn H, Lisman T, et al. Evaluation of hemostasis in patients with endstage renal disease. *PLoS ONE.* 2019;14:e0212237.

214. Zwaginga J, Ijsseldijk M, Beeser-Visser N, et al. High von Willebrand factor concentration compensates a relative adhesion defect in uremic blood. *Blood*. 1990; 75(7):1498– 508.

215. Andersen L, Hvas A, Hvas C. Effect of Desmopressin on Platelet Dysfunction During Antiplatelet Therapy: A Systematic Review. *Neurocrit Care*. 2021;34(3):1026-1046.

216. Ray JG. DDAVP use during pregnancy: an analysis of its safety for mother and child. *Obstet Gynecol Surv*. 1998;53(7):450–455.

217. Mannucci PM. Desmopressin (DDAVP) in the treatment of bleeding disorders: the first 20 years. *Blood*. 1997;90(7):2515-2521.

218. Huq FA, Kadir RA. Management of pregnancy, labour and delivery in women with inherited bleeding disorders. *Haemophilia*. 2011;17(Suppl 1):20-30.

219. Karanth L, Barua A, Kanagasabai S, Nair NS. Desmopressin acetate (DDAVP) for preventing and treating acute bleeds during pregnancy in women with congenital bleeding disorders. *Cochrane Database Syst Rev*. 2015;(9):CD009824

220. Renal Drug Handbook 5th edition, 2019. Editors: Ashley C and Dunleavy A.

221. Tsatsaris V, Carbonne B, Cabrol D. Atosiban for Preterm Labour. *Drugs*. 2004;64:375-382.

Figure S1: Birth weight and NICU admittance according to prepregnancy CKD stage.

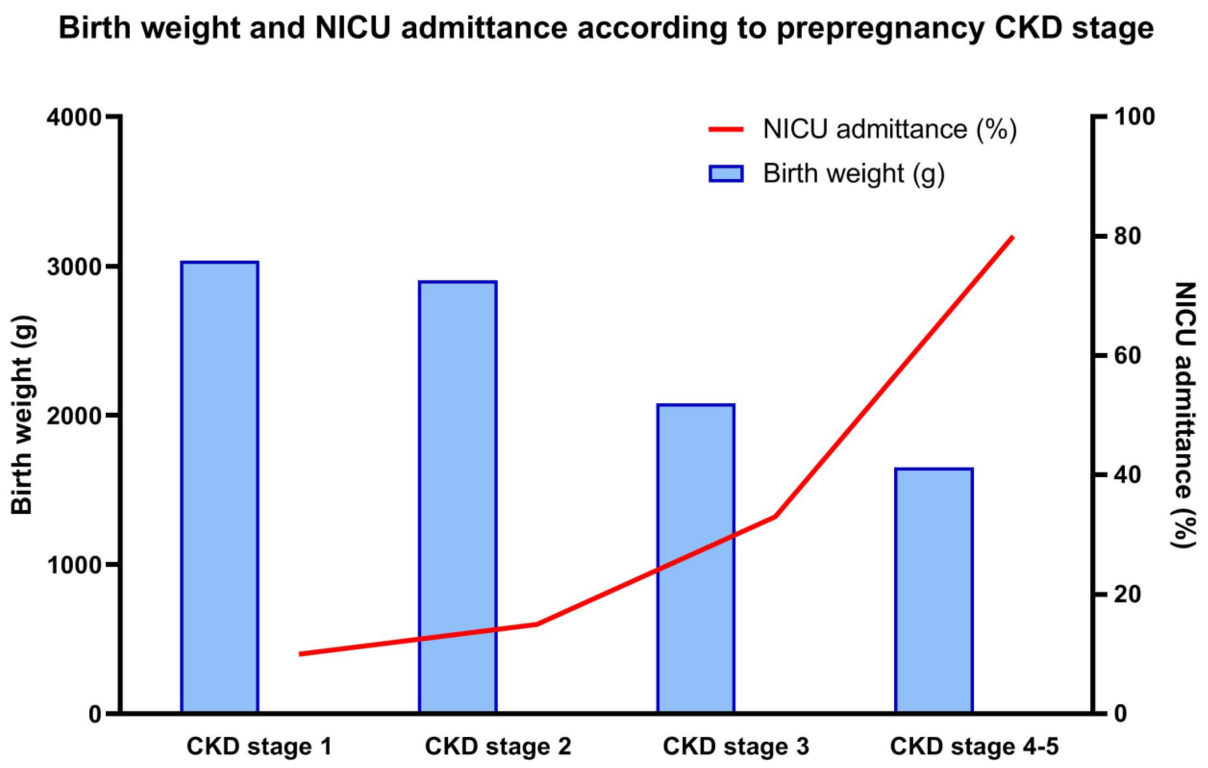

Figure S2: Maternal complications and admittance during pregnancy according to CKD stage.

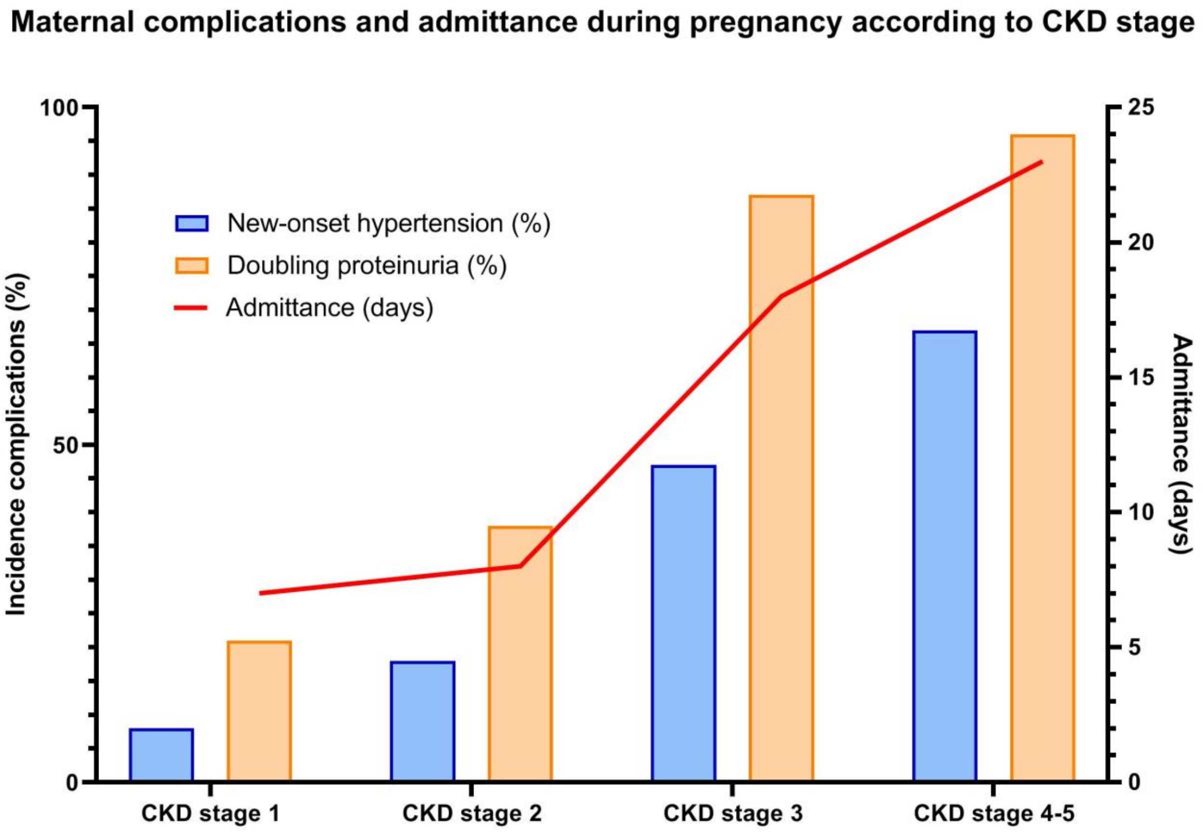

Figure S3: Kidney outcome six months postpartum according to prepregnancy CKD stage.

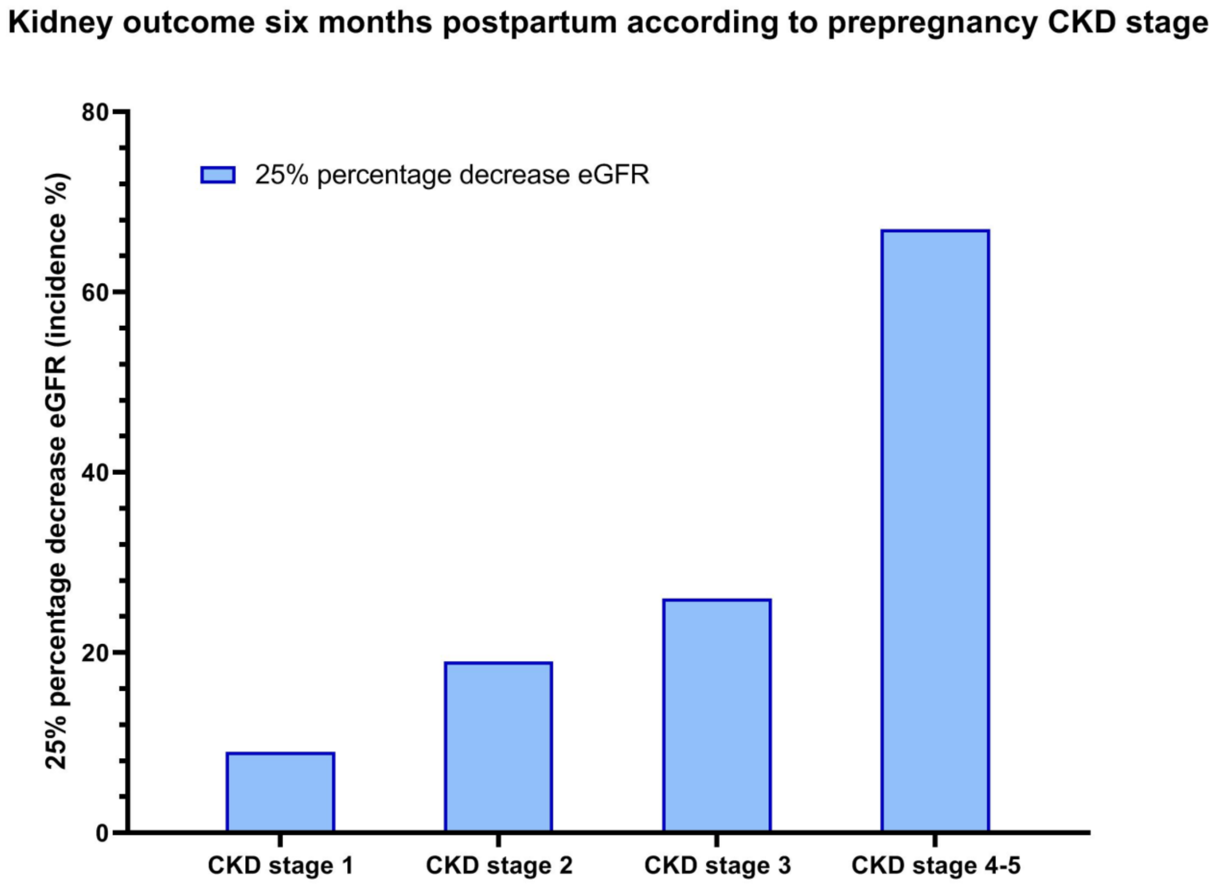

Table S1. Considerations for use of antihypertensive agents during lactation.

Medication	Trackable in breast milk; Relative Child Dose (RCD)	Medication effect in neonate	(Dis)advantages and considerations	Advice during lactation
Methyldopa	Yes, minimal	None	Safe. Association with postpartum depression, less potent. Dose thrice daily.	Decrease postpartum. Do no start postpartum.
Calciumantagonists				
Nifedipine	RCD 0.1-2.3%	None, also no growth restriction	Safe.<br>Advantage: 1-2 dd dosing	Good choice, certainly in women with sub-Saharian or Caribean origin
Amlodipine	Not or barely trackable	None	Safe. Advantage: 1dd dosing, few side-effects	Good choice
Beta blocking agents				
Labetalol	RCD <0.1%	None	Much experience. Safe. 2-3dd dosing	Good choice
Propranolol	RCD 0.1-0.9%	None	Safe. 2-3dd dosing	Good choice
Pindolol	RCD 0.36	None	1-2dd dosing. Few data, probably safe.	Good choice
Metoprolol	RCD <0.1%, does not accumulate	None	Safe. 1dd dosing	Good choice
Atenolol<br>Sotalol	RCD 6-19%<br>RCD 22%<br>Both: risk of accumulation, most time slower than therapeutic child dose	Hypoglycemia, hypotension, Bradycardia (rare)	Both: be careful with premature born infants <2-3 months old: monitoring for beta blocking effects	Find alternative
Alfa blocking agents (e.g. doxazosine)	Yes	None, but very few data	Very few data. Doxazosine up to 4mg gives very low concentrations in breast milk and should have no effects on the neonate	Find alternative because of lack of data
Diuretics				
Furosemide	Yes	None	Few data. Fluid depletion has negative effect on breast milk production	Avoid as much as possible, consider alternative
Hydrochlorothiazide	Yes, RCD 2%	None	Fluid depletion has negative effect on breast milk production. Dosages up to 50 mg/day are acceptable.	Safe up to 50 mg/day; agent of choice when indication for diuretic agent

Chloorthalidone	Yes, RCD 6%. Risk of accumulation. Slow clearance.	Possible accumulation in neonate and premature born infant	Possibly inhibits breast milk production. Few data.	Avoid as much as possible because of possible disadvantages
Amiloride	Yes	Unknown	No data. Might inhibit breast milk production.	Avoid as much as possible, risk for neonate unknown.
Triamterene	Yes	Unknown	No data	Avoid as much as possible, risk for neonate unknown
Spironolactone	RCD 0.2% estimated	Risk of gynecomasty small	Risk of inhibition of milk productions seems small in low doses. Few data.	Use with strict indication
Renin aldosterone inhibitors (RASI)	Yes, passes minimally in milk. RCD <5%	None<br>.	Preference for enalapril (up to 20 mg/day, 1-2 dd dosing).<br>Seems less effective in women with sub-Saharian or Caribean origin.<br>Be careful in premature born infants, high dose and neonates with kidney of liver function disorders	Good choice, certainly in diabetic nephropathy, proteinuria (≥0.5g/24h or ACR ≥30 mg/mmol) and women who used RASI before pregnancy.
Aldosteron receptor blocking agents	Yes, probably	Unknown	No/insufficient data about safety	Find alternative because of lack of data
Renin inhibitors	Unknown	Unknown	No/insufficient data about safety	Find alternative because of lack of data

Relative child dose (RCD): relationship between estimated dose which takes the child by breast milk and the dose taken by the mother. An RCD <10% is thought to be safe.
